# Insecticide resistance by a host-symbiont reciprocal detoxification

**DOI:** 10.1038/s41467-021-26649-2

**Published:** 2021-11-05

**Authors:** Yuya Sato, Seonghan Jang, Kazutaka Takeshita, Hideomi Itoh, Hideaki Koike, Kanako Tago, Masahito Hayatsu, Tomoyuki Hori, Yoshitomo Kikuchi

**Affiliations:** 1 Environmental Management Research Institute, National Institute of Advanced Industrial Science and Technology (AIST), Tsukuba Center, 305-8569 Tsukuba, Japan; 2 Graduate School of Agriculture, Hokkaido University, 060-8589 Sapporo, Japan; 3 Faculty of Bioresource Sciences, Akita Prefectural University, 010-0195 Akita, Japan; 4 Bioproduction Research Institute, AIST, Hokkaido Center, 062-8517 Sapporo, Japan; 5 Bioproduction Research Institute, AIST, Tsukuba Center, 305-8566 Tsukuba, Japan; 6 Institute for Agro-Environmental Sciences, National Agriculture and Food Research Organization (NARO), 305-8604 Tsukuba, Japan

**Keywords:** Evolutionary ecology, Coevolution, Symbiosis, Entomology

## Abstract

Insecticide resistance is one of the most serious problems in contemporary agriculture and public health. Although recent studies revealed that insect gut symbionts contribute to resistance, the symbiont-mediated detoxification process remains unclear. Here we report the in vivo detoxification process of an organophosphorus insecticide, fenitrothion, in the bean bug *Riptortus pedestris*. Using transcriptomics and reverse genetics, we reveal that gut symbiotic bacteria degrade this insecticide through a horizontally acquired insecticide-degrading enzyme into the non-insecticidal but bactericidal compound 3-methyl-4-nitrophenol, which is subsequently excreted by the host insect. This integrated “host-symbiont reciprocal detoxification relay” enables the simultaneous maintenance of symbiosis and efficient insecticide degradation. We also find that the symbiont-mediated detoxification process is analogous to the insect genome-encoded fenitrothion detoxification system present in other insects. Our findings highlight the capacity of symbiosis, combined with horizontal gene transfer in the environment, as a powerful strategy for an insect to instantly eliminate a toxic chemical compound, which could play a critical role in the human-pest arms race.

## Abstract (web summary)

Insect acquisition of insecticide resistance represents a serious problem for agriculture. Here, authors reveal an insect symbiotic bacteria that degrades insecticide fenitrothion into a non-insecticidal but bactericidal compound, which is subsequently excreted by the insect host.

## Introduction

Insects live in a world abounding with toxic compounds such as plant toxins and man-made pesticides. To overcome these toxins, herbivorous insects have evolved elaborate mechanisms for their detoxification^1–5^. Toxin resistance has brought insects to success in the terrestrial ecosystem, while at the same time, insecticide resistance is one of the most serious problems in contemporary agriculture and public health^2,6,7^. Although the resistance mechanisms are often encoded by the insects’ own genomes, recent studies revealed that in many insects, specific gut microorganisms also contribute to toxin resistance by degrading the chemical compounds^8,9^.

Many insects possess symbiotic bacteria in the bacteriocytes or the gut, wherein symbionts play pivotal metabolic roles such as the provision of essential amino acids, supplementation of vitamins, and digestion of indigestible food materials, such as plant cell walls^10–12^. In addition to the nutritional contribution, recent studies have revealed that symbiotic bacteria also confer other functions, including heat tolerance, parasite or pathogen resistance, body coloration, as well as toxin degradation^13,14^. However, how host–symbiont metabolic interactions play a role in these different symbiont-mediated ecological traits is poorly investigated. Here we report the in vivo detoxification process of the insecticide fenitrothion by gut symbionts in the bean bug *Riptortus pedestris*, revealing that a reciprocal host–symbiont detoxification of the insecticide and its bactericidal degradation-product is pivotal to maintain the stable association and thus the efficient detoxification.

**Fig. 1:**
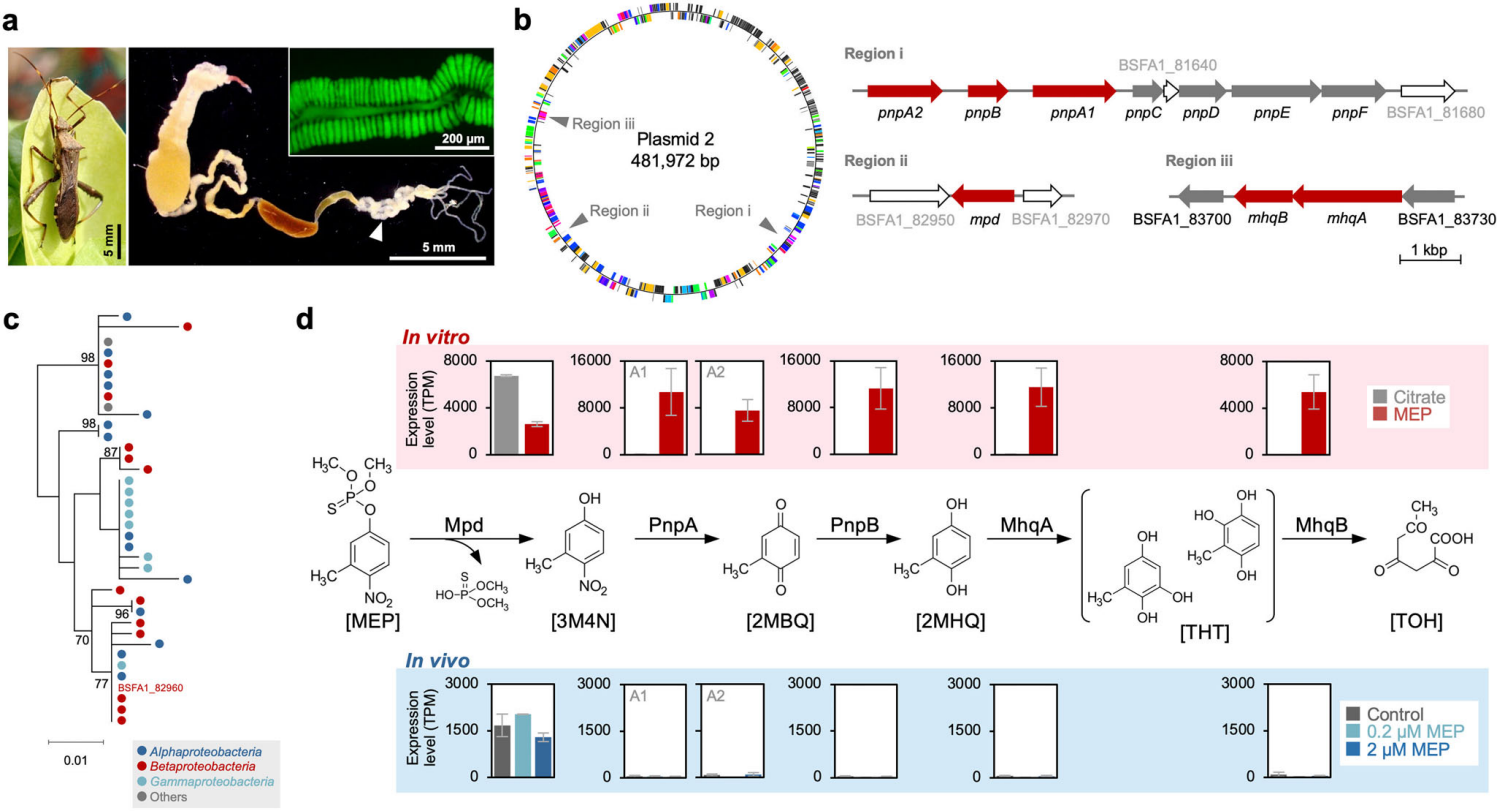
MEP-degrading genes are differentially expressed in cultured and gut-colonizing symbiont cells. **a** A male of *Riptortus pedestris* and its dissected gut. An arrow indicates symbiotic organ with numerous crypts. The inset is a fluorescence microscopy image of the crypts infected with a GFP-labeled MEP-degrading symbiont. **b** MEP-degrading genes encoded in plasmid 2 of the symbiont strain SFA1. **c** A phylogenetic tree of *mpd* (292 aa), showing frequent horizontal gene transfer of the MEP-degrading gene. See also Supplementary Fig. 3. **d** Gene expression patterns of the MEP-degrading genes in in vitro and in vivo conditions. Mean ± SEM of three replicates are indicated. Abbreviations of chemical compounds: MEP O,O-dimethyl O-[4-nitro-m-tolyl] phosphorothioate or fenitrothion, 3M4N 3-methyl-4-nitrophenol, 2MBQ 2-methyl-1,4-benzoquinone, 2MHQ 2-methylhydroquinone, THT trihydroxytoluene, TOH 2,4,6-trioxoheptanoate.

The bean bug *R. pedestris*, a serious pest of leguminous crops in Eastern Asia^15^, acquires a specific bacterial symbiont of the genus *Burkholderia* from the soil every generation and harbors 10^7^–10^8^ cells of these bacteria in midgut crypts (Fig. 1a)^16,17^. The *Burkholderia* symbiont contributes to the recycling of the host’s metabolic wastes, which benefits growth and reproduction of the bean bug host^18^. In addition, some symbiont strains are capable to degrade the organophosphorous insecticide fenitrothion (O,O-dimethyl O-[4-nitro-m-tolyl] phosphorothioate, or MEP). Colonization of the midgut crypts with such a strain results in the instant development of MEP resistance in the host insect^19^. MEP, which inhibits acetylcholine esterases in arthropods and exhibits both oral and percutaneous arthropod-specific toxicities, is one of the most popular organophosphorous insecticides used worldwide^20^. Since the degrading symbiont strains can utilize MEP as a carbon source^21,22^, spraying of MEP enriches degrading symbionts in soil, leading to an enhanced infection of the bean bug with MEP-degrading symbionts^23^. The symbiont-mediated resistance is effective for oral and cuticle treatments with MEP^19^.

Here, using transcriptomics and reverse genetics, we report the in vivo detoxification process of the insecticide fenitrothion by gut symbionts in the bean bug, revealing that a reciprocal host–symbiont detoxification of the insecticide and its bactericidal degradation product is pivotal to maintain the stable association, thereby enabling efficient detoxification.

## Results and discussion

### MEP-degradation pathway in the *Burkholderia* symbiont

To clarify the genetic basis of the symbiont-mediated MEP resistance, we first determined the whole-genome sequence of the typical MEP-degrading *Burkholderia* symbiont, strain SFA1^19^. The genome of SFA1 was 9.5 Mb in size, consisting of three circular chromosomes and five plasmids (Supplementary Fig. 1). This symbiont possessed MEP-degrading gene clusters previously reported in *Pseudomonas* and another *Burkholderia* species on the 0.48-Mb plasmid 2 (Fig. 1b, Supplementary Fig. 2). The phylogeny of bacterial *mpd* genes as well as other genes of the Mhq pathway (Fig. 1c, Supplementary Fig. 3a–d) was inconsistent with the phylogeny of the corresponding bacteria in which the genes were present (Supplementary Fig. 3e), strongly suggesting that they are horizontally transmitted among a broad range of Gram-negative bacteria, and that the *Burkholderia* symbiont acquired the MEP-degrading ability via the horizontal transmission of this plasmid. Cocultivation tests demonstrated that the plasmid is indeed frequently transferred from the SFA1 donor to nondegrading symbionts (Supplementary Fig. 4a–c), confirming its mobility between bacteria. Furthermore, the newly emerged degrading symbiont colonized the gut of the bean bug and conferred MEP resistance on the host insect (Supplementary Fig. 4d and e).

The genomic data suggested that MEP can be assimilated by strain SFA1 via two metabolic pathways branching at 2-methylhydroquinone: the *para*-nitrophenol-reductase (Pnp) pathway and the methylhydroquinone-metabolizing enzyme (Mhq) pathway (Supplementary Fig. 2a). When the symbiont was cultured in a minimal medium containing MEP as the sole carbon source, the Mhq-pathway genes, as well as the upstream methyl parathion-degrading enzyme (*mpd*) and *pnpAB* genes, were highly expressed (Fig. 1d, Supplementary Fig. 2b, c). This indicated that SFA1 assimilates MEP mainly via the Mhq pathway.

### First catabolic step of MEP degradation is critical and sufficient for the symbiont-mediated insecticide resistance

**Fig. 2:**
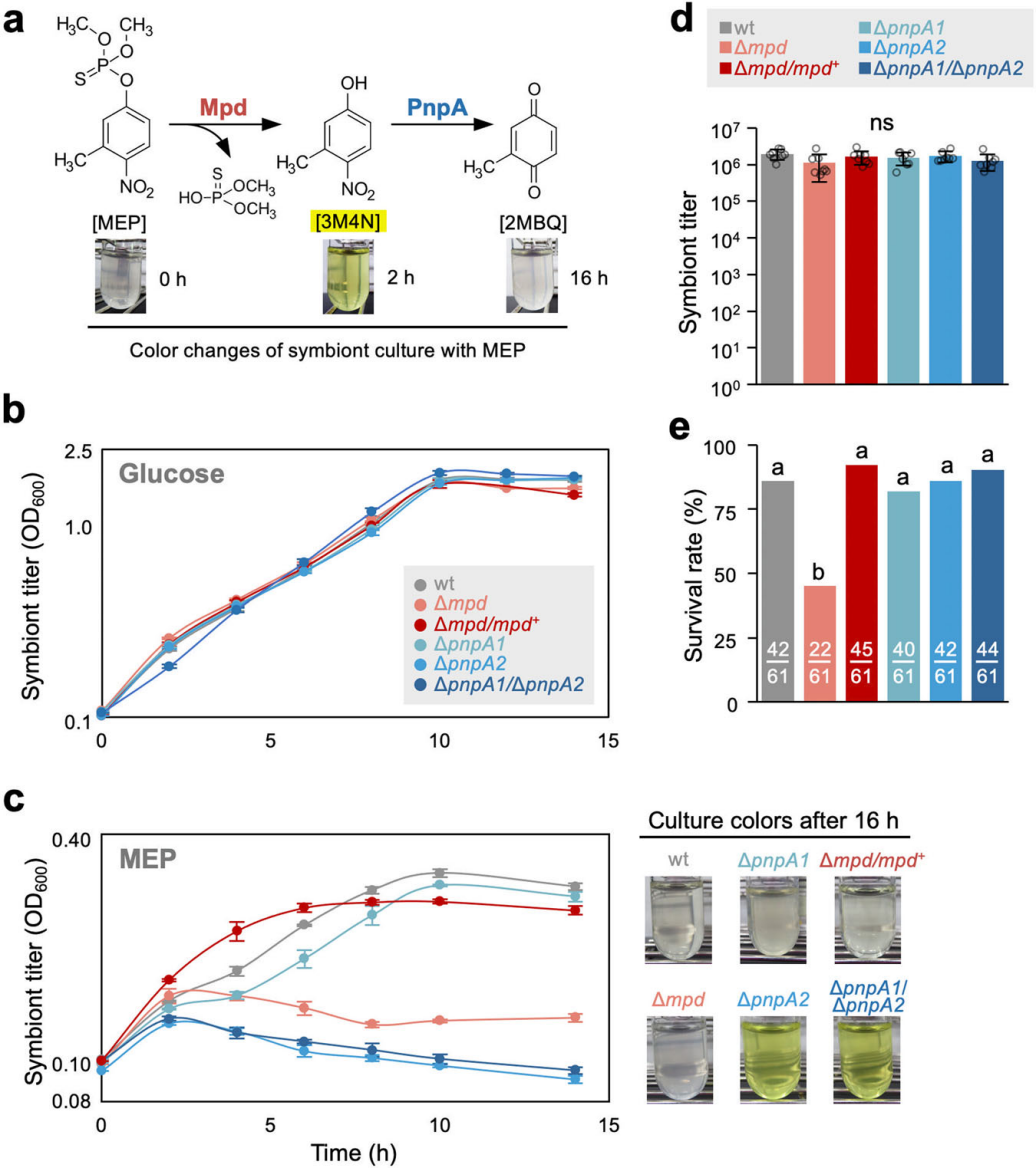
The first catabolic step of MEP degradation is critical for the symbiont-mediated insecticide resistance. **a** Partial MEP-degradation pathway and corresponding color changes in wild-type SFA1 culture with MEP. **b**, **c** Growth rates of wild-type SFA1, *mpd* (Δ*mpd*) and *pnpA* gene-deletion mutants (Δ*pnpA1*, Δ*pnpA2*, and Δ*pnpA1/*Δ*pnpA2*), and *mpd*-complement mutant (Δ*mpd*/*mpd*^+^) in (**b**) glucose and (**c**) MEP medium. Mean ± SD of three replicates are indicated. Photos of culture media (16 h after inoculation) are shown on the right side of (**c**). The medium of Δ*pnpA2* and Δ*pnpA1*/Δ*pnpA2* became yellow because of 3M4N accumulation. **d** Colonization titers of the wild-type and mutant strains in midgut crypts. Symbiont titer between the groups was calculated by qPCR of the *dnaA* gene of the symbiont. Mean ± SD of eight replicates are indicated. ns indicates statistical insignificance (two-sided Mann–Whitney *U* test after Bonferroni correction). **e** Survival rates after MEP treatment in insects infected with either wild type or mutants. Number of replicates are depicted on the bars. Different letters indicate statistically significant differences (*P* < 0.05, two-sided Fisher’s exact test after Bonferroni correction).

We then investigated the expression levels of the MEP-degrading genes in the SFA1 bacteria colonizing the midgut crypts of the insect. Unexpectedly, only the first gene, *mpd*, was highly expressed, while all the downstream genes were nearly silent (Fig. 1d, Supplementary Fig. 2b). Moreover, the expression level of the genes was not affected by MEP treatment of the insects. The *mpd* gene was also highly expressed in bacterial culture grown without MEP (Fig. 1d), indicating that it is constitutively expressed in SFA1. Previous studies on *Pseudomonas* strains reported that Mpd is a membrane protein and the first step of the MEP degradation occurs in the periplasmic space^24–27^. While MEP is highly toxic to insects, the degradation product, 3-methyl-4-nitrophenol (3M4N, a yellow-colored phenolic compound) (Fig. 2a), is nontoxic (Supplementary Fig. 5a). These results strongly suggested that the MEP resistance is conferred exclusively by the expression of *mpd* in the gut and by the Mpd-mediated conversion of MEP to 3M4N.

To confirm this point, the *mpd* and *pnpA* genes were deleted and MEP-exposure tests were performed with insects infected with the mutants. PnpA, degrading 3M4N to 2-methyl-1,4-benzoquinone, is encoded in strain SFA1 by two homologous genes, *pnpA1* and *pnpA2* (Fig. 1b, d, Supplementary Fig. 2), which were both deleted. These gene-deletion mutants, as well as wild-type SFA1, grew well in a minimal medium containing glucose as the sole carbon source (Fig. 2b). However, the Δ*mpd* and Δ*pnpA2* single mutants, and the Δ*pnpA1/*Δ*pnpA2* double mutant, but not the Δ*pnpA1* single mutant, were not able to grow in a minimal medium containing MEP as the sole carbon source (Fig. 2c), confirming the involvement of *mpd* and *pnpA2* in the MEP degradation by SFA1. All the deletion mutants colonized well the midgut crypts of the bean bug when insects were reared in the absence of MEP (Fig. 2d). However, when the infected insects were treated with MEP, those harboring the Δ*mpd* mutant showed a significant reduction of the survival rate but not those harboring the Δ*pnpA1*, Δ*pnpA2*, or Δ*pnpA1/*Δ*pnpA2* mutants (Fig. 2e). A genetically complemented mutant of *mpd*, Δ*mpd*/*mpd*^*+*^, restored the growth ability in MEP medium (Fig. 2c) and the ability for conferring MEP resistance in the bean bug host (Fig. 2e). Together, these results demonstrated that (1) the MEP-degradation process and gene expression pattern in SFA1 is remarkably different between in vitro and in vivo conditions, (2) only *mpd* is highly expressed inside the insect gut, and (3) *mpd* is necessary and sufficient for MEP resistance in the insect host.

### Insecticide-degradation product 3M4N is highly bactericidal

**Fig. 3:**
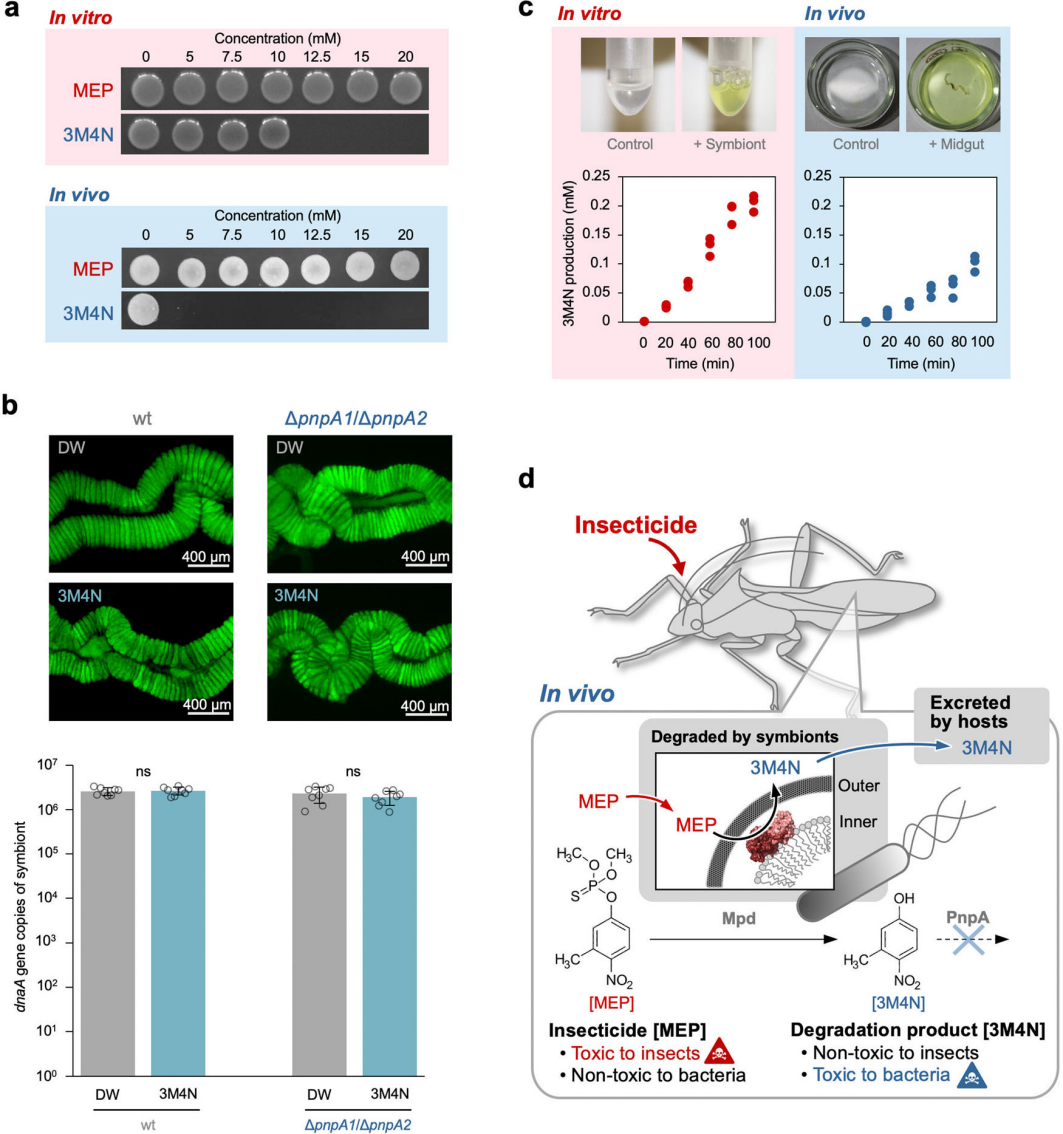
The insecticide-degradation product, 3M4N, is highly bactericidal but efficiently eliminated by the host from the gut symbiotic organ. **a** Bactericidal abilities of MEP and 3M4N on cultured SFA1 cells (in vitro) and midgut symbiont cells (in vivo). **b** Effects of 3M4N on crypt-colonizing symbiont population in the bean bug gut. Insects infected with SFA1 (wt) and its Δ*pnpA1*/Δ*pnpA2* mutant were fed on soybean seeds and either distilled water (DW) or DW containing 5 mM 3M4N. Symbiont titer between the groups was compared by qPCR of the *dnaA* gene of the symbiont. Mean ± SEM of eight replicates are indicated. ns indicates statistical insignificance (two-sided Fisher’s exact test). Fluorescent microscopy images of the midgut crypts colonized by GFP-labeled SFA1 (wt) and Δ*pnpA1*/Δ*pnpA2* are shown on top of the bar graph. Fluorescent microscopy was independently repeated 10 times. **c** MEP degradation and 3M4N production in the gut harboring the MEP-degrading symbiont SFA1. The degradation rate is compared with that of SFA1 grown in culture. The data represent the results of three independent experiments. **d** A schematic illustration of the MEP detoxification governed by host–symbiont mutualistic interactions.

Notably, the MEP medium of Δ*pnpA2* and Δ*pnpA1/*Δ*pnpA2* cultures became yellow in color due to the accumulation of 3M4N (Fig. 2c). When transferred to MEP medium after culturing in nutrient medium containing citrate as a carbon source, the growth of wild-type SFA1 is halting by 10–20 h before resuming (Supplementary Fig. 5b–d). Since *pnpA* expression depends on its substrate 3M4N^28,29^, this long growth lag is probably due to the accumulation of 3M4N in the cultures, as suggested by their transient yellowish color (Fig. 2a, Supplementary Fig. 5c), until induction of the *pnpA* expression (Supplementary Fig. 5e). Moreover, it could also indicate that this compound is toxic to the symbiotic bacteria. Indeed, when cultured SFA1 cells were incubated either with MEP or 3M4N and subsequently spotted on agar plates, 3M4N but not MEP showed bactericidal activity (Fig. 3a). Our previous study reported that the symbionts in the midgut crypts show a thinner cell wall compared with the cultured bacteria and that they are highly sensitive to various stresses such as detergents, proteases, and cell-surface-attacking antimicrobial peptides^18^. The spot test revealed that, while MEP is still nontoxic, 3M4N shows a drastically higher toxicity to SFA1 cells isolated from midgut crypts than to SFA1 grown in culture (Fig. 3a, b).

### Bactericidal product 3M4N is efficiently eliminated by the host from the gut symbiotic organ

The imbalanced expression pattern of *mpd* and *pnpA* in the midgut crypts, i.e., constitutive *mpd* expression and little *pnpA* expression, is expected a priori to cause an accumulation of 3M4N in the midgut, which could lead to a severe effect on the symbiont population. However, the symbiont titer in the insects was not affected by 3M4N feeding (Fig. 3b). Furthermore, even the Δ*pnpA1*/Δ*pnpA2* mutant, which lacked the 3M4N-degrading enzyme and was thus susceptible to the compound (Fig. 2c), was also not affected by 3M4N feeding in the midgut crypts (Fig. 3b), strongly suggesting nonaccumulation of 3M4N in the symbiotic organ. When dissected midguts infected with SFA1 were knotted with a nylon wire at the anterior and posterior portions to avoid bacterial leakage (Supplementary Fig. 6a) and incubated in a phosphate buffer containing 2 mM MEP, 3M4N accumulated in the solution (Fig. 3c). This finding indicates that MEP permeates into the crypt lumen and is degraded by the symbiont and that subsequently its degradation product 3M4N is actively excreted from the midgut to the outside (i.e., the hemolymph in the living insect), even though the transportation system of 3M4N through crypt epithelia remains unclear. The low expression of the 3M4N-inducible *pnpA2* gene in the midgut (Fig. 1d) supports this conclusion. The exclusion ability of 3M4N by the midgut was not affected by the presence of trehalose that is known as the major sugar in insect hemolymph (Supplementary Fig. 6b). Although MEP degradation was accomplished in midgut crypts (Fig. 3d), the degradation activity per symbiont cell was approximately ten times lower in the midgut cells than that in cells in culture (Supplementary Fig. 6c). This could be because (1) the expression activity of *mpd* or its protein product is low in the gut; (2) the transport systems of MEP and 3M4N in the gut epithelial cells limit the degradation efficiency; or (3) symbiont cells are compacted in the gut crypts, which reduces the available surface area of the symbiont for uptake and also the degradation efficiency. When feces of bean bugs fed with water containing 3M4N were analyzed by LC–MS, the compound was detected in the feces (Supplementary Fig. 7), suggesting that 3M4N is not further metabolized in the bean bug host but excreted in unchanged form, although this point still needs further confirmation.

### Host–symbiont reciprocal detoxification

Taken together, we conclude that the symbiont-mediated insecticide detoxification is accomplished by an elaborate host–symbiont collaboration. Symbiotic bacteria actively degrade the insecticidal (but not bactericidal) MEP, and in return, the host insect immediately excretes its bactericidal (but not insecticidal) degradation product 3M4N (Fig. 3d). Hence, this coordinated detoxification enables the maintenance of the symbiosis, and thereby sustains high degradation activity, even under insecticide stress. Host–symbiont metabolic integration, wherein both the host and the bacterial symbiont contribute to metabolic pathways, is a common feature in many symbiotic interactions of plant-sucking and blood-feeding insects with some biosynthetic steps performed by symbiont enzymes and others by host-encoded enzymes^10–12^. This study demonstrates that, in the detoxification symbiosis, the host–symbiont metabolic integration is also pivotal.

It should be noted that the symbiont-mediated detoxification process of MEP is remarkably analogous to another known MEP-detoxification mechanism in insects that is mediated by an insect-encoded glutathione-S transferase (GST) (Supplementary Fig. 8). Insect GSTs detoxify MEP by conjugating glutathione, while the symbiont Mpd hydrolyzes MEP, and their respective products, 5-*S*-glutathionyl-1-methyl-2-nitrobenzene and 3M4N, are subsequently eliminated from the insect cells and tissues. Constitutive high expression of GSTs, reported in MEP-resistant insects^30^, can thus be mimicked functionally and mechanistically in insects lacking such a GST by a MEP-degrading symbiont that is constitutively expressing *mpd*, without the need of any mutation in the insects’ own genomes to evolve a MEP-modifying GST. Another notable point is that gut symbionts can rapidly gain the detoxification ability through horizontal gene transfer. Diverse bacteria encode insecticide-degrading genes on plasmids^8^, and in *Burkholderia* species, MEP-degrading plasmids can be acquired or lost dynamically, depending on the presence of the insecticide in the environment^21^ (Supplementary Fig. 4). Thus, highly flexible symbiont-mediated detoxification mechanisms may play a critical role in the evolutionary arms race and integrated coevolution of toxin-mediated relationships such as plant–herbivore and human–pest antagonisms.

## Methods

### Insects and bacteria

Bean bugs were reared in petri dishes (90 mm in diameter and 20-mm high) at 25 °C under a long-day regimen (16-h light, 8-h dark) and fed with soybean seeds and distilled water containing 0.05% ascorbic acid (DWA). *Burkholderia* symbiont strain SFA1^19^, a MEP-degrading strain conferring MEP resistant in the bean bug, and its GFP-(green fluorescent protein) labeled derivative, strain SJ586, were used in this study. The symbiont was cultured at 30 °C on YG medium (0.5% yeast extract, 0.4% glucose, and 0.1% NaCl). The GFP-labeled strain was constructed by the Tn7 mini-transposon system, as previously described^31^.

### Genome sequencing

DNA was extracted from cultured cells of strain SFA1 by the phenol–chloroform extraction as previously described^32^. The DNA library for Illumina short reads (the mean insert size: 500 bp) was constructed by using the Covaris S2 ultrasonicator (Covaris) and the KAPA HyperPrep Kit (Kapa Biosystems). For the library construction for Nanopore long reads, Native Barcoding Expansion (EXP-NBD104, Oxford Nanopore Technologies) and the Ligation Sequencing Kit (SQK-LSK109, Oxford Nanopore Technologies) were used. The genome sequencing was performed with NextSeq using the 2 × 151-bp protocol (Illumina) and GridION using an R9.4.1 flow cell (Oxford Nanopore Technologies). The Illumina short reads were processed by using Sickle Ver 1.33 (available at https://github.com/najoshi/sickle) for removing the low-quality and shorter reads. After processing the Nanopore long-reads with Porechop Ver 0.2.3 (available at https://github.com/rrwick/Porechop) and Filtlong Ver 0.2.0 (available at https://github.com/rrwick/Filtlong), error correction was performed by using Canu Ver 1.8^33^. These processed short- and long reads were assembled by using Unicycler Ver 0.4.7^34^, resulting in the eight circular replicons (Supplementary Fig. 1). The assembled genome was annotated by DFAST Ver 1.1.0^35^. After the homology searches of the protein sequences by blastp 2.5.0 + ^36^ against the COG database (PMID: 25428365), circular replicons were visualized with circos v 0.69-8^37^. The chromosomes and plasmids were assigned according to the genome of *Caballeronia* (*Burkholderia*) *cordobensis* strain YI23^38^.

### Phylogenetic analysis

Nucleotide sequences of 16 S rRNA gene of representative *Burkholderia* spp. and outgroup species were aligned by using SINA v1.2.11^39^. Protein sequences of MEP-degrading genes (*mpd*, *pnpB*, and *mhqA*) and a plasmid-transfer gene (*traH*) on plasmid 2 were subjected to the blastp search against the *nr* database (downloaded in Jul. 2019) and top ~30 hit sequences were retrieved for each gene. Multiple sequencing alignments of each gene were constructed with L-INS-I of mafft v7.407^40^. Gap-including and ambiguous sites in the alignments were then removed. Unrooted maximum-likelihood (ML) phylogenetic trees were reconstructed with RAxML v8.2.3^41^ using the GTR + Γ model (for 16 S rRNA gene) or the LG + Γ model^42^ (for other genes). The bootstrap values of 1000 replicates for all internal branches were calculated with a rapid bootstrapping algorithm^43^.

### Preparation of SFA1 cultures for RNA-seq

*Burkholderia* symbiont SFA1 was precultured in minimal medium (20 mM phosphate buffer [pH 7.0], 0.01% yeast, 0.1% (NH_4_)_2_SO_4_, 0.02% NaCl, 0.01% MgSO_4_⋅7H_2_O, 0.005% CaCl_2_⋅2H_2_O, 0.00025% FeSO_4_⋅7H_2_O, and 0.00033% EDTA⋅2Na) containing 1.0 mM of MEP on a gyratory shaker (210 rpm) at 30 °C overnight, and subcultured in newly prepared MEP-containing minimal medium under the same conditions for 5 h. As a control, SFA1 was precultured in minimal medium containing 0.1% citrate overnight, and then the overnighter was subcultured in a newly prepared citrate-containing minimal medium under the same conditions for 10 h. The culture was mixed with an equal amount of RNAprotect Bacteria Regent (Qiagen, Valencia, CA, USA), then centrifuged to harvest the cells for the RNA-seq analysis.

### Preparation of midgut symbiont cells for RNA-seq

The oral administration of the symbiont strain SFA1 was performed as described^19,44^. The symbiont was inoculated to 2^nd^ instar nymphs, and three days after molting to the 3rd instar, nymphs were transdermally administered with 1 µl of 0.2 µM or 20 µM of MEP (dissolved in acetone). One- or three days after the treatment, insects were dissected and the crypt-bearing symbiotic gut region was subjected to the RNA extraction and RNA-seq analysis. As a control, untreated insects were analyzed.

### RNA-seq analysis

Total RNA was extracted from triplicate samples from cultures by the hot-phenol method as previously described^45^ or from the midgut symbiont cells by using RNAiso Plus (Takara Bi, Kusatsu, Shiga, Japan) and the RNeasy mini kit (Qiagen). The extracted total RNA was purified by phenol–chloroform extraction and digestion by DNase (RQ1 RNase-Free DNase, Promega, Fitchburg, WI, USA) and repurified by using a RNeasy Mini Kit. The mRNA in the samples was further enriched by the RiboMinus Transcriptome Isolation Kit bacteria (Thermo Fisher Scientific, Waltham, MA, USA) and the RiboMinus Eukaryote Kit for RNA-Seq (Thermo Fisher Scientific), and purified by using an AMPure XP kit (Beckman Coulter, Brea, CA, USA). The cDNA libraries were constructed from approximately 100 ng of rRNA-depleted RNA samples by the use of a NextUltraRNA library prep kit (New England Biolabs, Ipswich, MA, USA). Size selection of cDNA (200–300 bp) and determination of the size distribution and concentration of the purified cDNA samples were performed as described previously^46^. In total, 21 cDNA libraries were constructed and sequenced by MiSeq (Illumina, Inc., San Diego, CA, USA). To ensure high sequence quality, the remaining sequencing adapters and the reads with a cutoff Phred score of 15 (for leading and tailing sequences, Phred score of >20) and a length of less than 80 bp in the obtained RNA-seq data were removed by the program Trimmomatic v0.30 using Illumina TruSeq3 adapter sequences for the clipping^47^. The remaining paired reads were analyzed by FastQC version 0.11.9 (http://www.bioinformatics.babraham.ac.uk/projects/fastqc/) for quality control, and Bowtie2 ver. 2.2.2^48^ for mapping on the symbiont genome (DDBJ/EMBL/GenBank accession: AP022305–AP022312). After the conversion of the output BAM files to BED files using the bamtobed program in BEDTools ver. 2.14.3^49^, gene expression levels were calculated in TPM (transcripts per kilobase million) values by using in-house scripts^46^.

### Gene deletion and complementation

MEP-degrading genes (*mpd*, *pnpA1*, and *pnpA2*) were deleted by the homologous-recombination-based deletion method using pK18mobsacB or pUC18, as previously described^50,51^. Primers used for the mutagenesis are listed in Supplementary Table 1. For *mpd* gene deletion, pK18mobsacB was used to construct a markerless mutant. For single deletion of *pnpA1* and *pnpA2* genes, pUC18 was used to substitute each gene locus with a kanamycin-resistance gene cassette. The double deletion of *pnpA1* and *pnpA2* genes was performed by substituting *pnpA2* gene locus with a tetracycline-resistance gene cassette in the *pnpA1*-deletion mutant. Gene complementation of *mpd* was also performed by homologous recombination using plasmid pUC18 with primers listed in Supplementary Table 1. To investigate growth profiles of the wild-type SFA1, the gene-deletion mutants (Δ*mpd*, Δ*pnpA1*, Δ*pnpA2*, and Δ*pnpA1/*Δ*pnpA2*), and the *mph*-complement mutant (Δ*mpd*/*mpd*^+^) in the MEP-containing minimal medium, the strains were precultured in minimal medium containing 1.0 mM MEP on a gyratory shaker (210 rpm) at 30 °C overnight, and then cultured in newly prepared MEP-containing minimal medium under the same condition. The growth of cultures was estimated by OD_600_ measurements. To confirm the basic growth abilities of the mutants, these bacterial strains were pre- and subcultured in minimal medium containing 0.1% glucose under the same conditions. These symbiont strains and mutants were inoculated to the bean bug as described above.

### Quantitative PCR

Symbiont titers in the midgut crypts were evaluated by quantitative PCR (qPCR) of bacterial *dnaA* gene copies. The qPCR was performed by using a KAPA SYBR Fast qPCR Master Mix (Kapa Biosystems) and the LightCycler 96 System (Roche Applied Science) with the following primers: BSdnaA–F (5′-AGC GCG AGA TCA GAC GGT CGT CGA T-3′) and BSdnaA–R (5′-TCC GGC AAG TCG CGC ACG CA-3′).

### MEP treatment of insects

MEP treatment of *R. pedestris* was performed as previously described^19^. Soybean seeds were dipped in 0.2 mM MEP for 5 s and dried at room temperature. In each clean plastic container, 15 individuals of 3^rd^-instar nymphs were reared on three seeds of the MEP-treated soybean and DWA at 25 °C under the long-day regime, and the number of dead insects was counted 24 h after the treatments. The survival rate of the insects was analyzed under Fisher’s exact test by use of the program R ver. 3.6.3 (available at https://www.R-project.org/). Multiple comparisons were corrected by the Bonferroni method.

### Bactericidal activities of MEP and its degradation product 3M4N

To measure bactericidal activities of MEP and 3M4N on cultured cells of SFA1, 10^4^ cells of log-phase growing bacteria were mixed with a defined concentration of MEP or 3M4N, and spotted on a YG agar plate. To measure the bactericidal activity against midgut crypt-colonizing cells, the symbiotic organs infected with SFA1 were dissected from 3^rd^-instar insects, homogenized in PBS, and purified by a 5-µm-size pore Syringe filter to harvest colonizing symbiont cells^50^. MEP or 3M4N was added to approximately 10^4^ cells of the harvested cells and spotted on a YG agar plate. Bactericidal activities of the chemical compounds were then checked in 24 h after incubation at 30 °C.

### HPLC detection of in vitro and in vivo MEP-degrading activities of the symbiont

To determine in vitro MEP-degradation activity, cultured cells of SFA1 were prepared as above, and 10^6^ cells were incubated at 25 °C in 200 µl of MEP solution (2 mM MEP in Tris-Hcl [pH 8.5] with 0.1% Triton X-100) in a 1.5-ml microtube. To determine in vivo MEP-degradation activity, the midgut of a 5^th^-instar insect infected with SFA1 was dissected, the posterior and anterior parts of the crypt-bearing symbiotic region were closed with 0.2-mm polyethylene fishline (Supplementary Fig. 6a), and incubated at 25 °C in 200 µl of the MEP solution. For the in vivo determination, 250 mM of trehalose, known as a major sugar source of insects’ hemolymph^52^, was added to the MEP solution to keep the tissue fresh. After incubation for different times, the reaction was stopped by adding 400 µl of methanol. After centrifugation, supernatants were subjected to high-performance liquid chromatography (HPLC) analyses to detect MEP and 3M4N, as previously reported^21^, and precipitated cells and tissues were subjected to DNA extraction and qPCR to estimate symbiont-cell numbers of each reaction.

### LC–ESI–MS detection of 3M4N in feces from 3M4N-fed insects

An insect-rearing system for feeding 3M4N and collecting feces is shown in Supplementary Fig. 7. Insects were fed with DW or DW containing 10 mM 3M4N in a plastic container, in which the solution supplier was covered by 0.5-mm mesh, so that insects were able to drink the solution by probing with their proboscis, but did not directly touch the solution by their legs or body. Twenty insects were reared per container and their feces were accumulated on the bottom of the container for five days. The collected feces (DW- or 3M4N-treated) were suspended in 1 ml of MilliQ water, and the water-soluble fractions were extracted by thorough vortexing. Solids and insoluble fractions were removed from the suspension by centrifugation and subsequent filtration using a cellulose-acetate membrane (Φ, 0.20 μm, ADVANTEC, Tokyo, Japan). The resultant fraction was diluted 10-fold by MilliQ water and analyzed by liquid chromatography–electrospray-ionization mass spectrometry (LC–ESI–MS) according to a previous report^53–55^. HPLC was performed using the Nexera X2 system (Shimadzu, Kyoto, Japan) composed of LC-30AD pump, SPD-M30A photodiode-array detector, and SIL-30AC autosampler. Develosil HB ODS-UG column (ID 2.0 mm × L 75 mm, Nomura Chemical Co., Ltd, Aichi, Japan) was employed with a flow rate of 0.2 mL/min. The following gradient system was used for analysis of metabolites: MilliQ water (solvent A) and methanol (solvent B), 90% A and 10% B at 0–5 min, linear gradient from 90% A and 10% B to 20% A and 80% B at 5–15 min, 20% A and 80% B at 15–20 min, and 90% A and 10% B at 20–25 min. Retention time of 3M4N standard reagent was 14.2 min. Electrospray-ionization mass spectrometry (ESI–MS) in positive and negative ion modes was simultaneously performed using amaZon SL (Bruker, Billerica, MA, USA). 3M4N (MW = 153.14) standard showed a clear peak in negative mode at *m/z* of 151.53.

### Reporting summary

Further information on research design is available in the Nature Research Reporting Summary linked to this article.

## Supplementary information

Supplementary Information

Peer Review File

Reporting Summary

## Data Availability

All relevant data, including qPCR, bacterial growth rate, and gene expression profiles of the insecticide-degradation enzymes, are available in the Figshare repository (10.6084/m9.figshare.16748203.v1). The annotated genome of the *Burkholderia* symbiont strain SFA1 has been deposited in the DDBJ/EMBL/GenBank nucleotide-sequence database under the accession numbers AP022305–AP022312 and the raw sequence data have been deposited in DRA under the accession number DRA009280. The assembled genome was annotated by using the COG database (PMID: 25428365), and the chromosomes and plasmids were assigned according to the genome of *Burkholderia* (*Caballeronia*) *cordobensis* strain YI23 (CP003087–CP003092 in the DDBJ/EMBL/GenBank nucleotide-sequence database). The RNA-seq nucleotide sequences reported in this study were deposited in the DDBJ/GenBank/EBI databases under the accession number DRA010054.
