# Supplementary material for: Insecticide resistance by a host-symbiont reciprocal detoxification
Source: Nat Commun. 2021 Nov 5;12:6432. doi: 10.1038/s41467-021-26649-2 (PMC8571283; doi:10.1038/s41467-021-26649-2)
Supplement: Supplementary file 1 — Supplementary Information [file 41467_2021_26649_MOESM1_ESM.pdf]

Supplementary Information

Insecticide resistance by a host-symbiont reciprocal detoxification

Sato, Jang *et al.*

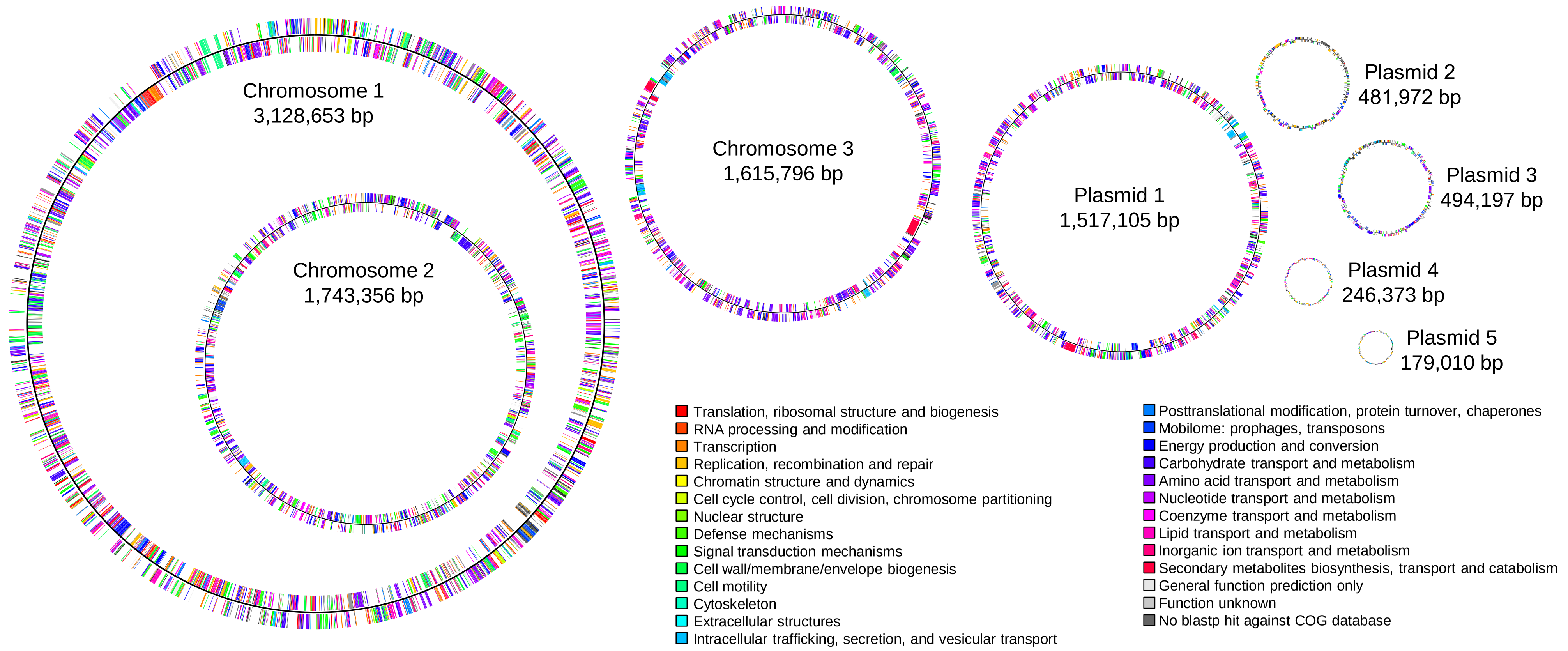

Supplementary Fig. 1 | The genome of *Burkholderia* symbiont strain SFA1. On the CDS circle, colors indicate functional categories as shown in the figure.

a

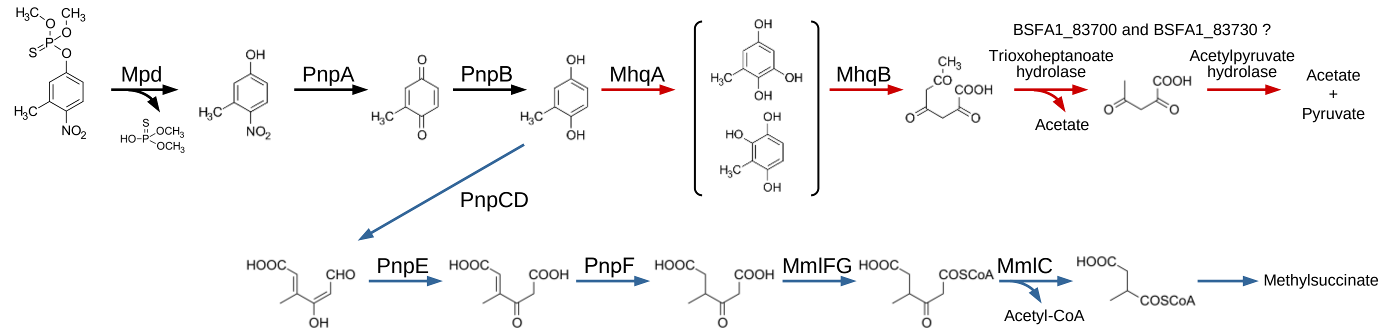

b

Gene	ID	Chromosome / Plasmid number	Gene expression level (TPM)						
			In vitro		*In vivo* (24 h)			*In vivo* (72 h)	
			Citrate	MEP	Control	0.2 µM MEP	2 µM MEP	Control	0.2 µM MEP
mpd	BSFA1_82960	Plasmid 2	6759	2718	1678	2032	1294	1467	1188
pnpA1	BSFA1_81620	Plasmid 2	32	9045	39	8	30	29	13
pnpA2	BSFA1_81600	Plasmid 2	54	10748	51	50	40	39	65
pnpB	BSFA1_81610	Plasmid 2	60	13924	78	10	103	44	46
mhqA	BSFA1_83720	Plasmid 2	67	14623	63	14	54	30	34
mhqB	BSFA1_83710	Plasmid 2	37	6784	99	13	60	26	26
hydrolase	BSFA1_83700	Plasmid 2	10	3340	24	42	16	0	33
5-oxopent-3-ene-1,2,5-tricarboxylate decarboxylase	BSFA1_83730	Plasmid 2	40	13617	88	77	38	31	5
pnpC	BSFA1_81630	Plasmid 2	42	12775	113	71	74	42	39
pnpD	BSFA1_81650	Plasmid 2	41	3759	33	27	0	13	30
pnpE	BSFA1_81660	Plasmid 2	4	196	8	21	11	19	12
pnpF	BSFA1_81670	Plasmid 2	9	194	30	11	65	18	0
mmlC	BSFA1_71580	Plasmid 1	4	9	2	16	19	0	4
mmlF	BSFA1_11210	Chromosome 1	171	81	54	31	29	65	71
mmlG	BSFA1_11220	Chromosome 1	182	139	9	144	37	57	64

c

Gene	ID	Chromosome / Plasmid number	Gene expression level (TPM)						
			In vitro		*In vivo* (24 h)			*In vivo* (72 h)	
			Citrate	MEP	Control	0.2 µM MEP	2 µM MEP	Control	0.2 µM MEP
pnpC	BSFA1_47960	Chromosome 3	26	48	71	52	16	46	23
pnpD	BSFA1_47970	Chromosome 3	14	23	21	10	32	21	5
pnpE	BSFA1_47980	Chromosome 3	7	10	52	12	14	11	17
pnpF	BSFA1_47990	Chromosome 3	44	35	41	10	15	11	15
pnpF	BSFA1_53550	Chromosome 3	5	3	38	10	0	16	10

Common · Mhq pathway · Pnp pathway

Gene expression (TPM): 20,000 · 2,000 · 0

Supplementary Fig. 2 | MEP-degradation pathways inferred from the SFA1 genome. a, The Pnp pathway (blue arrows) was reported in the MEP-degrading *Pseudomonas* sp. WBC-3[28] and the Mhq pathway (red arrows) was reported in the MEP-degrading *Burkholderia* sp. NF100[54]. **b,** Gene expression levels of the MEP-degrading genes. All expression values are mean from three replicates. Note that the Mhq pathway is mainly used in SFA1 when MEP is added as the sole carbon source in culture. In the Mhq pathway, the last two steps of the ring cleavage process are still undetermined. Considering a previous report[55] and our RNA-seq data, BSFA1_83700 and BSFA1_83730, which are adjacent to the *mhqA* and *mhqB* genes, are likely involved in the ring cleavage process. Low expression levels (transcripts per million [TPM] values <177) of *mmlC* (BSFA1_71580 encoding 4-methyl-3-oxoadipyl-CoA thioesterase, Chromosome 1) and *mmlFG* (BSFA1_11210 and BSFA1_11220 encoding 4-methyl-3-oxoadipate CoA-transferase alpha- and beta-subunit, respectively; Plasmid 1) suggested that they are not involved in the MEP degradation. **c,** *pnpC–F* homologs are also found in the chromosome, but their expression levels are low.

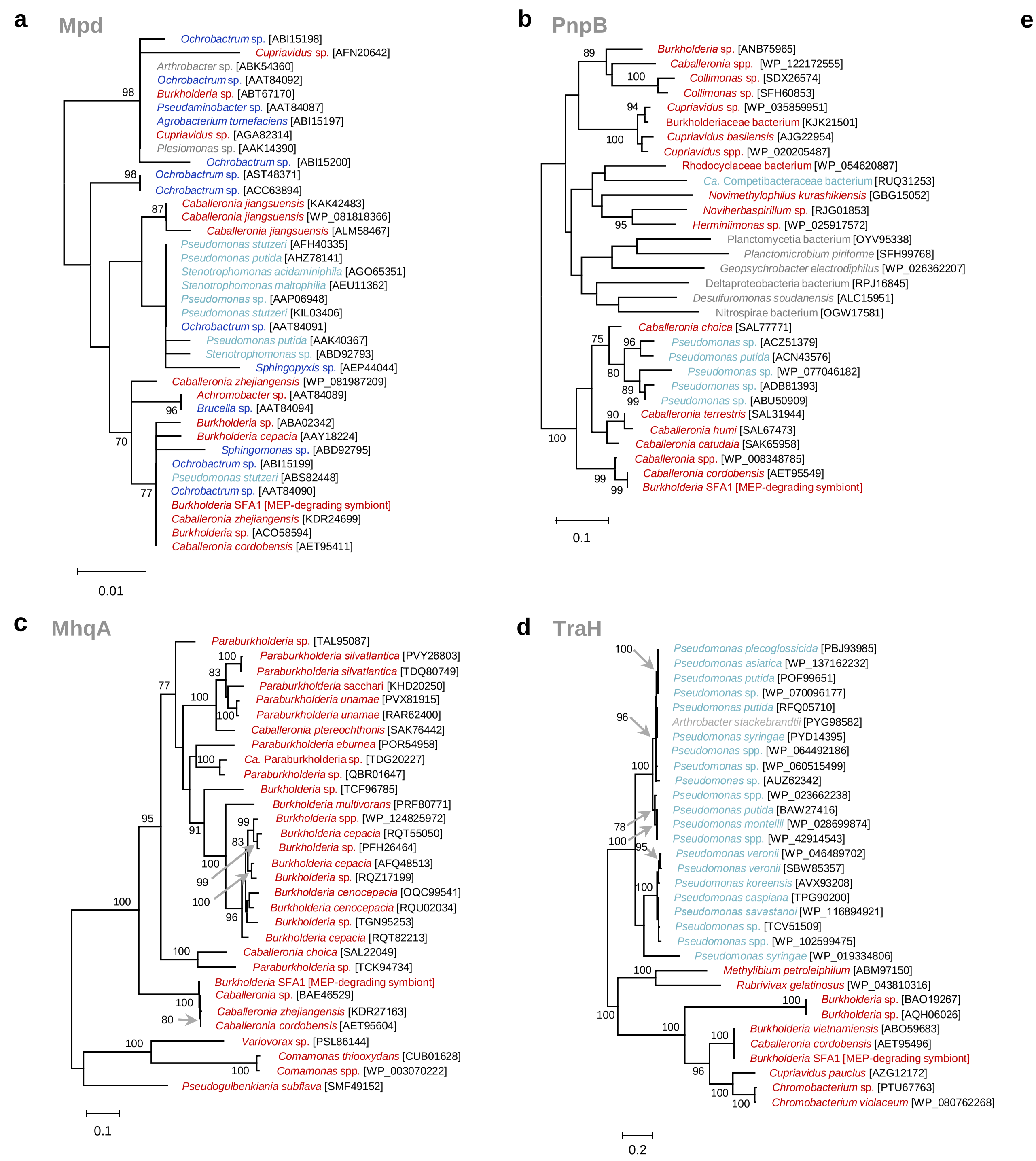

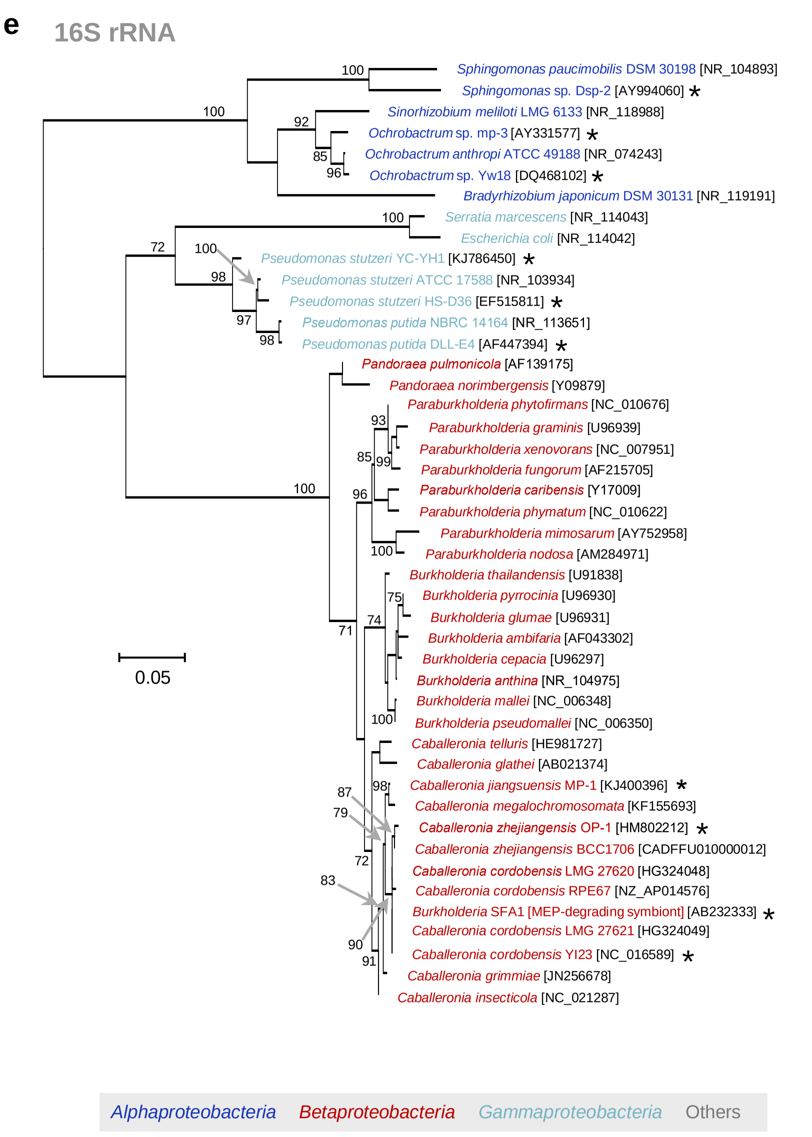

Supplementary Fig. 3 | Phylogenetic trees of MEP-degrading genes. ML trees with bootstrap values (> 70%) are shown. **a,** *mpd*; **b,** *pnpB*; **c,** *mhqA*; **d,** a plasmid transfer gene (*traH*). **e,** Phylogenetic relationship of the MEP-degrading species was estimated by 16S rRNA gene sequences, wherein MEP-degrading species/strains that carry *mpd* are indicated by asterisks. These phylogenetic analyses strongly suggest that the MEP-degrading genes and/or the plasmid itself transmit with a high frequency.

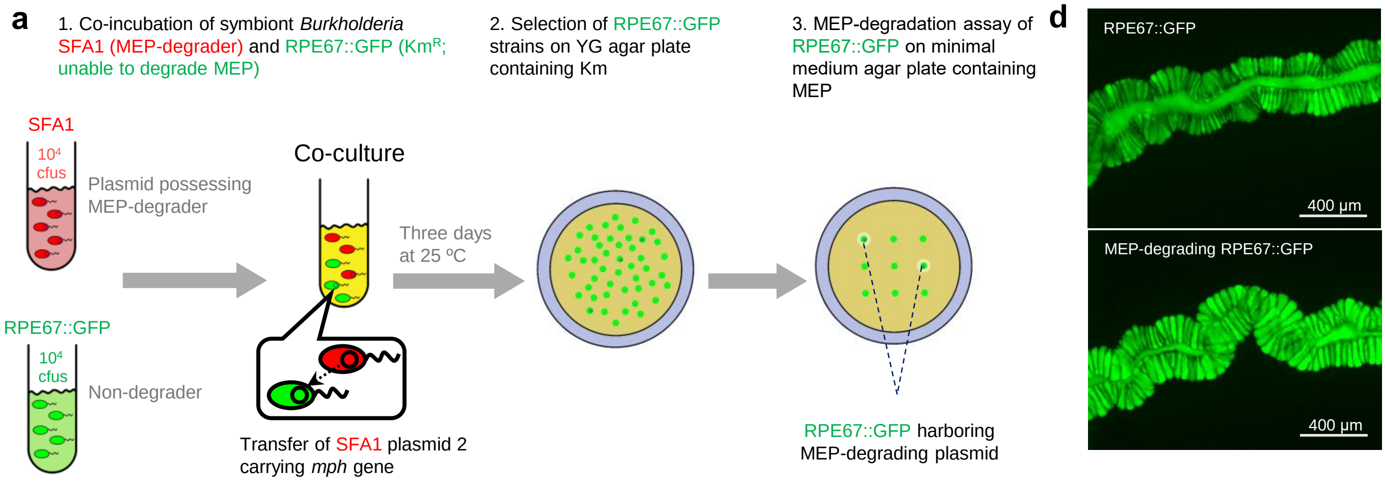

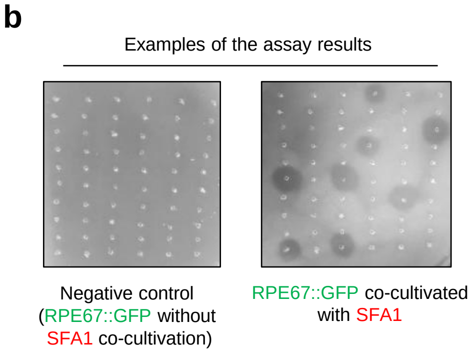

c

Batch	Ratio of MEP-degrading RPE67::GFP (%)[a]
#1	5/96 (5.2%)
#2	6/96 (6.3%)
#3	11/96 (11.5%)
#4	10/96 (10.4%)
#5	9/96 (9.4%)

[a]Ratio of MEP-degrading bacteria was calculated by [The number of MEP-degrading RPE67::GFP colonies / Total number of RPE67::GFP colonies]

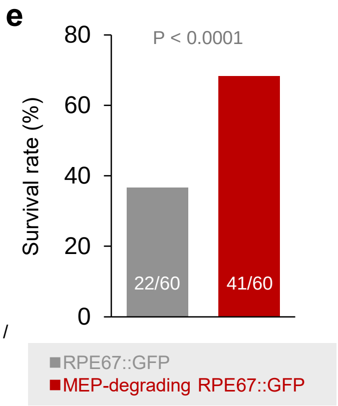

Supplementary Fig. 4 | Horizontal transmission of the MEP-degrading plasmid between *Burkholderia* symbiont strains. **a,** Analysis of the horizontal transmission of the MEP-degrading ability between *Burkholderia* symbiont strains under laboratory condition. SFA1 was co-cultured with RPE67, a non-degrading strain whose chromosome was tagged by GFP, and after overnight incubation, the inter-strain transmission of the MEP-degrading ability from SFA1 to the GFP-labelled RPE67 was investigated by halo-formation on agar plates containing emulsified MEP. **b,** Examples of the assay showing transmission of MEP-degrading ability. **c,** Table of the transmission rate of MEP-degrading ability in five trials. **d,** A newly-emerged MEP-degrading RPE67 derivative colonizes the midgut crypts. Fluorescent microscopy was independently repeated 10 times. **e,** The survival rates after MEP treatment in insects infected with either the non-degrading symbiont (RPE67) or the newly-emerged MEP-degrading symbiont (MEP-degrading RPE67). The method is the same as the one in Figure 2e. Statistically analyzed by two-sided Fisher's exact test.

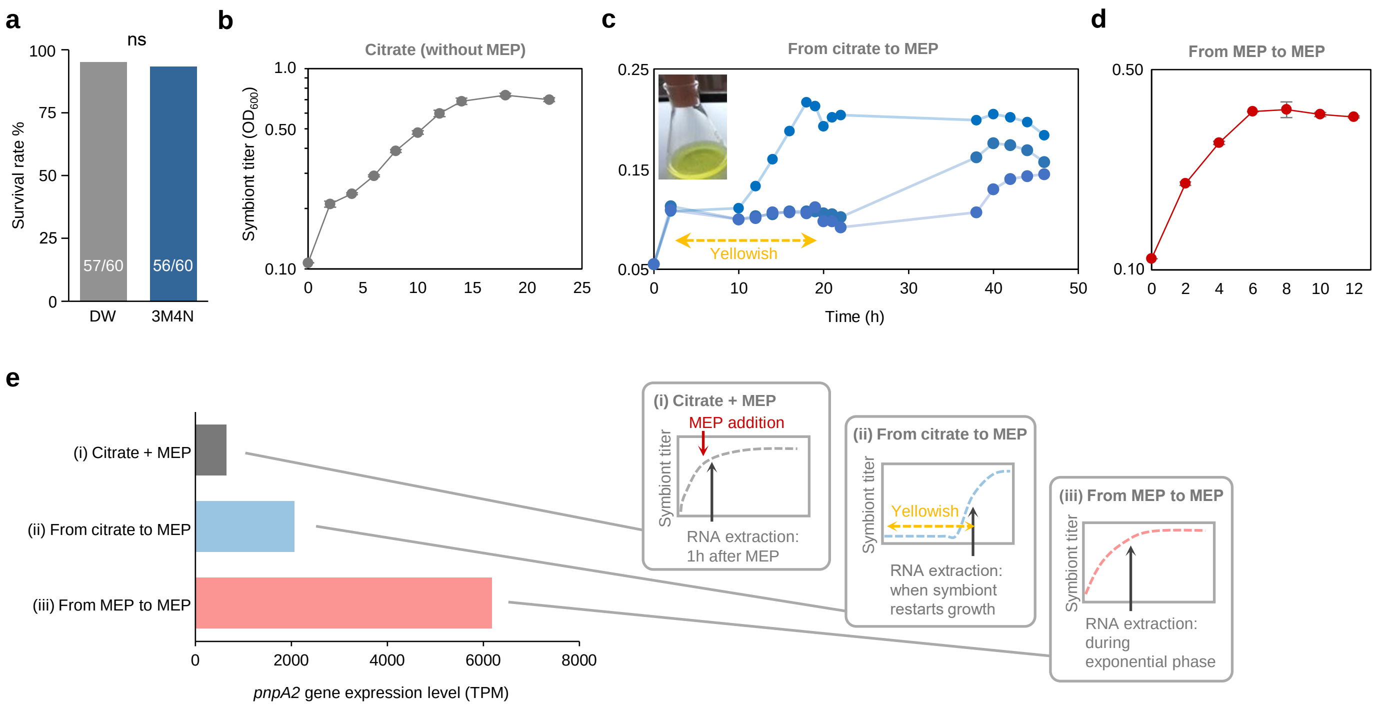

Supplementary Fig. 5 | Effects of 3M4N on the bean bug and the symbiotic bacteria. a, Survival rate of bean bugs that are fed on 3M4N. The survival rates were not significantly different between DWA-fed and 3M4N-fed insects (two-sided Fisher's exact test), indicating 3M4N is non-toxic to the insect. Insects were reared on soybean seeds and either of DWA or 3M4N solution (DWA containing 5 mM 3M4N) for 2 days, and their survival was monitored for 5 days. **b** to **d,** Effects of 3M4N on the symbiotic bacteria: growth delay of SFA1 in MEP medium after pre-culture in citrate medium. **b,** Growth of SFA1 in minimal medium containing 0.1% citrate (citrate medium). Mean ± SD of three replicates are indicated. **c,** Growth of SFA1 in minimal medium containing 1.0 mM of MEP (MEP medium) after pre-culture in the citrate medium. The medium became yellowish because of 3M4N accumulation (see inset picture) and the growth was delayed. After 10-20 h delay, bacterial growth was recovered because of the *pnpA2* expression was induced by 3M4N. The growth curves of three independent cultures are shown. **d,** Growth of SFA1 in MEP medium after pre-culture in MEP medium. No growth delay was observed. Mean ± SD of three replicates are indicated. **e,** Gene expression of *pnpA2* during cultivation with MEP investigated by RNA-seq. Culture conditions: (i) SFA1 was cultured in minimal medium containing citrate and analyzed one hour after MEP addition; (ii) cells were transferred from the citrate medium to MEP medium, wherein growth delay was observed, and analyzed after yellow color of MEP medium started to disappear; (iii) cells were transferred from MEP medium to newly-prepared MEP medium, wherein no growth delay was observed. The values are mean from two replicates.

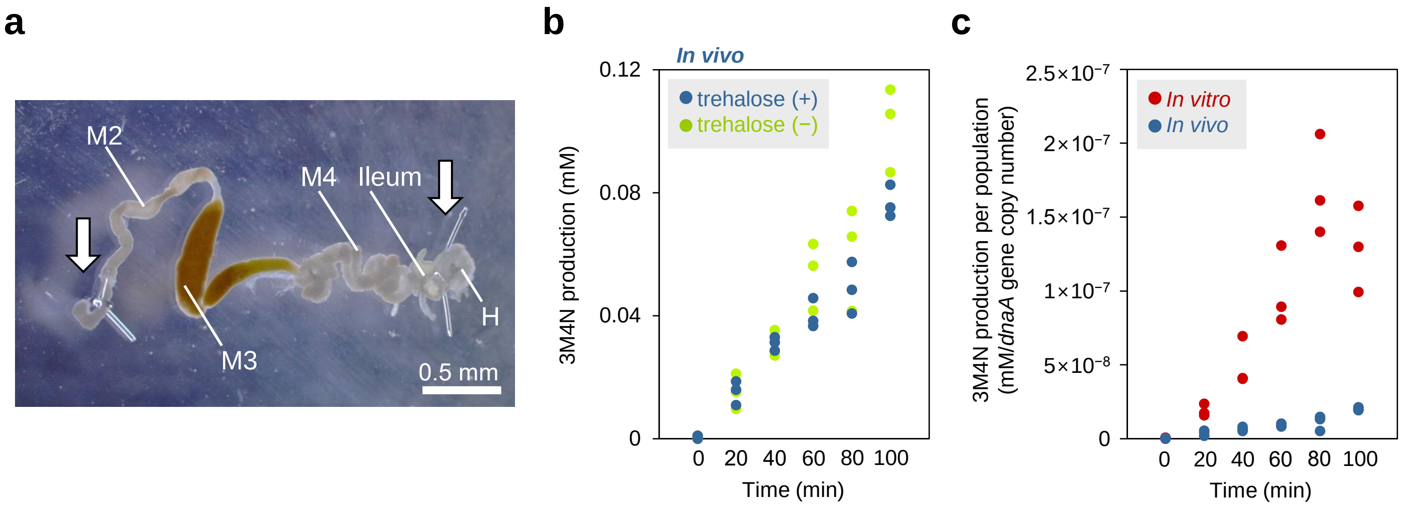

Supplementary Fig. 6 | Detoxification of MEP and production of 3M4N by dissected midguts. a, A dissected gut of a 5th instar insect infected with SFA1, closed at both ends with a 0.2 mm polyethylene fishline knot (arrows). M2, midgut second section; M3, midgut third section; M4, midgut fourth section (symbiotic organ); H, hindgut. **b,** 3M4N production in the gut harboring the MEP-degrading symbiont SFA1 when tissues were incubated in MEP-solution containing 250 mM trehalose. 3M4N production was not significantly different between with and without trehalose for each time point (two-sided Mann–Whitney *U* test after Bonferroni correction). The *in vivo* incubation data without trehalose is from Fig. 3c. **c,** MEP-degrading (3M4N production) rate per single SFA1 cell. The degradation rates shown in Fig. 3c were divided by symbiont cell numbers estimated by qPCR of the *dnaA* gene. The 3M4N-production data represent the results of three independent experiments.

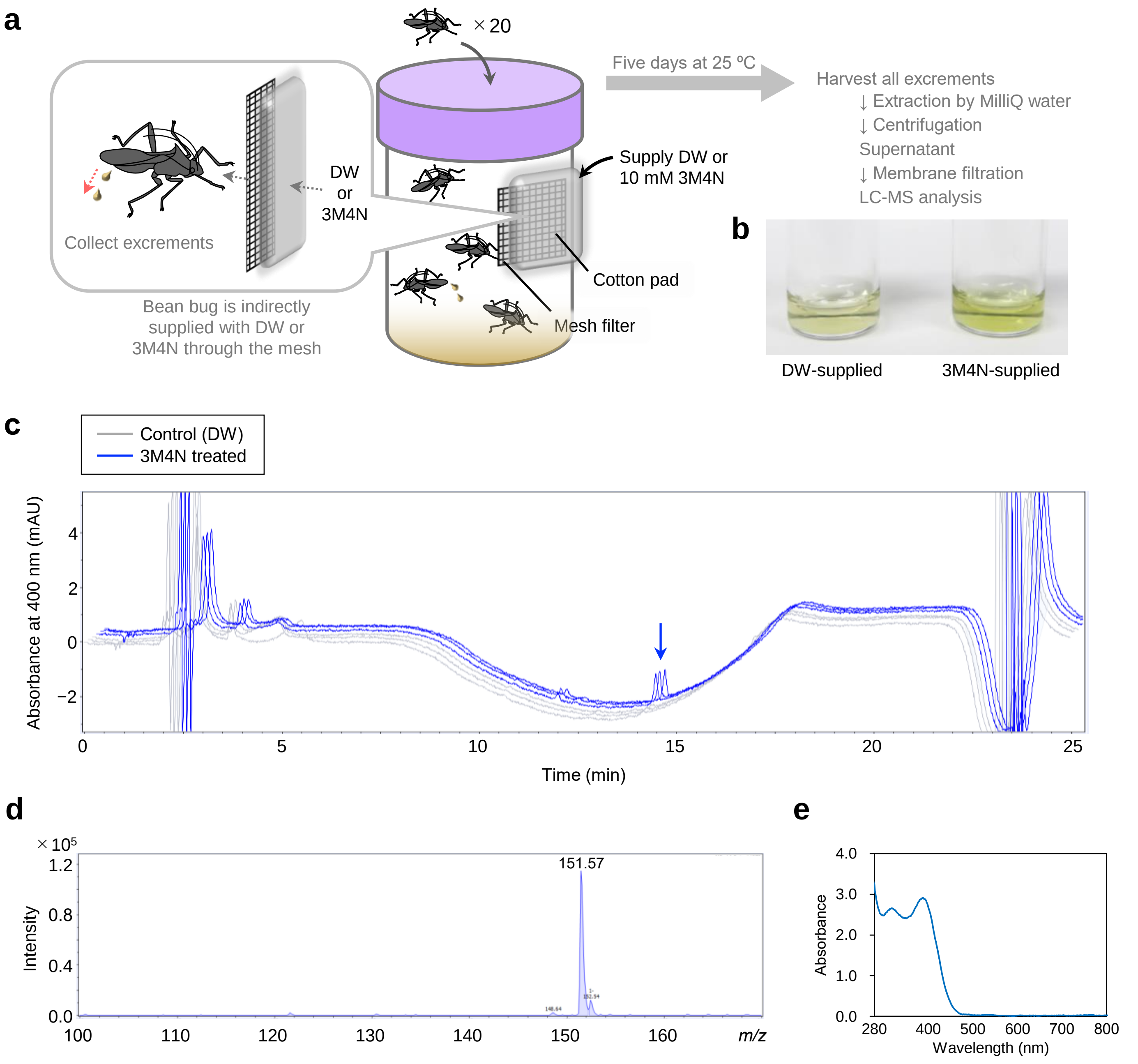

Supplementary Fig. 7 | Detection of 3M4N from feces. a, An insect rearing system for feeding 3M4N and collecting feces. Insects were fed with DW or DW containing 10 mM 3M4N in a plastic container, where the solution supplier was covered by 0.5 mm mesh so that insects were able to drink the solution by probing with their proboscis but did not directly touch the solution by their legs or body. **b,** Color of isolated feces. Feces isolated from the 3M4N-fed group are more yellowish in color. Feces were dissolved in DW adjusted to pH 8.0. **c,** Chromatogram of the feces extracts analyzed by HPLC. Monitoring of absorbance at 400 nm, which corresponds to 3M4N specific absorbance, detected a clear peak at 14.2 min only in the 3M4N-fed group. Triplicate chromatograms of the DW- and 3M4N-fed groups are stacked. **d,** Mass spectrum of the HPLC 14.2 min fraction in negative mode. The *m/z* of 151.57 is identical to that of 3M4N standard (*m/z* = 151.53). The mass and other spectral data of 3M4N standard are available from Spectral Database for Organic Compounds (https://sdbs.db.aist.go.jp/sdbs/cgi-bin/direct_frame_top.cgi). **e,** UV-Vis absorbance spectrum of 1 mM 3M4N. A large peak was observed near 390 nm.

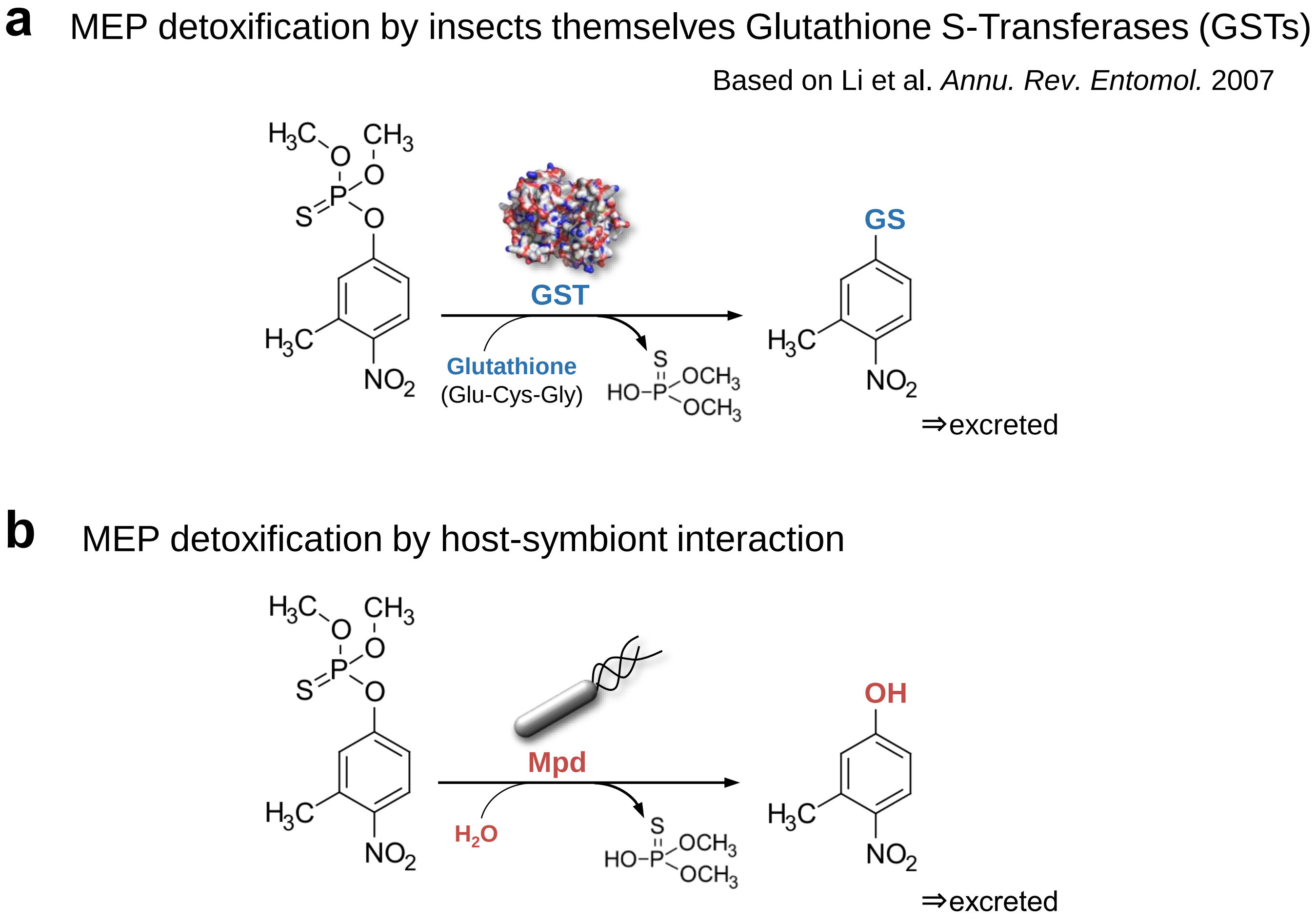

Supplementary Fig. 8 | The analogy between the insect-encoded and the symbiont-mediated detoxification systems. The symbiont-mediated MEP-detoxification mechanism revealed here is analogous to the detoxification mechanism reported in MEP-resistant insects where glutathione S-transferases (GSTs) are highly up-regulated[26]. GSTs detoxify MEP by conjugating glutathione and the symbiont Mpd hydrolyzes MEP. The products of these enzymes, 5-*S*-glutathionyl-1-methyl-2-nitrobenzene and 3M4N, respectively, are immediately eliminated from insect cells and tissues.

Supplementary Table 1 | Primers used in this study

Primer	Sequence[a] (5’ → 3’)	Restriction enzyme	Usage
mph-LF-EcoRI	CTGGAATTCCAGGTAGGCCAGCACCACAC	EcoRI	*mph* deletion
mph-LR	*GGCACGAAACCCAGCAAGCG*GTTCTTCAGG		
mph-RF	*CGCTTGCTGGGTTTCGTGCC*GGTGAACTAC		
mph-RR-HindIII	TCGAAGCTTTGCCCTACATCTCCAAGGTC	HindIII	
pnpA1-LF-EcoI	TTGGAATTCAACTGCTGATGCAGATACGG	EcoRI	*pnpA1* deletion
pnpA1-LR-XbaI	*GATTGTGTCTAGAGTCTCCA*TCTTGTGTCTC	XbaI	
pnpA1-RF-XbaI	*TGGAGACTCTAGACACAATC*GCTCAATTCTC	XbaI	
pnpA1-RR-HindIII	AATAAGCTTGTCCGACTGCGAAACGTC	HindIII	
pnpA2-LF-BamHI	TGAGGATCCCACGGCGAATCAGCTTGTAG	BamHI	*pnpA2* deletion and *pnpA1*/*pnpA2* double deletion
pnpA2-LR-XbaI	*ATCAGCATCTAGAGTTTCCA*TTTTGCAGTC	XbaI	
pnpA2-RF-XbaI	*TGGAAACTCTAGATGCTGAT*GCAGATACGG	XbaI	
pnpA2-RR-HindIII	GTGAAGCTTACTTGAACGCGACATTGCTG	HindIII	
mph-ex-LF-EcoRI	GCCGAATTCCTGCAGCTGCTGCGC	EcoRI	*mph* complementation
mph-ex-LR-BamHI	AGCGGATCCTCAGGGGCATGGTG	BamHI	
mph-ex-RF-HindIII	AAGAAGCTTCGTTTCGTGCCGGTG	HindIII	
mph-ex-RR-HindIII	GCAAAGCTTGGCGGCGCTCAAGGC	HindIII	
mph-ex-F-BamHI	AGGGGATCCAGGATGTAGGAC	BamHI	
mph-ex-R-fsn-kmr	*ATAAAACCGCCTAACGGTGC*GGGCAAG		
kmr-F-fsn-mph	*GCACCGTTAGGCGGTTTTAT*GGACAGCAAG		
kmr-R-BamHI	CTAGGGATCCCCAGAGTCC	BamHI	
tcr-F-XbaI	ATCTCTAGAGAACTCTCTCCCAAAG	XbaI	Tc resistance cassette
tcr-R-XbaI	ACGGTCTAGATTGTTGTATAAGTG	XbaI	
kmr-F-XbaI	GGTTCTAGAGACAGCAAGCGAAC	XbaI	Km resistance cassette
kmr-R-XbaI	AACTCTAGAGTCCCGCTCAGAAG	XbaI	

[a]In the sequences, restriction sites are indicated by underlines, and corresponding restriction enzymes are described right column. Italic indicates complement sequence for the pair primer.
