# Supplementary material for: Insecticide resistance by a host-symbiont reciprocal detoxification
Source: Nat Commun. 2021 Nov 5;12:6432. doi: 10.1038/s41467-021-26649-2 (PMC8571283; doi:10.1038/s41467-021-26649-2)
Supplement: Supplementary file 3 — Reporting Summary [file 41467_2021_26649_MOESM3_ESM.pdf]

nature portfolio

Corresponding author(s): Yoshitomo Kikuchi

Last updated by author(s): Oct 6, 2021

Reporting Summary

Nature Portfolio wishes to improve the reproducibility of the work that we publish. This form provides structure for consistency and transparency in reporting. For further information on Nature Portfolio policies, see our Editorial Policies and the Editorial Policy Checklist.

Statistics

For all statistical analyses, confirm that the following items are present in the figure legend, table legend, main text, or Methods section.

n/a	Confirmed	
☐	☒	The exact sample size (*n*) for each experimental group/condition, given as a discrete number and unit of measurement
☐	☒	A statement on whether measurements were taken from distinct samples or whether the same sample was measured repeatedly
☐	☒	The statistical test(s) used AND whether they are one- or two-sided<br>*Only common tests should be described solely by name; describe more complex techniques in the Methods section.*
☒	☐	A description of all covariates tested
☐	☒	A description of any assumptions or corrections, such as tests of normality and adjustment for multiple comparisons
☐	☒	A full description of the statistical parameters including central tendency (e.g. means) or other basic estimates (e.g. regression coefficient) AND variation (e.g. standard deviation) or associated estimates of uncertainty (e.g. confidence intervals)
☐	☒	For null hypothesis testing, the test statistic (e.g. *F*, *t*, *r*) with confidence intervals, effect sizes, degrees of freedom and *P* value noted<br>*Give P values as exact values whenever suitable.*
☒	☐	For Bayesian analysis, information on the choice of priors and Markov chain Monte Carlo settings
☒	☐	For hierarchical and complex designs, identification of the appropriate level for tests and full reporting of outcomes
☒	☐	Estimates of effect sizes (e.g. Cohen's *d*, Pearson's *r*), indicating how they were calculated

Our web collection on statistics for biologists contains articles on many of the points above.

Software and code

Policy information about availability of computer code

Data collection	Genome sequencing: The Illumina short-reads were processed by using Sickle Ver 1.33 (Available at https://github.com/najoshi/sickle) for removing the low-quality and shorter reads. After processing the Nanopore long-reads with Porechop Ver 0.2.3 (Available at https://github.com/rrwick/Porechop) and Filtlong Ver 0.2.0 (Available at https://github.com/rrwick/Filtlong), error correction was performed by using Canu Ver 1.8. These processed short- and long-reads were assembled by using Unicycler Ver 0.4.7.
Data analysis	Genome sequencing: The assembled genome of SFA1 was annotated by DFAST Ver 1.1.0. After the homology searches of the protein sequences by blastp 2.5.0+ against the COG database (PMID: 25428365), circular replicons were visualized with circos v 0.69-8.<br>Phylogenetic analysis: Nucleotide sequences of 16S rRNA gene were aligned by using SINA v1.2.11. Protein sequences were subjected to the blastp search against the nr database (downloaded in Jul. 2019). Multiple sequencing alignments of each gene were constructed with L-INS-I of mafft v7.407. Unrooted maximum likelihood (ML) phylogenetic trees were reconstructed with RAxML v8.2.3. using the GTR + Γ model (for 16S rRNA gene) or the LG + Γ model (for other genes).<br>RNA-seq: Paired-end reads were checked for quality with FastQC version 0.11.9 (http://www.bioinformatics.babraham.ac.uk/projects/fastqc). The remaining sequencing adaptors and the reads with a cutoff Phred score of 15 (leading and tailing sequences, Phred score of >20) and a length of less than 80 bp were removed by the program Trimmomatic v0.30 using Illumina TruSeq3 adapter sequences for adapter clipping. The paired-end reads were mapped on the genome of symbiont bacteria (DDBJ/EMBL/GenBank accession: AP022305–AP022312) using Bowtie2 ver. 2.2.2. BAM files were converted to BED files with the bamtobed program in BEDTools ver. 2.14.3.

For manuscripts utilizing custom algorithms or software that are central to the research but not yet described in published literature, software must be made available to editors and reviewers. We strongly encourage code deposition in a community repository (e.g. GitHub). See the Nature Portfolio guidelines for submitting code & software for further information.

Data

Policy information about availability of data

All manuscripts must include a data availability statement. This statement should provide the following information, where applicable:

- Accession codes, unique identifiers, or web links for publicly available datasets
- A description of any restrictions on data availability
- For clinical datasets or third party data, please ensure that the statement adheres to our policy

All relevant data including qPCR, bacterial growth rate, and survival rate of inoculated insects are available in the Figshare depository (https://doi.org/10.6084/m9.figshare.16748203.v1).
The annotated genome of the Burkholderia symbiont strain SFA1 has been deposited in the DDBJ/EMBL/GenBank nucleotide sequence database under the accession numbers AP022305–AP022312 and the raw sequence data has been deposited in DRA under the accession number DRA009280. The assembled genome was annotated by using by blastp 2.5.0+ against the COG database (PMID: 25428365). The chromosomes and plasmids were assigned according to the genome of Caballeronia (Burkholderia) cordobensis strain YI23 (CP003087–CP003092 in the DDBJ/EMBL/GenBank nucleotide sequence database).
The RNA-seq nucleotide sequences reported in this study were deposited in the DDBJ/GenBank/EBI databases under the accession number DRA010054.

Field-specific reporting

Please select the one below that is the best fit for your research. If you are not sure, read the appropriate sections before making your selection.

☒ Life sciences ☐ Behavioural & social sciences ☐ Ecological, evolutionary & environmental sciences

For a reference copy of the document with all sections, see nature.com/documents/nr-reporting-summary-flat.pdf

Life sciences study design

All studies must disclose on these points even when the disclosure is negative.

Sample size	No statistical methods were used to predetermine sample size. Sample sizes were determined by magnitude and consistency of measurable differences. For the symbiont inoculation test and the survival measurement, to ensure data robustness, over 60 insect individuals were subjected to the analysis. For both the growth profile measurements and transcriptome analyses, Burkholderia symbiont strain SFA1 was cultured in triplicate, except for the experiment in Fig. S5e (two replicates). The replicate numbers of symbiont culturing and the insect numbers used in this study are given in the figure legends and/or depicted on the bars.
Data exclusions	No data was excluded.
Replication	All attempts at replication were successful. The experiments were replicated two times, wherein similar results were obtained for all independent biological replicates. Data was analyzed by at least two different people independently.
Randomization	Insects were randomly assigned to experimental groups at the beginning of symbiont inoculations.
Blinding	RNA-seq analyses were performed independently from gene identification by genome sequencing and without any prior information on insecticide-degrading genes. qPCR and survival rate measurement of insects infected with mutants were performed by persons without detailed knowledge of the insecticide-degrading genes.

Reporting for specific materials, systems and methods

We require information from authors about some types of materials, experimental systems and methods used in many studies. Here, indicate whether each material, system or method listed is relevant to your study. If you are not sure if a list item applies to your research, read the appropriate section before selecting a response.

Materials & experimental systems

n/a	Involved in the study	
☒	☐	Antibodies
☒	☐	Eukaryotic cell lines
☒	☐	Palaeontology and archaeology
☐	☒	Animals and other organisms
☒	☐	Human research participants
☒	☐	Clinical data
☒	☐	Dual use research of concern

Methods

n/a	Involved in the study	
☒	☐	ChIP-seq
☒	☐	Flow cytometry
☒	☐	MRI-based neuroimaging

Animals and other organisms

Policy information about studies involving animals; ARRIVE guidelines recommended for reporting animal research

Laboratory animals	The bean bug Riptortus pedestris was used. The inbred line used in this study has been reared in the laboratory for over 10 years.
Wild animals	This study did not involve wild animals.
Field-collected samples	This study did not involve field collected samples.
Ethics oversight	No ethical approval was required for this study.

Note that full information on the approval of the study protocol must also be provided in the manuscript.
