# Supplementary material for: Insecticide resistance by a host-symbiont reciprocal detoxification
Source: Nat Commun. 2021 Nov 5;12:6432. doi: 10.1038/s41467-021-26649-2 (PMC8571283; doi:10.1038/s41467-021-26649-2)
Supplement: Supplementary file 2 — Peer Review File [file 41467_2021_26649_MOESM2_ESM.pdf]

nature portfolio

Peer Review File

Insecticide resistance by a host-symbiont reciprocal detoxification

Open Access This file is licensed under a Creative Commons Attribution 4.0 International License, which permits use, sharing, adaptation, distribution and reproduction in any medium or format, as long as you give appropriate credit to the original author(s) and the source, provide a link to the Creative Commons license, and indicate if changes were made. In the cases where the authors are anonymous, such as is the case for the reports of anonymous peer reviewers, author attribution should be to 'Anonymous Referee' followed by a clear attribution to the source work. The images or other third party material in this file are included in the article's Creative Commons license, unless indicated otherwise in a credit line to the material. If material is not included in the article's Creative Commons license and your intended use is not permitted by statutory regulation or exceeds the permitted use, you will need to obtain permission directly from the copyright holder. To view a copy of this license, visit http://creativecommons.org/licenses/by/4.0/.

Reviewers' Comments:

Reviewer #1:
Remarks to the Author:
In this manuscript, Sato and colleagues elucidate the molecular basis of insecticide resistance in the Riptortus-Burkholderia symbiosis. Following up on earlier reports of the same group on insecticide-degrading symbionts in the bean bug, they show that the symbionts encode two different pathways for fenitrothion (MEP) degradation, only one of which is highly upregulated in vitro upon exposure to MEP. Surprisingly, in vitro RNAseq revealed that only the first enzyme of the degradation pathway is constitutively highly expressed, whereas the subsequent ones are effectively silent even when the host is fed a MEP-containing diet. Deletion mutants of the first and second step in the MEP degradation pathway reveal that the first step (conversion of MEP to 3M4N) is necessary and sufficient to confer MEP resistance to the host, but both steps are required for in vitro growth of the symbionts when grown on MEP as the sole carbon source. Interestingly, as 3M4N has bactericidal activity on the symbionts, the authors conclude that the insect effectively remove 3M4N from the midgut crypts, and they provide in vivo evidence that this is indeed the case. This manuscript is a tour-de-force on the fascinating interplay between host and symbiont for the detoxification of an insecticide and reveals the molecular basis for the rapid acquisition of a novel ecological trait through symbiosis in an elegant series of experiments. As such, it will make an outstanding contribution to the scientific literature, and I am convinced that it will receive tremendous attention from a broad scientific readership. While I do have a few comments for the authors to consider in a revision, I am very enthusiastic about the quality and novelty of the science presented in this manuscript.

General comment:
1. The authors present convincing data that the symbiont’s mpd expression is necessary and sufficient for conferring insecticide resistance to the host. However, the dynamics of 3M4N clearance still remain mysterious to me. I urge the authors to consider and address the following aspects:
a. The authors claim that pnp is upregulated upon accumulation of 3M4N in vitro, but they do not explicitly show this. If this is the case, it would indeed make sense that pnp and the following steps in the pathway are not upregulated in vivo, if 3M4N is in fact quickly cleared from the midgut crypts by as yet unknown host mechanisms. Given that this is an important aspect, it would be worth testing whether pnp upregulation indeed responds to the presence of 3M4N. This should be easy to do in vitro, i.e. by assessing pnp expression upon exposure to 3M4N via qPCR or RNAseq.
b. I am somewhat puzzled by the fact that the symbionts convert MEP into 3M4N, and then export the latter to then be cleared by the insect, even though they could convert it to non-bactericidal products. Or am I misunderstanding this, and MEP degradation actually occurs via extracellular enzymes (Mpd)? Since localization is crucial here, it would be good to explain in a bit more detail what is known about the localization of MEP-degrading enzymes.
c. The authors nicely show the presence of 3M4N in the bugs’ feces after oral exposure to this compound. I was a bit surprised to not see data on the excretion of 3M4N in MEP-fed bugs, since this would directly demonstrate 3M4N as the end product of MEP degradation. This experiment would be a good support of the presented scenario on host-symbiont interplay to deal with the insecticide. If this could be done quantitatively (i.e. quantifying the amount of ingested MEP by assessing the amount of consumed liquid and then quantifying 3M4N), the authors could even draw conclusions on the efficiency of degradation, but semi-quantitative measurements would also be sufficient to make the point (i.e. quantifying MEP vs. 3M4N in the feces of MEP-fed bugs).
d. What is the point (from the bacterial perspective) of having a degradation pathway with the first step constitutively expressed that converts a non-toxic substrate into a toxic intermediate, and then have a lag time to upregulate the following enzymes? I have a hard time understanding how this could evolve, unless the substrate is a really common carbon source that makes it worthwhile and compensates for the costs of possible self-intoxication. This is a general question on the degrading ability of the bacteria, regardless of their symbiotic association with the host, so maybe I am missing something important here.

Specific comments:
l. 47: Since this paper is not about an insect feeding on plant sap or vertebrate blood, please keep this more general on insect symbiosis.

l. 61: delete “environomental”
l. 64: delete “to”
l. 83-85: While the phylogenies do indicate quite a bit of horizontal transfer, the symbiont genes are usually embedded in a clade of Burkholderia/Caballeronia, so I am not completely convinced that this provides strong evidence for horizontal acquisition of the plasmid by the symbionts. Am I missing anything here? This is a small point, though, since the authors nicely and convincingly demonstrate that the plasmid can be transferred horizontally.
l. 119: Please mention that MEP was the only carbon source in the MEP medium (right?), since the reader otherwise could interpret the lack of growth of the mpd mutant as a bactericidal effect of MEP (I did at first, until I realized this...).
l. 166: Please rephrase, as the effect of trehalose was not explicitly assessed, since the assays were only done in the presence (but not the absence) of trehalose. What you mean to say is that the effect is observed in the presence of trehalose.
l. 206: Consider replacing “Analogous” with “Thus”, and removing “similar”
Figure 2e: How does the mortality of bugs with mpd mutants compare to those with non-degrading Burkholderia symbionts?
Ext. Data Fig. 2: (a) Please change color of the PnpA and PnpB arrows to black. (b) Please explain in the legend that these are mean expression values from 3 replicates (did I get this right from the methods section?).
Ext. Data Fig. 3: Please highlight the placement of the symbiont. It is not stated anywhere that “BSFA1” is the symbiont. Although it can be deduced from the text, please make the reader’s life easier by stating it here. Also, it would be good to add a phylogenomic analysis of the symbiont to show its affiliation to the genus Burkholderia (or Caballeronia?) and to put the displayed phylogenies into perspective.
Ext. Data Fig. 4: Delete “Phylogenetic tree of mpd and”, which I guess was from a previous version of the manuscript. In (e), please explain better what is displayed (I assume survival of R. pedestris on MEP-containing diet?).
Ext. Data Fig. 7: (b) the 3M4N-fed ones looks greener to me, not yellowish. In fact, it simply looks like a more concentrated fecal suspension. (c,d) Please report the absorbance plot and MS spectrum of the standard.

Reviewer #2:
Remarks to the Author:
This study examined the molecular basis for insecticide detoxification by a gut symbiont of the bean bug. In 2012, some of the same researchers published evidence that an environmentally acquired Burkholderia bacterium confered insecticide resistance upon the insect host; this was a very fascinating result (which has been cited >500 times). This paper provides a very significant extension, showing that the symbiont provides a first enzymatic step, that produces a non-insecticidal product (3M4N). Interestingly, the product is bacteriocidal, which would potentially be self-defeating, since killing the bacteria would remove their beneficial effects for the insect. Other experiments show that the insect excretes the 3M4N, removing it from the gut lumen where the bacteria reside.

I read all of this paper with much interest, including the (long) SI. In general, the claims seem very interesting and also quite well supported. There is quite a lot of data here, including the full bacterial genome, which turns out to be very complex, as it is larger than most bacterial genomes, and contains several chromosomes and numerous plasmids. The genome contained candidate loci for the detoxification activity, as characterized in other bacteria. As it turns out, these are indeed the loci responsible. One core finding of the paper was the use of knockout strains lacking the initial Mpd detoxification enzyme and complemented strains, and the role of this locus is confirmed with classic molecular microbiology methods. Similar knockouts and complements were used to confirm a role of another locus (pnpA2) in further transformation of the 3M4N. Interestingly these loci are on the same bacterial plasmid, though in different locations, and are inducible by MEP.

Phylogenetic trees for these genes show frequent movement among bacterial strains and species, and this ability to transfer among genomes is confirmed by experiments in which a plasmid-harboring strains transmits to another strain.

The experiments with the dissected guts were also ingenious. Some of the physiological mechanisms are not really clear—how does the 3M4N exit the gut, and then how is it excreted? However, these are details that are not really needed for the main story.

Altogether, I found this to be a very rich paper with abundant experimental data to support the proposed route of detoxification. It was also easy to read. The figures are very nice. It is too bad so much good work is buried in the SI, but I can see that it would be a very long paper if included in the main text.

Very minor points:
l. 41 "prosperity" is not the right word to use here.

Extended data Fig 4a: the little dotted lines on the illustratio of the MEP degradation assay seem to be shifte to the left, and not pointing to the correct spots on the plate.

Extended data Fig 6: please define M2, M3, M4, H in the legend. Some people might think H is head, but it seems tobe the other end!

Reviewer #3:
Remarks to the Author:
In this study Sato and co-authors study the molecular and physiological basis of insecticide detoxification in an insect by its associated bacterial symbiont. The study builds upon several previous studies by this same group that have shown how this symbiont that is acquired from the soil at every generation is responsible for insecticide resistance in the insect. The authors perform a series of genomic analyses that identify the potential genes responsible for such degradation. The functionality of these genes is verified through gene silencing coupled with biochemistry assays. The authors also demonstrate that while the symbiotic bacteria degrades the insecticide, which is not bactericidal, the product of this degradation is bactericidal so that in return, the host insect immediately excretes it. The authors confirm such excretion by detecting the degradation product in insect faeces. The authors perform these experiments with bacteria both in vitro and in vivo. In vivo experiments are performed by reinoculating the symbiont (and created mutants) in the insect host. By doing so the authors can test symbiont effects on insect fitness. A surprising result is, however, that the gene expression levels found in vitro and in vivo are quite different, which reveals the important role played by the host in regulating the metabolism of the microbe. The authors also demonstrate that insecticide degradation, which is located in a gene inside a plasmid, can be horizontally transmitted among bacteria. This is important as it shows how insecticide resistance can be transmitted among bacteria and potentially among insect hosts. This part of the study is maybe less clear to me as the authors demonstrate such horizontal transmission between bacteria in vitro, but not in vivo. Also, even if horizontal transmission of this trait is demonstrated among bacteria, whether the same occurs in nature among insect hosts is not demonstrated. As far as I am aware this study is one of the few reported examples where an interaction of mutual benefits mediated by an insect toxic compound has been reported. The manuscript is well presented, clear and suits the general audience of Nature Communications.

Enric Frago
CBGP - Centre for Biology and Management of Populations - Montpellier - France
(I sign all my reviews)

We appreciate the constructive and favorable comments made by the reviewers regarding our manuscript entitled **"Insecticide resistance by a host-symbiont reciprocal detoxification"** (NCOMMS-21-21341-T). We have carefully revised the manuscript and addressed in detail each of the comments/suggestions made by the reviewers. The revised parts and sentences have been highlighted in the marked-up manuscript. Below, we reply point-by-point to all comments (copied in blue).

Response to Reviewer #1:

In this manuscript, Sato and colleagues elucidate the molecular basis of insecticide resistance in the Riptortus-Burkholderia symbiosis. Following up on earlier reports of the same group on insecticide-degrading symbionts in the bean bug, they show that the symbionts encode two different pathways for fenitrothion (MEP) degradation, only one of which is highly upregulated in vitro upon exposure to MEP. Surprisingly, in vitro RNAseq revealed that only the first enzyme of the degradation pathway is constitutively highly expressed, whereas the subsequent ones are effectively silent even when the host is fed a MEP-containing diet. Deletion mutants of the first and second step in the MEP degradation pathway reveal that the first step (conversion of MEP to 3M4N) is necessary and sufficient to confer MEP resistance to the host, but both steps are required for in vitro growth of the symbionts when grown on MEP as the sole carbon source. Interestingly, as 3M4N has bactericidal activity on the symbionts, the authors conclude that the insect effectively remove 3M4N from the midgut crypts, and they provide in vivo evidence that this is indeed the case. This manuscript is a tour
-de-force on the fascinating interplay between host and symbiont for the detoxification of an insecticide and reveals the molecular basis for the rapid acquisition of a novel ecological trait through symbiosis in an elegant series of experiments. As such, it will make an outstanding contribution to the scientific literature, and I am convinced that it will receive tremendous attention from a broad scientific readership. While I do have a few comments for the authors to consider in a revision, I am very enthusiastic about the quality and novelty of the science presented in this manuscript.

---We thank the reviewer for the helpful and useful comments, which have significantly improved this manuscript.

General comment:

1. The authors present convincing data that the symbiont's mpd expression is necessary and sufficient for conferring insecticide resistance to the host. However, the dynamics of 3M4N clearance still remain mysterious to me. I urge the authors to consider and address the following

aspects:

a. The authors claim that pnp is upregulated upon accumulation of 3M4N in vitro, but they do not explicitly show this. If this is the case, it would indeed make sense that pnp and the following steps in the pathway are not upregulated in vivo, if 3M4N is in fact quickly cleared from the midgut crypts by as yet unknown host mechanisms. Given that this is an important aspect, it would be worth testing whether pnp upregulation indeed responds to the presence of 3M4N. This should be easy to do in vitro, i.e. by assessing pnp expression upon exposure to 3M4N via qPCR or RNAseq.

---Although previous studies have elucidated the induction of *pnp* expression by 3M4N, we did not observe any clear data in SFA1. During this study, we conducted the preliminary RNA-seq analyses of cultured symbiont cells, which demonstrated the induction of *pnp* by 3M4N accumulation after MEP degradation. In the revised manuscript, RNA-seq data are shown in Supplementary Figure 5e. The culture conditions of the symbionts were as follows:
(i) The cells were cultured in minimal medium containing citrate, and at the log phase, MEP was added. One hour after the addition of MEP, the cells were collected and subjected to RNA-seq.
(ii) Cells were transferred from the citrate medium to the MEP medium, and RNA-seq was conducted after yellow color of the MEP medium started to disappear.
(iii) Cells were transferred from the spent MEP medium to freshly prepared MEP medium.
The RNA-seq data clearly confirmed that *pnp* is not constitutively expressed but is induced by 3M4N. The new data are included in Supplementary Figure 5 (panel **e**) and are quoted in the main text (L148 in the revised manuscript).

b. I am somewhat puzzled by the fact that the symbionts convert MEP into 3M4N, and then export the latter to then be cleared by the insect, even though they could convert it to non-bactericidal products. Or am I misunderstanding this, and MEP degradation actually occurs via extracellular enzymes (Mpd)? Since localization is crucial here, it would be good to explain in a bit more detail what is known about the localization of MEP-degrading enzymes.

---The cellular localization of Mpd has been thoroughly examined in *Pseudomonas* strains, wherein Mpd is localized in the inner membrane and the degradation from MEP to 3M4N occurs in the periplasmic space (Liu et al. 2005, *Biochem Biophys Res Commun*; Zhongli et al. 2001 Appl Environ Microbiol; Yang et al. 2008, *Biotechnol Bioeng*). By heterologous expression of *mpd* in *E. coli*, Yang et al. (*J Agric Food Chem* 2009) confirmed that Mpd is localized and functions in the inner membrane (and is not secreted into the culture supernatant). The localization of Mpd is now mentioned in the main text with reference to these studies (L114 – 115 in the

revised manuscript). Although the localization was shown in the summarizing illustration (Figure 3d), we apologize for not including this important information in the main text. Thus, under *in vivo* conditions, we expect that 3M4N accumulates in the luminal region of the gut symbiotic organ (M4), which is immediately eliminated by the gut epidermal cells of the host insect without damaging symbiont cells.

c. The authors nicely show the presence of 3M4N in the bugs' feces after oral exposure to this compound. I was a bit surprised to not see data on the excretion of 3M4N in MEP-fed bugs, since this would directly demonstrate 3M4N as the end product of MEP degradation. This experiment would be a good support of the presented scenario on host-symbiont interplay to deal with the insecticide. If this could be done quantitatively (i.e. quantifying the amount of ingested MEP by assessing the amount of consumed liquid and then quantifying 3M4N), the authors could even draw conclusions on the efficiency of degradation, but semi-quantitative measurements would also be sufficient to make the point (i.e. quantifying MEP vs. 3M4N in the feces of MEP-fed bugs).

---Thank you for your useful comment. We agree with the reviewer's suggestion. Although we made multiple attempts to detect MEP and 3M4N in the feces of MEP-fed insects, the detection was unsuccessful. Perhaps, the failure occurred due to foreign substances present in the feces and/or the detection limit of the chemical compounds in feces. When the insects were fed a high dose of MEP for easier detection, the insects did not feed well and produced less feces. Moreover, MEP is a photodegradative compound, which can also hamper quantitative detection. Despite these efforts, we concluded that it is difficult to obtain quantitative data in MEP-fed insects, and to experimentally confirm the excretion of 3M4N by the host. We conducted *in vivo* detoxification tests in dissected guts (Figure 3c), evaluated the effect of 3M4N ingestion on symbiont populations in the gut (Figure 3b), and tested the detection of ingested 3M4N in feces (Supplementary figure 7). Since 3M4N was not toxic to the insects, these experiments were successful and we managed to accumulate supportive evidence for the 3M4N efflux by the host insect.

d. What is the point (from the bacterial perspective) of having a degradation pathway with the first step constitutively expressed that converts a non-toxic substrate into a toxic intermediate, and then have a lag time to upregulate the following enzymes? I have a hard time understanding how this could evolve, unless the substrate is a really common carbon source that makes it worthwhile and compensates for the costs of possible self-intoxication. This is a general question on the degrading ability of the bacteria, regardless of their symbiotic association with the host, so

maybe I am missing something important here.

---Although the driver of such unfavorable metabolic system is not well understood, this is a very interesting point. In the soil environment where these bacteria live, the complex aggregated structure of the soil could not allow sufficient accumulation of 3M4N to harm the bacterium itself. Alternatively, it is reasonable to suggest that the production and accumulation of 3M4N could contribute to the inhibition and sterilization of other surrounding bacteria. In turn, it can benefit the eventual growth of the bacterium and lead to the dominance of the bacterium in the soil habitat. Although this is not the focus of the present study, it can be an intriguing topic for investigating microbial evolution in future studies.

Specific comments:

l. 47: Since this paper is not about an insect feeding on plant sap or vertebrate blood, please keep this more general on insect symbiosis.

---Thank you for your useful comment. We agree, and have revised the sentence in the revised manuscript:

"Many insects possess symbiotic bacteria in the bacteriocytes or the gut, wherein symbionts play pivotal metabolic roles such as the provision of essential amino acids, supplementation of vitamins, and digestion of indigestible food materials, such as plant cell walls."

(L47 – 50 in the revised manuscript)

l. 61: delete "environomental"

---The term has been deleted. (L60 in the revised manuscript)

l. 64: delete "to"

---As suggested "to" was deleted. (L63 in the revised manuscript)

l. 83-85: While the phylogenies do indicate quite a bit of horizontal transfer, the symbiont genes are usually embedded in a clade of Burkholderia/Caballeronia, so I am not completely convinced that this provides strong evidence for horizontal acquisition of the plasmid by the symbionts. Am I missing anything here? This is a small point, though, since the authors nicely and convincingly demonstrate that the plasmid can be transferred horizontally.

---In the revised version, a phylogeny of 16S rRNA gene sequences was included to show the relationships between the bacteria carrying the MEP-degrading genes (Supplementary Fig. 3e). This phylogeny also includes a number of alliances that have neither MEP-degradation abilities nor *mpd*, wherein MEP-degraders carrying *mpd* do not form a monophyletic group. This provides a strong argument for the presence of frequent horizontal transmission of the degradation trait. Although it remains unclear why bacteria of the same genus tend to be clustered together, conjugation compatibility between bacterial species may play a pivotal role. To avoid confusion, the sentence has been revised as follows:
"The phylogeny of bacterial *mpd* genes as well as other genes of the Mhq pathway (Fig. 1c, Supplementary Fig. 3a-d) was inconsistent with the phylogeny of the corresponding bacteria in which the genes were present (Supplementary Fig. 3e), strongly suggesting that they are horizontally transmitted among a broad range of gram-negative bacteria, and that the *Burkholderia* symbiont acquired the MEP-degrading ability via the horizontal transmission of this plasmid."
(L86 – 92 in the revised manuscript).

l. 119: Please mention that MEP was the only carbon source in the MEP medium (right?), since the reader otherwise could interpret the lack of growth of the mpd mutant as a bactericidal effect of MEP (I did at first, until I realized this...).

Thank you for this suggestion. The point (MEP is the sole carbon source in the MEP medium) has been mentioned in the main text. (L127 in the revised manuscript).

l. 166: Please rephrase, as the effect of trehalose was not explicitly assessed, since the assays were only done in the presence (but not the absence) of trehalose. What you mean to say is that the effect is observed in the presence of trehalose.

---We apologize for the confusion. Although the result without trehalose is not shown in the supplementary figure, it is shown in Figure 3c. In this revised version, the results for trehalose are shown in the same panel of the figure (Supplementary Figure 6b). Trehalose was added because it acts as an energy source for insect cells and a higher effect on 3M4N efflux was expected. However, there was no statistically significant difference between the treatments. Even though the data appears to be unimportant, we would like to retain it as some readers may be concerned about the effect of trehalose on the in vivo assay.

l. 206: Consider replacing "Analogous" with "Thus", and removing "similar"

---All the words have been revised accordingly. (L215 – 216 in the revised manuscript).

Figure 2e: How does the mortality of bugs with mpd mutants compare to those with non-degrading Burkholderia symbionts?

---The survival rate of insects infected with non-degrading bacteria is described in our previous work (Kikuchi et al 2012 *PNAS*) and is also shown in Supplementary Figure 4e. According to these data, the survival rate of the insects infected with non-degrading bacteria was less than 40%, which is in agreement with the survival rate of insects infected with the mpd-deletion mutant (Figure 2e). The genetic backgrounds of the stinkbugs were almost identical because we used an inbreeding insect strain maintained since over 10 years in my lab. However, the genetic backgrounds of the mpd-deficient strain (SFA1) and the non-degrading strain (e.g., RPE67) were different. Due to this, we did not include these data for non-degrading strains in Figure 2e.

Ext. Data Fig. 2: (a) Please change color of the PnpA and PnpB arrows to black. (b) Please explain in the legend that these are mean expression values from 3 replicates (did I get this right from the methods section?).

Thank you for this suggestion. The figure and legends have been revised accordingly. (Supplementary Figure 2 in the revised manuscript)

Ext. Data Fig. 3: Please highlight the placement of the symbiont. It is not stated anywhere that "BSFA1" is the symbiont. Although it can be deduced from the text, please make the reader's life easier by stating it here. Also, it would be good to add a phylogenomic analysis of the symbiont to show its affiliation to the genus Burkholderia (or Caballeronia?) and to put the displayed phylogenies into perspective.

---In the phylogenetic trees, "BSFA1" has been replaced by "*Burkholderia* symbiont strain SFA1" The phylogenetic tree of the 16S rRNA gene was included to show the phylogenetic placement of strain SFA1 (it is placed in the stinkbug-associated *Burkholderia* [*Caballeronia*] clade). (Supplementary Figure 3e in the revised manuscript)

Ext. Data Fig. 4: Delete "Phylogenetic tree of mpd and", which I guess was from a previous version of the manuscript. In (e), please explain better what is displayed (I assume survival of R.

pedestris on MEP-containing diet?).

Thank you for pointing this out. The legend has been corrected accordingly. (Supplementary Fig. 4 in the revised manuscript)

Ext. Data Fig. 7: (b) the 3M4N-fed ones looks greener to me, not yellowish. In fact, it simply looks like a more concentrated fecal suspension. (c,d) Please report the absorbance plot and MS spectrum of the standard.

---As the feces are a little greenish-to-brownish in color, and, as we put in about the same amount of feces, we believe that the difference in color is due to the 3M4N excreted by the insects. This color difference is clear and easy to understand, thus, we would like to retain this picture in this figure.

---The mass and other spectral data of the 3M4N standard are available online from the Spectral Database for Organic Compounds (https://sdbs.db.aist.go.jp/sdbs/cgi-bin/direct_frame_top.cgi). The link is shown in the legend of Supplementary fig. 7. In addition, the UV-Vis absorbance spectrum of 1 mM 3M4N has been added to Supplementary Fig. 7e.

<u>Response to Reviewer #2</u>:

This study examined the molecular basis for insecticide detoxification by a gut symbiont of the bean bug. In 2012, some of the same researchers published evidence that an environmentally acquired Burkholderia bacterium confered insecticide resistance upon the insect host; this was a very fascinating result (which has been cited >500 times). This paper provides a very significant extension, showing that the symbiont provides a first enzymatic step, that produces a non-insecticidal product (3M4N). Interestingly, the product is bacteriocidal, which would potentially be self-defeating, since killing the bacteria would remove their beneficial effects for the insect. Other experiments show that the insect excretes the 3M4N, removing it from the gut lumen where the bacteria reside.

I read all of this paper with much interest, including the (long) SI. In general, the claims seem very interesting and also quite well supported. There is quite a lot of data here, including the full bacterial genome, which turns out to be very complex, as it is larger than most bacterial genomes, and contains several chromosomes and numerous plasmids. The genome contained candidate loci for the detoxification activity, as characterized in other bacteria. As it turns out, these are indeed the loci responsible. One core finding of the paper was the use of knockout strains lacking the initial Mpd detoxification enzyme and complemented strains, and the role of this locus is

confirmed with classic molecular microbiology methods. Similar knockouts and complements were used to confirm a role of another locus (pnpA2) in further transformation of the 3M4N. Interestingly these loci are on the same bacterial plasmid, though in different locations, and are inducible by MEP.

Phylogenetic trees for these genes show frequent movement among bacterial strains and species, and this ability to transfer among genomes is confirmed by experiments in which a plasmid-harboring strains transmits to another strain.

The experiments with the dissected guts were also ingenious. Some of the physiological mechanisms are not really clear—how does the 3M4N exit the gut, and then how is it excreted? However, these are details that are not really needed for the main story.

Altogether, I found this to be a very rich paper with abundant experimental data to support the proposed route of detoxification. It was also easy to read. The figures are very nice. It is too bad so much good work is buried in the SI, but I can see that it would be a very long paper if included in the main text.

Thank you for your review and evaluation of our manuscript. The question pointed out by the reviewer “how does the 3M4N exit from the gut” is indeed one of the challenges in future studies. We expect that some types of transporters may be involved in 3M4N excretion, which would be elucidated by RNA-seq of the gut tissue in conjunction with RNAi screening.

Very minor points:

l. 41 “prosperity” is not the right word to use here.

---The term has been replaced by “success” (L41 in the revised manuscript)

Extended data Fig 4a: the little dotted lines on the illustration of the MEP degradation assay seem to be shift to the left, and not pointing to the correct spots on the plate.

The figure has been corrected. (Supplementary Figure 4a in the revised manuscript)

Extended data Fig 6: please define M2, M3, M4, H in the legend. Some people might think H is head, but it seems to be the other end!

Thank you for this suggestion. The abbreviations have been included in the figure legend (Supplementary Figure 6 in the revised manuscript).

Response to Reviewer #3:

In this study Sato and co-authors study the molecular and physiological basis of insecticide detoxification in an insect by its associated bacterial symbiont. The study builds upon several previous studies by this same group that have shown how this symbiont that is acquired from the soil at every generation is responsible for insecticide resistance in the insect. The authors perform a series of genomic analyses that identify the potential genes responsible for such degradation. The functionality of these genes is verified through gene silencing coupled with biochemistry assays. The authors also demonstrate that while the symbiotic bacteria degrades the insecticide, which is not bactericidal, the product of this degradation is bactericidal so that in return, the host insect immediately excretes it. The authors confirm such excretion by detecting the degradation product in insect faeces. The authors perform these experiments with bacteria both in vitro and in vivo. In vivo

experiments are performed by reinoculating the symbiont (and created mutants) in the insect host. By doing so the authors can test symbiont effects on insect fitness. A surprising result is, however, that the gene expression levels found in vitro and in vivo are quite different, which reveals the important role played by the host in regulating the metabolism of the microbe. The authors also demonstrate that insecticide degradation, which is located in a gene inside a plasmid, can be horizontally transmitted among bacteria. This is important as it shows how insecticide resistance can be transmitted among bacteria and potentially among insect hosts. This part of the study is maybe less clear to me as the authors demonstrate such horizontal transmission between bacteria in vitro, but not in vivo. Also, even if horizontal transmission of this trait is demonstrated among bacteria, whether the same occurs in nature among insect hosts is not demonstrated. As far as I am aware this study

is one of the few reported examples where an interaction of mutual benefits mediated by an insect toxic compound has been reported. The manuscript is well presented, clear and suits the general audience of Nature Communications.

---Thank you for the fair and impartial evaluation of our manuscript.

Insecticide resistance by a host-symbiont reciprocal detoxification

Yuya Sato[1,7], Seonghan Jang[2,7], Kazutaka Takeshita[3], Hideomi Itoh[4], Hideaki Koike[5], Kanako Tago[6], Masahito Hayatsu[6], Tomoyuki Hori[1], and Yoshitomo Kikuchi[2,4*]

[1]Environmental Management Research Institute, National Institute of Advanced Industrial Science and Technology (AIST), Tsukuba Center, 305-8569 Tsukuba, Japan
[2]Graduate School of Agriculture, Hokkaido University, 060-8589 Sapporo, Japan
[3]Faculty of Bioresource Sciences, Akita Prefectural University, 010-0195 Akita, Japan
[4]Bioproduction Research Institute, AIST, Hokkaido Center, 062-8517 Sapporo, Japan
[5]Bioproduction Research Institute, AIST, Tsukuba Center, 305-8566 Tsukuba, Japan
[6]Institute for Agro-Environmental Sciences, National Agriculture and Food Research Organization (NARO), 305-8604 Tsukuba, Japan
[7]These authors contributed equally: Yuya Sato, Seonghan Jang

*To whom correspondence should be addressed. E-mail: y-kikuchi@aist.go.jp (Y.K.)

Running title: Host-symbiont reciprocal insecticide detoxification

Abstract

Insecticide resistance is one of the most serious problems in contemporary agriculture and public health[1,2]. Although recent studies revealed that insect gut symbionts contribute to resistance[3,4], the symbiont-mediated detoxification process remains unclear. Here we report the *in vivo* detoxification process of an organophosphorus insecticide, fenitrothion, in the bean bug *Riptortus pedestris*. Using transcriptomics and reverse genetics, we reveal that gut symbiotic bacteria degrade this insecticide through a horizontally acquired insecticide-degrading enzyme into the non-insecticidal but bactericidal compound 3-methyl-4-nitrophenol, which is subsequently excreted by the host insect. This integrated "host-symbiont reciprocal detoxification relay" enables the simultaneous maintenance of symbiosis and efficient insecticide degradation. We also find that the symbiont-mediated detoxification process is analogous to the insect genome-encoded fenitrothion detoxification system present in other insects. Our findings highlight the capacity of symbiosis, combined with horizontal gene transfer in the environment, as a powerful strategy for an insect to instantly eliminate a toxic chemical compound, which could play a critical role in the human-pest arms race.

Introduction

Insects live in a world abounding with toxic compounds such as plant toxins and man-made pesticides. To overcome these toxins, herbivorous insects have evolved elaborate mechanisms for their detoxification[1,2,5–7]. Toxin resistance has brought insects to success in the terrestrial ecosystem, while at the same time, insecticide resistance is one of the most serious problems in contemporary agriculture and public health[2,8,9]. Although the resistance mechanisms are often encoded by the insects' own genomes, recent studies revealed that in many insects specific gut microorganisms also contribute to toxin resistance by degrading the chemical compounds[3,4].

Many insects possess symbiotic bacteria in the bacteriocytes or the gut, wherein symbionts play pivotal metabolic roles such as the provision of essential amino acids, supplementation of vitamins, and digestion of indigestible food materials, such as plant cell walls[10–12]. In addition to the nutritional contribution, recent studies have revealed that symbiotic bacteria confer also other functions, including heat tolerance, parasite or pathogen resistance, body coloration, as well as toxin degradation[13,14]. However, how host-symbiont metabolic interactions play a role in these different symbiont-mediated ecological traits is poorly investigated. Here we report the *in vivo* detoxification process of the insecticide fenitrothion by gut symbionts in the bean bug *Riptortus pedestris*, revealing that a reciprocal host-symbiont detoxification of the insecticide and its bactericidal degradation-product is pivotal to maintain the stable association and thus the efficient detoxification.

The bean bug *R. pedestris*, a serious pest of leguminous crops in Eastern Asia[15], acquires a specific bacterial symbiont of the genus *Burkholderia* from the soil every generation and harbors 10^7–10^8 cells of these bacteria in midgut crypts (Fig. 1a)[16,17]. The

Burkholderia symbiont contributes to the recycling of the host's metabolic wastes, which benefits growth and reproduction of the bean bug host[18]. In addition, some symbiont strains are capable to degrade the organophosphorus insecticide fenitrothion (O,O-dimethyl O-[4-nitro-m-tolyl] phosphorothioate, or MEP). Colonization of the midgut crypts with such a strain results in the instant development of MEP resistance in the host insect[19]. MEP, which inhibits acetylcholine esterases in arthropods and exhibits both oral and percutaneous arthropod-specific toxicities, is one of the most popular organophosphorus insecticides used worldwide[20]. Since the degrading symbiont strains can utilize MEP as a carbon source[21,22], spraying of MEP enriches degrading symbionts in soil, leading to an enhanced infection of the bean bug with MEP-degrading symbionts[23]. The symbiont-mediated resistance is effective for oral and cuticle treatments with MEP[19].

Here, using transcriptomics and reverse genetics, we report the *in vivo* detoxification process of the insecticide fenitrothion by gut symbionts in the bean bug, revealing that a reciprocal host-symbiont detoxification of the insecticide and its bactericidal degradation-product is pivotal to maintain the stable association thereby enabling efficient detoxification.

Results and discussion

MEP-degradation pathway in the *Burkholderia* symbiont

To clarify the genetic bases of the symbiont-mediated MEP resistance, we first determined the whole genome sequence of the typical MEP-degrading *Burkholderia* symbiont, strain SFA1[19]. The genome of SFA1 was 9.5 Mb in size, consisting of three circular chromosomes and five plasmids (Supplementary Fig. 1). This symbiont possessed MEP-degrading gene clusters previously reported in *Pseudomonas* and another

Burkholderia species on the 0.48 Mb plasmid 2 (Fig. 1b, Supplementary Fig. 2). The phylogeny of bacterial *mpd* genes as well as other genes of the Mhq pathway (Fig. 1c, Supplementary Fig. 3a-d) was inconsistent with the phylogeny of the corresponding bacteria in which the genes were present (Supplementary Fig. 3e), strongly suggesting that they are horizontally transmitted among a broad range of gram-negative bacteria, and that the *Burkholderia* symbiont acquired the MEP-degrading ability via the horizontal transmission of this plasmid. Co-cultivation tests demonstrated that the plasmid is indeed frequently transferred from the SFA1 donor to non-degrading symbionts (Supplementary Fig. 4a-c), confirming its mobility between bacteria. Furthermore, the newly emerged degrading symbiont colonized the gut of the bean bug and conferred MEP-resistance on the host insect (Supplementary Fig. 4d and e).

The genomic data suggested that MEP can be assimilated by strain SFA1 via two metabolic pathways branching at 2-methylhydroquinone: the *para*-nitrophenol reductase (Pnp) pathway and the methylhydroquinone metabolizing enzyme (Mhq) pathway (Supplementary Fig. 2a). When the symbiont was cultured in a minimal medium containing MEP as the sole carbon source, the Mhq pathway genes, as well as the upstream methyl parathion degrading enzyme (*mpd*) and *pnpAB* genes, were highly expressed (Fig. 1d, Supplementary Fig. 2b and c). This indicated that SFA1 assimilates MEP mainly via the Mhq pathway.

First catabolic step of MEP-degradation is critical and sufficient for the symbiont-mediated insecticide resistance

We then investigated the expression levels of the MEP-degrading genes in the SFA1 bacteria colonizing the midgut crypts of the insect. Unexpectedly, only the first gene, *mpd*,

was highly expressed while all the downstream genes were nearly silent (Fig. 1d, Supplementary Fig. 2b). Moreover, the expression level of the genes was not affected by MEP treatment of the insects. The *mpd* gene was also highly expressed in bacterial culture grown without MEP (Fig. 1d), indicating that it is constitutively expressed in SFA1. Previous studies on *Pseudomonas* strains reported that Mpd is a membrane protein and the first step of the MEP degradation occurs in the periplasmic space[24–27]. While MEP is highly toxic to insects, the degradation-product, 3-methyl-4-nitrophenol (3M4N; a yellow-colored phenolic compound) (Fig. 2a), is non-toxic (Supplementary Fig. 5a). These results strongly suggested that the MEP resistance is conferred exclusively by the expression of *mpd* in the gut and by the Mpd-mediated conversion of MEP to 3M4N.

To confirm this point, the *mpd* and *pnpA* genes were deleted and MEP-exposure tests were performed with insects infected with the mutants. PnpA, degrading 3M4N to 2-methyl-1,4-benzoquinone, is encoded in strain SFA1 by two homologous genes, *pnpA1* and *pnpA2* (Fig. 1b, d, Supplementary Fig. 2), which were both deleted. These gene deletion mutants, as well as wild type SFA1, grew well in a minimal medium containing glucose as the sole carbon source (Fig. 2b). However, the Δ*mpd* and Δ*pnpA2* single mutants, and the Δ*pnpA1*/Δ*pnpA2* double mutant, but not the Δ*pnpA1* single mutant, were not able to grow in a minimal medium containing MEP as the sole carbon source (Fig. 2c), confirming the involvement of *mpd* and *pnpA2* in the MEP degradation by SFA1. All the deletion mutants colonized well the midgut crypts of the bean bug when insects were reared in the absence of MEP (Fig. 2d). However, when the infected insects were treated with MEP, those harboring the Δ*mpd* mutant showed a significant reduction of the survival rate but not those harboring the Δ*pnpA1*, Δ*pnpA2* or Δ*pnpA1*/Δ*pnpA2* mutants (Fig. 2e). A genetically complemented mutant of *mpd*, Δ*mpd*/mpd^{+}, restored the growth

ability in MEP medium (Fig. 2c) and the ability for conferring MEP resistance in the bean bug host (Fig. 2e). Together, these results demonstrated that (1) the MEP degradation process and gene expression pattern in SFA1 is remarkably different between *in vitro* and *in vivo* conditions, (2) only *mpd* is highly expressed inside the insect gut, and (3) *mpd* is necessary and sufficient for MEP resistance in the insect host.

Insecticide-degradation product 3M4N is highly bactericidal

Notably, the MEP medium of Δ*pnpA2* and Δ*pnpA1*/Δ*pnpA2* cultures became yellow in color due to the accumulation of 3M4N (Fig. 2c). When transferred to MEP medium after culturing in nutrient medium containing citrate as a carbon source, the growth of wild type SFA1 is halting by 10 to 20 hours before resuming (Supplementary Fig. 5b-d). Since *pnpA* expression depends on its substrate 3M4N[28,29], this long growth lag is probably due to the accumulation of 3M4N in the cultures, as suggested by their transient yellowish color (Fig. 2a, Supplementary Fig. 5c), until induction of the *pnpA* expression (Supplementary Fig. 5e). Moreover, it could also indicate that this compound is toxic to the symbiotic bacteria. Indeed, when cultured SFA1 cells were incubated either with MEP or 3M4N and subsequently spotted on agar plates, 3M4N but not MEP showed bactericidal activity (Fig. 3a). Our previous study reported that the symbionts in the midgut crypts show a thinner cell wall compared with the cultured bacteria and that they are highly sensitive to various stresses such as detergents, proteases, and cell surface-attacking antimicrobial peptides[18]. The spot test revealed that, while MEP is still nontoxic, 3M4N shows a drastically higher toxicity to SFA1 cells isolated from midgut crypts than to SFA1 grown in culture (Fig. 3a and b).

Bactericidal product 3M4N is efficiently eliminated by the host from the gut symbiotic organ

The imbalanced expression pattern of *mpd* and *pnpA* in the midgut crypts, *i.e.* constitutive *mpd* expression and little *pnpA* expression, is expected *a priori* to cause an accumulation of 3M4N in the midgut, which could lead to a severe effect on the symbiont population. However, the symbiont titer in the insects was not affected by 3M4N feeding (Fig. 3b). Furthermore, even the Δ*pnpA1*/Δ*pnpA2* mutant, which lacked the 3M4N-degrading enzyme and was thus susceptible to the compound (Fig. 2c), was also not affected by 3M4N feeding in the midgut crypts (Fig. 3b), strongly suggesting non-accumulation of 3M4N in the symbiotic organ. When dissected midguts infected with SFA1 were knotted with a nylon wire at the anterior and posterior portions to avoid bacterial leakage (Supplementary Fig. 6a) and incubated in a phosphate buffer containing 2 mM MEP, 3M4N accumulated in the solution (Fig. 3c). This finding indicates that MEP permeates into the crypt lumen and is degraded by the symbiont and that subsequently its degradation product 3M4N is actively excreted from the midgut to the outside (*i.e.* the hemolymph in the living insect), even though the transportation system of 3M4N through crypt epithelia remains unclear. The low expression of the 3M4N-inducible *pnpA2* gene in the midgut (Fig. 1d) supports this conclusion. The exclusion ability of 3M4N by the midgut was not affected by the presence of trehalose that is known as the major sugar in insect hemolymph (Supplementary Fig. 6b). Although MEP degradation was accomplished in midgut crypts (Fig. 3d), the degradation activity per symbiont cell was approximately ten times lower in the midgut cells than that in cells in culture (Supplementary Fig. 6c). This could be because (1) the expression activity of *mpd* or its protein product is low in the gut; (2) the transport systems of MEP and 3M4N in the gut

epithelial cells limit the degradation efficiency; or (3) symbiont cells are compacted in the gut crypts, which reduces the available surface area of the symbiont for uptake and also the degradation efficiency. When feces of bean bugs fed with water containing 3M4N were analyzed by LC-MS, the compound was detected in the feces (Supplementary Fig. 7), suggesting that 3M4N is not further metabolized in the bean bug host but excreted in unchanged form.

Host-symbiont reciprocal detoxification

Taken together, we conclude that the symbiont-mediated insecticide detoxification is accomplished by an elaborate host-symbiont collaboration. Symbiotic bacteria actively degrade the insecticidal (but not bactericidal) MEP, and in return, the host insect immediately excretes its bactericidal (but not insecticidal) degradation product 3M4N (Fig. 3d). Hence, this coordinated detoxification enables the maintenance of the symbiosis, and thereby sustains high degradation activity, even under insecticide stress. Host-symbiont metabolic integration, wherein both the host and the bacterial symbiont contribute to metabolic pathways, is a common feature in many symbiotic interactions of plant-sucking and blood-feeding insects with some biosynthetic steps performed by symbiont enzymes and others by host-encoded enzymes[10–12]. This study demonstrates that, in the detoxification symbiosis, the host-symbiont metabolic integration is also pivotal.

It should be noted that the symbiont-mediated detoxification process of MEP is remarkably analogous to another known MEP-detoxification mechanism in insects that is mediated by an insect-encoded glutathione S transferase (GST) (Supplementary Fig. 8). Insect GSTs detoxify MEP by conjugating glutathione while the symbiont Mpd

hydrolyzes MEP and their respective products, 5-*S*-glutathionyl-1-methyl-2-nitrobenzene and 3M4N, are subsequently eliminated from the insect cells and tissues. Constitutive high expression of GSTs, reported in MEP-resistant insects[30], can thus be mimicked functionally and mechanistically in insects lacking such a GST by a MEP-degrading symbiont that is constitutively expressing *mpd*, without the need of any mutation in the insects' own genomes to evolve a MEP-modifying GST. Another notable point is that gut symbionts can rapidly gain the detoxification ability through horizontal gene transfer. Diverse bacteria encode insecticide-degrading genes on plasmids[3], and in *Burkholderia* species, MEP-degrading plasmids can be acquired or lost dynamically, depending on the presence of the insecticide in the environment[21] (Supplementary Fig. 4). Thus, highly flexible symbiont-mediated detoxification mechanisms may play a critical role in the evolutionary arms race and integrated co-evolution of toxin-mediated relationships such as plant-herbivore and human-pest antagonisms.

Methods

Insects and bacteria

Bean bugs were reared in petri dishes (90 mm in diameter and 20 mm high) at 25°C under a long-day regimen (16 h light, 8 h dark) and fed with soybean seeds and distilled water containing 0.05% ascorbic acid (DWA). *Burkholderia* symbiont strain SFA1[19], a MEP-degrading strain conferring MEP resistant in the bean bug, and its GFP (green fluorescent protein) labeled derivative, strain SJ586, were used in this study. The symbiont was cultured at 30°C on YG medium (0.5% yeast extract, 0.4% glucose, 0.1% NaCl). The GFP-labelled strain was constructed by the Tn7 mini-transposon system, as previously described[31].

Genome sequencing

DNA was extracted from cultured cells of strain SFA1 by the phenol-chloroform extraction as previously described[32]. The DNA library for Illumina short–reads (the mean insert size: 500 bp) was constructed by using the Covaris S2 ultrasonicator (Covaris) and the KAPA HyperPrep Kit (Kapa Biosystems). For the library construction for Nanopore long-reads, Native Barcoding Expansion (EXP-NBD104; Oxford Nanopore Technologies) and the Ligation Sequencing Kit (SQK-LSK109; Oxford Nanopore Technologies) were used. The genome sequencing was performed with NextSeq using the 2x151 bp protocol (Illumina) and GridION using an R9.4.1 flow cell (Oxford Nanopore Technologies). The Illumina short-reads were processed by using Sickle Ver 1.33 (Available at https://github.com/najoshi/sickle) for removing the low-quality and shorter reads. After processing the Nanopore long-reads with Porechop Ver 0.2.3 (Available at https://github.com/rrwick/Porechop) and Filtlong Ver 0.2.0 (Available at https://github.com/rrwick/Filtlong), error correction was performed by using Canu Ver 1.8[33]. These processed short- and long-reads were assembled by using Unicycler Ver 0.4.7[34], resulting in the eight circular replicons (Supplementary Fig. 1). The assembled genome was annotated by DFAST Ver 1.1.0[35]. After the homology searches of the protein sequences by blastp 2.5.0+[36] against the COG database (PMID: 25428365), circular replicons were visualized with circos v 0.69-8[37]. The chromosomes and plasmids were assigned according to the genome of *Caballeronia* (*Burkholderia*) *cordobensis* strain YI23[38].

Phylogenetic analysis

Nucleotide sequences of 16S rRNA gene of representative *Burkholderia* spp. and outgroup species were aligned by using SINA v1.2.11[39]. Protein sequences of MEP-degrading genes (*mpd*, *pnpB*, and *mhqA*) and a plasmid transfer gene (*traH*) on plasmid 2 were subjected to the blastp search against the *nr* database (downloaded in Jul. 2019) and top ~30 hit sequences were retrieved for each gene. Multiple sequencing alignments of each gene were constructed with L-INS-I of mafft v7.407[40]. Gap-including and ambiguous sites in the alignments were then removed. Unrooted maximum likelihood (ML) phylogenetic trees were reconstructed with RAxML v8.2.3[41] using the GTR + Γ model (for 16S rRNA gene) or the LG + Γ model[42] (for other genes). The bootstrap values of 1,000 replicates for all internal branches were calculated with a rapid bootstrapping algorithm[43].

Preparation of SFA1 cultures for RNA-seq

Burkholderia symbiont SFA1 was pre-cultured in minimal medium (20 mM Phosphate buffer [pH 7.0], 0.01% Yeast, 0.1% $(NH_4)_2SO_4$, 0.02% NaCl, 0.01% $MgSO_4{\cdot}7H_2O$, 0.005% $CaCl_2{\cdot}2H_2O$, 0.00025% $FeSO_4{\cdot}7H_2O$, 0.00033% EDTA·2Na) containing 1.0 mM of MEP on a gyratory shaker (210 rpm) at 30ºC overnight, and sub-cultured in newly-prepared MEP-containing minimal medium under the same conditions for 5 h. As a control, SFA1 was pre-cultured in minimal medium containing 0.1% citrate overnight, and then the overnighter was sub-cultured in a newly-prepared citrate-containing minimal medium under the same conditions for 10 h. The culture was mixed with an equal amount of RNAprotect Bacteria Regent (Qiagen, Valencia, CA, USA), then centrifuged to harvest the cells for the RNA-seq analysis.

Preparation of midgut symbiont cells for RNA-seq

The oral administration of the symbiont strain SFA1 was performed as described[19,44]. The symbiont was inoculated to 2nd instar nymphs, and three days after molting to the 3rd instar, nymphs were transdermally administrated with 1 µl of 0.2 µM or 20 µM of MEP (dissolved in acetone). One- or three-day after the treatment, insects were dissected and the crypt-bearing symbiotic gut region was subjected to the RNA extraction and RNA-seq analysis. As a control, untreated insects were analyzed.

RNA-seq analysis

Total RNA was extracted from triplicate samples from cultures by the hot phenol method as previously described[45] or from the midgut symbiont cells by using RNAiso Plus (Takara Bi, Kusatsu, Shiga, Japan) and the RNeasy mini kit (Qiagen). The extracted total RNA was purified by phenol–chloroform extraction and digestion by DNase (RQ1 RNase-Free DNase, Promega, Fitchburg, WI, USA) and re-purified by using a RNeasy Mini Kit. The mRNA in the samples were further enriched by the RiboMinus Transcriptome Isolation Kit, bacteria (Thermo Fisher Scientific, Waltham, MA, USA) and the RiboMinus Eukaryote Kit for RNA-Seq (Thermo Fisher Scientific) and purified by using an AMPure XP kit (Beckman Coulter, Brea, CA, USA). The cDNA libraries were constructed from approximately 100 ng of rRNA-depleted RNA samples by the use of a NextUltraRNA library prep kit (New England Biolabs, Ipswich, MA, USA). Size selection of cDNA (200–300 bp) and determination of the size distribution and concentration of the purified cDNA samples were performed as described previously[46]. In total, 21 cDNA libraries were constructed and sequenced by MiSeq (Illumina, Inc., San Diego, CA, USA). To ensure high sequence quality, the remaining sequencing

adaptors and the reads with a cutoff Phred score of 15 (for leading and tailing sequences, Phred score of >20) and a length of less than 80 bp in the obtained RNA-seq data were removed by the program Trimmomatic v0.30 using Illumina TruSeq3 adapter sequences for the clipping[47]. The remaining paired reads were analyzed by FastQC (http://www.bioinformatics.babraham.ac.uk/projects/fastqc/) for quality control, and Bowtie2 ver. 2.2.2[48] for mapping on the symbiont genome (DDBJ/EMBL/GenBank accession: AP022305–AP022312). After the conversion of the output BAM files to BED files using the bamtobed program in BEDTools ver. 2.14.3[49], gene expression levels were calculated in TPM (Transcripts Per kilobase Million) values by using in-house scripts[46].

Gene deletion and complementation

MEP-degrading genes (*mpd*, *pnpA1*, and *pnpA2*) were deleted by the homologous-recombination based deletion method using pK18mobsacB or pUC18, as previously described[50,51]. Primers used for the mutagenesis are listed in Supplementary Table 1. For *mpd* gene deletion, pK18mobsacB was used to construct a markerless mutant. For single deletion of *pnpA1* and *pnpA2* genes, pUC18 was used to substitute each gene locus with a kanamycin resistance gene cassette. The double deletion of *pnpA1* and *pnpA2* genes was performed by substituting *pnpA2* gene locus with a tetracycline resistance gene cassette in the *pnpA1* deletion mutant. Gene complementation of *mpd* was also performed by homologous recombination using plasmid pUC18 with primers listed in Supplementary Table 1. To investigate growth profiles of the wild type SFA1, the gene-deletion mutants (Δ*mpd*, Δ*pnpA1*, Δ*pnpA2*, and Δ*pnpA1*/Δ*pnpA2*), and the *mph*-complement mutant (Δ*mpd*/*mpd*$^+$) in the MEP-containing minimal medium, the strains were pre-cultured in minimal medium containing 1.0 mM MEP on a gyratory shaker (210

rpm) at 30ºC overnight, and then cultured in newly-prepared MEP-containing minimal medium under the same condition. The growth of cultures was estimated by OD_{600} measurements. To confirm the basic growth abilities of the mutants, these bacterial strains were pre- and sub-cultured in minimal medium containing 0.1% glucose under the same conditions. These symbiont strains and mutants were inoculated to the bean bug as described above.

Quantitative PCR

Symbiont titers in the midgut crypts were evaluated by quantitative PCR (qPCR) of bacterial *dnaA* gene copies. The qPCR was performed by using a KAPA SYBR Fast qPCR Master Mix (Kapa Biosystems) and the LightCycler 96 System (Roche Applied Science) with the following primers: BSdnaA-F (5′-AGC GCG AGA TCA GAC GGT CGT CGA T-3′) and BSdnaA-R (5′-TCC GGC AAG TCG CGC ACG CA-3′).

MEP treatment of insects

MEP treatment of *R. pedestris* was performed as previously described[19]. Soybean seeds were dipped in 0.2 mM MEP for 5 sec and dried at room temperature. In each clean plastic container, 15 individuals of 3rd instar nymphs were reared on three seeds of the MEP-treated soybean and DWA at 25°C under the long-day regime, and the number of dead insects were counted 24 h after the treatments. The survival rate of the insects was analyzed under Fisher's exact test by use of the program R ver. 3.6.3 (Available at https://www.R-project.org/). Multiple comparisons were corrected by the Bonferroni method.

Bactericidal activities of MEP and its degradation-product 3M4N

To measure bactericidal activities of MEP and 3M4N on cultured cells of SFA1, 10^4 cells of log-phase growing bacteria were mixed with a defined concentration of MEP or 3M4N, and spotted on a YG agar plate. To measure the bactericidal activity against midgut crypt-colonizing cells, the symbiotic organs infected with SFA1 were dissected from 3rd instar insects, homogenized in PBS, and purified by a 5 µm size pore Syringe filter to harvest colonizing symbiont cells[50]. MEP or 3M4N was added to approximately 10^4 cells of the harvested cells and spotted on a YG agar plate. Bactericidal activities of the chemical compounds were then checked in 24 h after incubation at 30°C.

HPLC detection of *in vitro* and *in vivo* MEP-degrading activities of the symbiont

To determine *in vitro* MEP-degradation activity, cultured cells of SFA1 were prepared as above, and 10^6 cells were incubated at 25°C in 200 µl of MEP-solution (2 mM MEP in Tris-Hcl [pH 8.5] with 0.1% Triton X-100) in a 1.5 ml microtube. To determine *in vivo* MEP-degradation activity, the midgut of a 5th instar insect infected with SFA1 was dissected, the posterior and anterior parts of the crypt-bearing symbiotic region were closed with 0.2 mm polyethylene fishline (Supplementary Fig. 6a) and incubated at 25°C in 200 µl of the MEP-solution. For the *in vivo* determination, 250 mM of trehalose, known as a major sugar source of insects' hemolymph[52], was added to the MEP-solution to keep the tissue fresh. After incubation for different times, the reaction was stopped by adding 400 µl of methanol. After centrifugation, supernatants were subjected to high performance liquid chromatography (HPLC) analyses to detect MEP and 3M4N, as previously reported[21], and precipitated cells and tissues were subjected to DNA extraction and qPCR to estimate symbiont cell numbers of each reaction.

LC–ESI–MS detection of 3M4N in feces from 3M4N-fed insects

An insect rearing system for feeding 3M4N and collecting feces is shown in Supplementary Fig. 7. Insects were fed with DW or DW containing 10 mM 3M4N in a plastic container, in which the solution supplier was covered by 0.5 mm mesh so that insects were able to drink the solution by probing with their proboscis but did not directly touch the solution by their legs or body. Twenty insects were reared per container and their feces were accumulated on the bottom of the container for five days. The collected feces (DW- or 3M4N-treated) were suspended in 1 ml of MilliQ water, and the water-soluble fractions were extracted by thorough vortexing. Solids and insoluble fractions were removed from the suspension by centrifugation and subsequent filtration using a cellulose acetate membrane (Φ, 0.20 μm, ADVANTEC, Tokyo, Japan). The resultant fraction was diluted 10-fold by MilliQ water and analyzed by liquid chromatography–electrospray ionization mass spectrometry (LC–ESI–MS) according to a previous report[53]. HPLC was performed using the Nexera X2 system (Shimadzu, Kyoto, Japan) composed of LC-30AD pump, SPD-M30A photodiode array detector and SIL-30AC autosampler. Develosil HB ODS-UG column (ID 2.0 mm x L 75 mm; Nomura Chemical Co., Ltd, Aichi, Japan) was employed with a flow rate of 0.2 mL/min. The following gradient system was used for analysis of metabolites: MilliQ water (solvent A) and methanol (solvent B), 90% A and 10% B at 0–5 min, linear gradient from 90% A and 10% B to 20% A and 80% B at 5–15 min, 20% A and 80% B at 15–20 min, 90% A and 10% B at 20–25 min. Retention time of 3M4N standard reagent was 14.2 min. Electrospray ionizationmass spectrometry (ESI–MS) in positive and negative ion modes were

simultaneously performed using amaZon SL (Bruker, Billerica, MA, USA). 3M4N (MW=153.14) standard showed a clear peak in negative mode at *m/z* of 151.53.

Data availability

All relevant data are available from the corresponding authors upon request. The annotated genome of the *Burkholderia* symbiont strain SFA1 has been deposited in the DDBJ/EMBL/GenBank nucleotide sequence database under the accession numbers AP022305–AP022312 and the raw sequence data has been deposited in DRA under the accession number DRA009280. The RNA-seq nucleotide sequences reported in this study were deposited in the DDBJ/GenBank/EBI databases under the accession number DRA010054.

References

1. Després, L., David, J.-P. & Gallet, C. The evolutionary ecology of insect resistance to plant chemicals. *Trends Ecol. Evol.* **22**, 298–307 (2007).

2. Heckel, D. G. Insecticide resistance after silent spring. *Science* 337, 1612–1614 (2012).

3. Itoh, H., Tago, K., Hayatsu, M. & Kikuchi, Y. Detoxifying symbiosis: microbe-mediated detoxification of phytotoxins and pesticides in insects. *Nat. Prod. Rep.* **35**, 434–454 (2018).

4. Hammer, T. J. & Bowers, M. D. Gut microbes may facilitate insect herbivory of chemically defended plants. *Oecologia* **179**, 1–14 (2015).

5. Hemingway, J., Field, L. & Vontas, J. An overview of insecticide resistance. *Science* **298**, 96–97 (2002).

6. Alyokhin, A. & Chen, Y. H. Adaptation to toxic hosts as a factor in the evolution of insecticide resistance. *Curr. Opin. Insect Sci.* **21**, 33–38 (2017).

7. Hemingway, J. & Ranson, H. Insecticide Resistance in insect vectors of human disease. *Entomology* **45**, 371–391 (2000).

8. Hemingway, J. *et al.* Averting a malaria disaster: will insecticide resistance derail malaria control? *Lancet* **387**, 1785–1788 (2016).

9. Correy, G. J. *et al.* Overcoming insecticide resistance through computational inhibitor design. *Proc. Natl. Acad. Sci. USA* **116**, 21012–21021 (2019).

10. Consortium, T. I. A. G. Genome Sequence of the Pea Aphid *Acyrthosiphon pisum*. *PLoS Biology* **8**, e1000313 (2010).

11. McCutcheon, J. P. & Moran, N. A. Extreme genome reduction in symbiotic bacteria. *Nat. Rev. Microbiol.* **10**, 13–26 (2012).

12. Bublitz, D. C. *et al.* Peptidoglycan production by an insect-bacterial mosaic. *Cell* **179**, 703-712.e7 (2019).

13. Douglas, A. E. Multiorganismal insects: Diversity and function of resident microorganisms. *Annu. Rev. Entomol.* **60**, 1–18 (2015).

14. McLean, A. H. C., Parker, B. J., Hrček, J., Henry, L. M. & Godfray, H. C. J. Insect symbionts in food webs. *Philos. Trans. R. Soc. B Biol. Sci.* **371**, 20150325 (2016).

15. Schaefer, C. & Panizzi, A. Heteroptera of Economic Importance. (CRC, Boca Raton, FL, 2000).

16. Takeshita, K. & Kikuchi, Y. Riptortus pedestris and Burkholderia symbiont: an ideal model system for insect–microbe symbiotic associations. *Res. Microbiol.* **168**, 175–187 (2017).

17. Kaltenpoth, M. & Flórez, L. V. Versatile and dynamic symbioses between insects and *Burkholderia* bacteria. *Annu. Rev. Entomol.* **65**, 1–26 (2019).

18. Ohbayashi, T. *et al.* Comparative cytology, physiology and transcriptomics of *Burkholderia insecticola* in symbiosis with the bean bug *Riptortus pedestris* and in culture. *ISME J.* **13**, 1469–1483 (2019).

19. Kikuchi, Y. *et al.* Symbiont-mediated insecticide resistance. *Proc. Natl. Acad. Sci. USA* **109**, 8618–8622 (2012).

20. Stenersen, J. Chemical pesticides: Mode of action and toxicology. (CRC, Boca Raton, FL, 2004).

21. Hayatsu, M., Hirano, M. & Tokuda, S. Involvement of two plasmids in fenitrothion degradation by *Burkholderia* sp. strain NF100. *Appl. Environ. Microbiol.* **66**, 1737–1740 (2000).

22. Itoh, H. *et al.* Bacterial population succession and adaptation affected by insecticide application and soil spraying history. *Front. Microbiol.* **5**, 457 (2014).

23. Itoh, H. *et al.* Infection dynamics of insecticide-degrading symbionts from soil to insects in response to insecticide spraying. *ISME J.* **12**, 909–920 (2018).

24. Zhongli, C., Shunpeng, L. & Guoping, F. Isolation of methyl parathion-degrading strain M6 and cloning of the methyl parathion hydrolase gene. *Appl. Environ. Microbiol.* **67**, 4922–4925 (2001).

25. Liu, H., Zhang, J.-J., Wang, S.-J., Zhang, X.-E. & Zhou, N.-Y. Plasmid-borne catabolism of methyl parathion and p-nitrophenol in *Pseudomonas* sp. strain WBC-3. *Biochem. Bioph. Res. Co*. **334**, 1107–1114 (2005).

26. Yang, C. *et al.* Surface display of MPH on *Pseudomonas putida* JS444 using ice nucleation protein and its application in detoxification of organophosphates. *Biotechnol. Bioeng.* **99**, 30–37 (2008).

27. Yang, C., Freudl, R. & Qiao, C. Export of methyl parathion hydrolase to the periplasm by the twin-arginine translocation pathway in *Escherichia coli*. *J. Agr. Food Chem.* **57**, 8901–8905 (2009).

28. Zhang, J.-J., Liu, H., Xiao, Y., Zhang, X.-E. & Zhou, N.-Y. Identification and characterization of catabolic para-nitrophenol 4-monooxygenase and para-

benzoquinone reductase from *Pseudomonas* sp. strain WBC-3. *J. Bacteriol.* **191**, 2703–2710 (2009).

29. Min, J., Zhang, J.-J. & Zhou, N.-Y. The gene cluster for para-nitrophenol catabolism is responsible for 2-chloro-4-nitrophenol degradation in *Burkholderia* sp. strain SJ98. *Appl. Environ. Microbiol.* **80**, 6212–6222 (2014).

30. Li, X., Schuler, M. A. & Berenbaum, M. R. Molecular mechanisms of metabolic resistance to synthetic and natural xenobiotics. *Entomology* **52**, 231–253 (2007).

31. Kikuchi, Y. & Fukatsu, T. Live imaging of symbiosis: spatiotemporal infection dynamics of a GFP-labelled *Burkholderia* symbiont in the bean bug *Riptortus pedestris*. *Mol. Ecol.* **23**, 1445–1456 (2014).

32. Kikuchi, Y., Hosokawa, T. & Fukatsu, T. An ancient but promiscuous host–symbiont association between *Burkholderia* gut symbionts and their heteropteran hosts. *ISME J.* **5**, 446–460 (2011).

33. Koren, S. *et al.* Canu: scalable and accurate long-read assembly via adaptive k-mer weighting and repeat separation. *Genome. Res.* **27**, 722–736 (2017).

34. Wick, R. R., Judd, L. M., Gorrie, C. L. & Holt, K. E. Unicycler: Resolving bacterial genome assemblies from short and long sequencing reads. *PLoS Comput. Biol.* **13**, e1005595 (2017).

35. Tanizawa, Y., Fujisawa, T. & Nakamura, Y. DFAST: a flexible prokaryotic genome annotation pipeline for faster genome publication. *Bioinformatics* **34**, 1037–1039 (2017).

36. Camacho, C. *et al.* BLAST+: architecture and applications. *BMC Bioinformatics* **10**, 421 (2009).

37. Krzywinski, M. *et al.* Circos: An information aesthetic for comparative genomics. *Genome. Res.* **19**, 1639–1645 (2009).

38. Lim, J. S. *et al.* Complete genome sequence of the fenitrothion-degrading *Burkholderia* sp. strain YI23. *J. Bacteriol.* **194**, 896–896 (2012).

39. Pruesse, E., Peplies, J. & Glöckner, F. O. SINA: Accurate high-throughput multiple sequence alignment of ribosomal RNA genes. *Bioinformatics* **28**, 1823–1829 (2012).

40. Katoh, K. & Standley, D. M. MAFFT multiple sequence alignment software Version 7: Improvements in performance and usability. *Mol. Biol. Evol.* **30**, 772–780 (2013).

41. Stamatakis, A. RAxML version 8: a tool for phylogenetic analysis and post-analysis of large phylogenies. *Bioinformatics* **30**, 1312–1313 (2014).

42. Le, S. Q. & Gascuel, O. An improved general amino acid replacement matrix. *Mo.l Biol. Evol.* **25**, 1307–1320 (2008).

43. Stamatakis, A., Hoover, P., Rougemont, J. & Renner, S. A rapid bootstrap algorithm for the RAxML web servers. *Syst. Biol.* **57**, 758–771 (2008).

44. Ishigami, K., Jang, S., Itoh, H. & Kikuchi, Y. Insecticide resistance governed by gut symbiosis in a rice pest, Cletus punctiger, under laboratory conditions. *Biol Lett.* **17**, 20200780 (2021).

45. Sato, Y. *et al.* Transcriptome analyses of metabolic enzymes in thiosulfate- and hydrogen-grown *Hydrogenobacter thermophilus* cells. *Biosci. Biotechnol. Biochem.* **76**, 1677–1681 (2012).

46. Sato, Y. *et al.* Transcriptome analysis of activated sludge microbiomes reveals an unexpected role of minority nitrifiers in carbon metabolism. *Commun. Biol.* **2**, 179 (2019).

47. Bolger, A. M., Lohse, M. & Usadel, B. Trimmomatic: a flexible trimmer for Illumina sequence data. *Bioinformatics* **30**, 2114–2120 (2014).

48. Langmead, B. & Salzberg, S. L. Fast gapped-read alignment with Bowtie 2. *Nat. Methods* **9**, 357–359 (2012).

49. Quinlan, A. R. & Hall, I. M. BEDTools: a flexible suite of utilities for comparing genomic features. *Bioinformatics* **26**, 841–842 (2010).

50. Kim, J. K. *et al.* Polyester synthesis genes associated with stress resistance are involved in an insect–bacterium symbiosis. *Proc. Natl. Acad. Sci. USA* **110**, E2381–E2389 (2013).

51. Sato, Y. *et al.* A Novel enzymatic system against oxidative stress in the thermophilic hydrogen-oxidizing bacterium *Hydrogenobacter thermophilus*. *PLoS One* **7**, e34825 (2012).

52. Anbutsu, H. *et al.* Small genome symbiont underlies cuticle hardness in beetles. *Proc. Natl. Acad. Sci. USA* **114**, E8382–E8391 (2017).

53. Fujisawa, T., Kurosawa, M. & Katagi, T. Uptake and transformation of pesticide metabolites by duckweed (*Lemna gibba*). *J. Agr. Food Chem.* **54**, 6286–6293 (2006).

54. Tago, K., Sato J., Takesa, H., Kawagishi, H. & Hayatsu, M. Characterization of methylhydroquinone-metabolizing oxygenase genes encoded on plasmid in *Burkholderia* sp. NF100. *J. Biosci. Bioeng.* **100**, 517-523 (2005).

55. Chapman, P. J. & Ribbons, D. W. Metabolism of resorcinylic compounds by bacteria: Orcinol pathway in *Pseudomonas putida*. *J. bacteriol.* **125**, 975-984 (1976).

Acknowledgments

We thank Masaki Torimura, Maki Yanagisawa and Haruka Ooi (AIST) for technical assistance and Peter Mergaert (CNRS) for helpful comments on the manuscript. This study was supported by the Institute for Fermentation, Osaka (IFO) to Y.K., the Ministry of Education, Culture, Sports, Science and Technology (MEXT) KAKENHI to Y.K. (20H03303), Y.S. (17H04716), and the JSPS Research Fellowship for Young Scientists to S.J. (201911493).

Author information

Affiliations

Environmental Management Research Institute, National Institute of Advanced Industrial Science and Technology (AIST), Tsukuba Center, Tsukuba, Japan

Yuya Sato & Tomoyuki Hori

Bioproduction Research Institute, AIST, Hokkaido Center, Sapporo, Japan

Seonghan Jang, Hideomi Itoh & Yoshitomo Kikuchi

Graduate School of Agriculture, Hokkaido University, Sapporo, Japan

Seonghan Jang & Yoshitomo Kikuchi

Faculty of Bioresource Sciences, Akita Prefectural University, Akita, Japan

Kazutaka Takeshita

Bioproduction Research Institute, AIST, Tsukuba Center, Tsukuba, Japan

Hideaki Koike

Institute for Agro-Environmental Sciences, National Agriculture and Food Research Organization (NARO), Tsukuba, Japan

Kanako Tago & Masahito Hayatsu

Contributions

Y.S. and Y.K. designed the study. Y.K. and K.Takeshita sequenced and analyzed the whole genome of the symbiont; K.Takeshita investigated plasmid transfer between symbiont strains; Y.S., H.K., and T.H. performed RNA-seq analysis; Y.S., S.J., and H.I. conducted genetic manipulation, infection test, and insecticide exposure test; Y.S. and S.J. measured bactericidal activities of the insecticide and its-degradation product; K.Tago and M.H. conducted HPLC measurement; Y.S. performed LC-ESI-MS; Y.S. and Y.K. wrote the manuscript.

Corresponding author

Correspondence to Yoshitomo Kikuchi <y-kikuchi@aist.go.jp>

Ethics declarations

Competing interests

The authors declare no competing interests.

Figure legends

Fig. 1 | MEP-degrading genes are differentially expressed in cultured and gut-colonizing symbiont cells. a, A male of *Riptortus pedestris* and its dissected gut. An arrow indicates symbiotic organ with numerous crypts. Inset is a fluorescence microscopy image of the crypts infected with a GFP-labelled MEP-degrading symbiont. **b,** MEP-degrading genes encoded in plasmid 2 of the symbiont strain SFA1. **c,** A phylogenetic tree of *mpd* (292 aa), showing frequent horizontal gene transfer of the MEP-degrading gene. See also Supplementary Fig. 3. **d,** Gene expression patterns of the MEP-degrading genes *in vitro* and *in vivo* conditions. Bars indicate standard errors.

Fig. 2 | The first catabolic step of MEP degradation is critical for the symbiont-mediated insecticide resistance. a, Partial MEP degradation pathway and corresponding color changes in wild type SFA1 culture with MEP. **b, c,** Growth rates of wild type SFA1, *mpd* (Δ*mpd*) and *pnpA* gene-deletion mutants (Δ*pnpA1*, Δ*pnpA2*, Δ*pnpA1*/Δ*pnpA2*), and *mpd*-complement mutant (Δ*mpd*/*mpd*$^{+}$) in (**b**) glucose and (**c**) MEP-medium. Photos of culture media (16 h after inoculation) are shown on the right side of (**c**). The medium of Δ*pnpA2* and Δ*pnpA1*/Δ*pnpA2* became yellow because of 3M4N accumulation. **d,** Colonization titers of the wild type and mutant strains in midgut crypts. Symbiont titer between the groups were calculated by qPCR of the *dnaA* gene of the symbiont. Mean and SE of 10 replicates are indicated. ns: statistical non-significance. **e,** Survival rates after MEP-treatment in insects infected with either wild type or mutants. Number of replicates are depicted on the bars. Different letters indicate statistically significant differences ($P < 0.05$).

Fig. 3 | The insecticide-degradation product, 3M4N, is highly bactericidal but efficiently eliminated by the host from the gut symbiotic organ. a, Bactericidal abilities of MEP and 3M4N on cultured SFA1 cells (*in vitro*) and midgut symbiont cells (*in vivo*). **b,** Effects of 3M4N on crypt-colonizing symbiont population in the bean bug gut. Insects infected with SFA1(wt) and its Δ*pnpA1*/Δ*pnpA2* mutant were fed on soybean seeds and either DW or DW containing 5 mM 3M4N. Symbiont titer between the groups were compared by qPCR of the *dnaA* gene of the symbiont. Mean and SE of 10 replicates are indicated. ns indicates statistical insignificance. Fluorescent microscopy images of the midgut crypts colonized by GFP-labelled SFA1 (wt) and Δ*pnpA1*/Δ*pnpA2* are shown on top of the bar graph. **c,** MEP-degradation and 3M4N production in the gut harboring the MEP-degrading symbiont SFA1. The degradation rate is compared with that of SFA1 grown in culture. **d,** A schematic illustration of the MEP-detoxification governed by host-symbiont mutualistic interactions.

Supplementary Information

Insecticide resistance by a host-symbiont reciprocal detoxification

Sato, Jang *et al.*

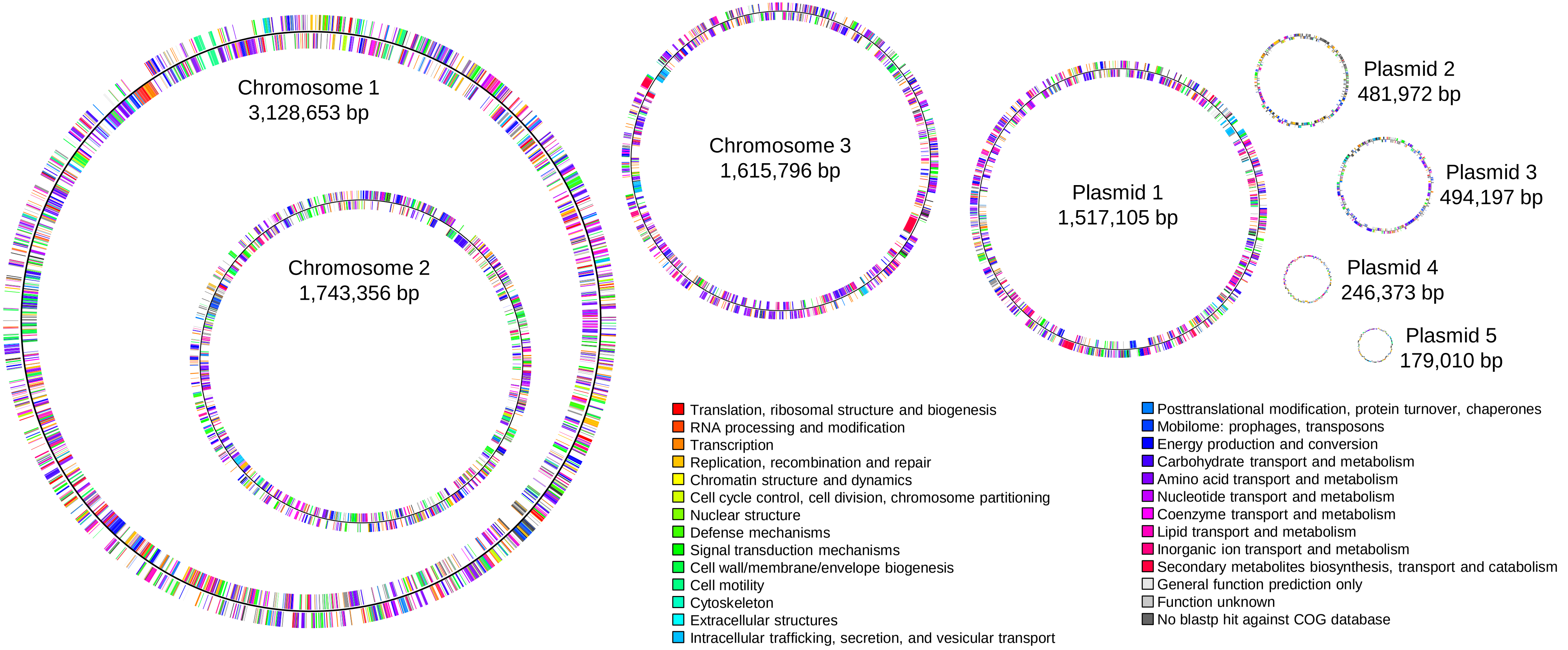

Supplementary Fig. 1 | The genome of *Burkholderia* symbiont strain SFA1. On the CDS circle, colors indicate functional categories as shown in the figure.

a

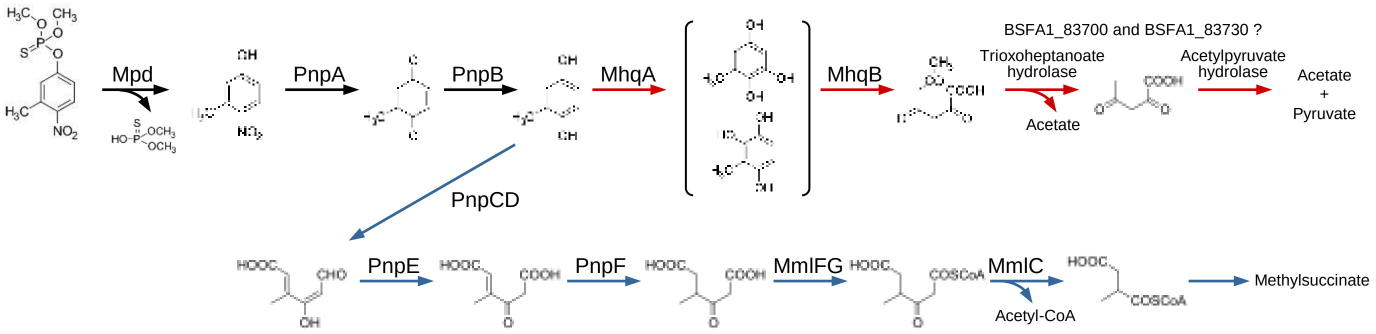

b

Gene	ID	Chromosome / Plasmid number	Gene expression level (TPM) In vitro: Citrate	In vitro: MEP	In vivo (24 h): Control	In vivo (24 h): 0.2 μM MEP	In vivo (24 h): 2 μM MEP	In vivo (72 h): Control	In vivo (72 h): 0.2 μM MEP
mpd	BSFA1_82960	Plasmid 2	6759	2718	1678	2032	1294	1467	1188
pnpA1	BSFA1_81620	Plasmid 2	32	9045	39	8	30	29	13
pnpA2	BSFA1_81600	Plasmid 2	54	10748	51	50	40	39	65
pnpB	BSFA1_81610	Plasmid 2	60	13924	78	10	103	44	46
mhqA	BSFA1_83720	Plasmid 2	67	14623	63	14	54	30	34
mhqB	BSFA1_83710	Plasmid 2	37	6784	99	13	60	26	26
hydrolase	BSFA1_83700	Plasmid 2	10	3340	24	42	16	0	33
5-oxopent-3-ene-1,2,5-tricarboxylate decarboxylase	BSFA1_83730	Plasmid 2	40	13617	88	77	38	31	5
pnpC	BSFA1_81630	Plasmid 2	42	12775	113	71	74	42	39
pnpD	BSFA1_81650	Plasmid 2	41	3759	33	27	0	13	30
pnpE	BSFA1_81660	Plasmid 2	4	196	8	21	11	19	12
pnpF	BSFA1_81670	Plasmid 2	9	194	30	11	65	18	0
mmlC	BSFA1_71580	Plasmid 1	4	9	2	16	19	0	4
mmlF	BSFA1_11210	Chromosome 1	171	81	54	31	29	65	71
mmlG	BSFA1_11220	Chromosome 1	182	139	9	144	37	57	64

c

Gene	ID	Chromosome / Plasmid number	Gene expression level (TPM) In vitro: Citrate	In vitro: MEP	In vivo (24 h): Control	In vivo (24 h): 0.2 μM MEP	In vivo (24 h): 2 μM MEP	In vivo (72 h): Control	In vivo (72 h): 0.2 μM MEP
pnpC	BSFA1_47960	Chromosome 3	26	48	71	52	16	46	23
pnpD	BSFA1_47970	Chromosome 3	14	23	21	10	32	21	5
pnpE	BSFA1_47980	Chromosome 3	7	10	52	12	14	11	17
pnpF	BSFA1_47990	Chromosome 3	44	35	41	10	15	11	15
pnpF	BSFA1_53550	Chromosome 3	5	3	38	10	0	16	10

Common Mhq pathway Pnp pathway

Gene expression (TPM) 20,000 2,000 0

Supplementary Fig. 2 | MEP-degradation pathways inferred from the SFA1 genome. a, The Pnp pathway (blue arrows) was reported in the MEP-degrading *Pseudomonas* sp. WBC-3[28] and the Mhq pathway (red arrows) was reported in the MEP-degrading *Burkholderia* sp. NF100[54]. **b,** Gene expression levels of the MEP-degrading genes. All expression values are mean from 3 replicates. Note that the Mhq pathway is mainly used in SFA1 when MEP is added as the sole carbon source in culture. In the Mhq pathway, the last two steps of the ring cleavage process are still undetermined. Considering a previous report[55] and our RNA-seq data, BSFA1_83700 and BSFA1_83730, which are adjacent to the *mhqA* and *mhqB* genes, are likely involved in the ring cleavage process. Low expression levels (transcripts per million [TPM] values <177) of *mmlC* (BSFA1_71580 encoding 4-methyl-3-oxoadipyl-CoA thioesterase, Chromosome 1) and *mmlFG* (BSFA1_11210 and BSFA1_11220 encoding 4-methyl-3-oxoadipate CoA-transferase alpha- and beta-subunit, respectively; Plasmid 1) suggested that they are not involved in the MEP degradation. **c,** *pnpC–F* homologs are also found in the chromosome, but their expression levels are low.

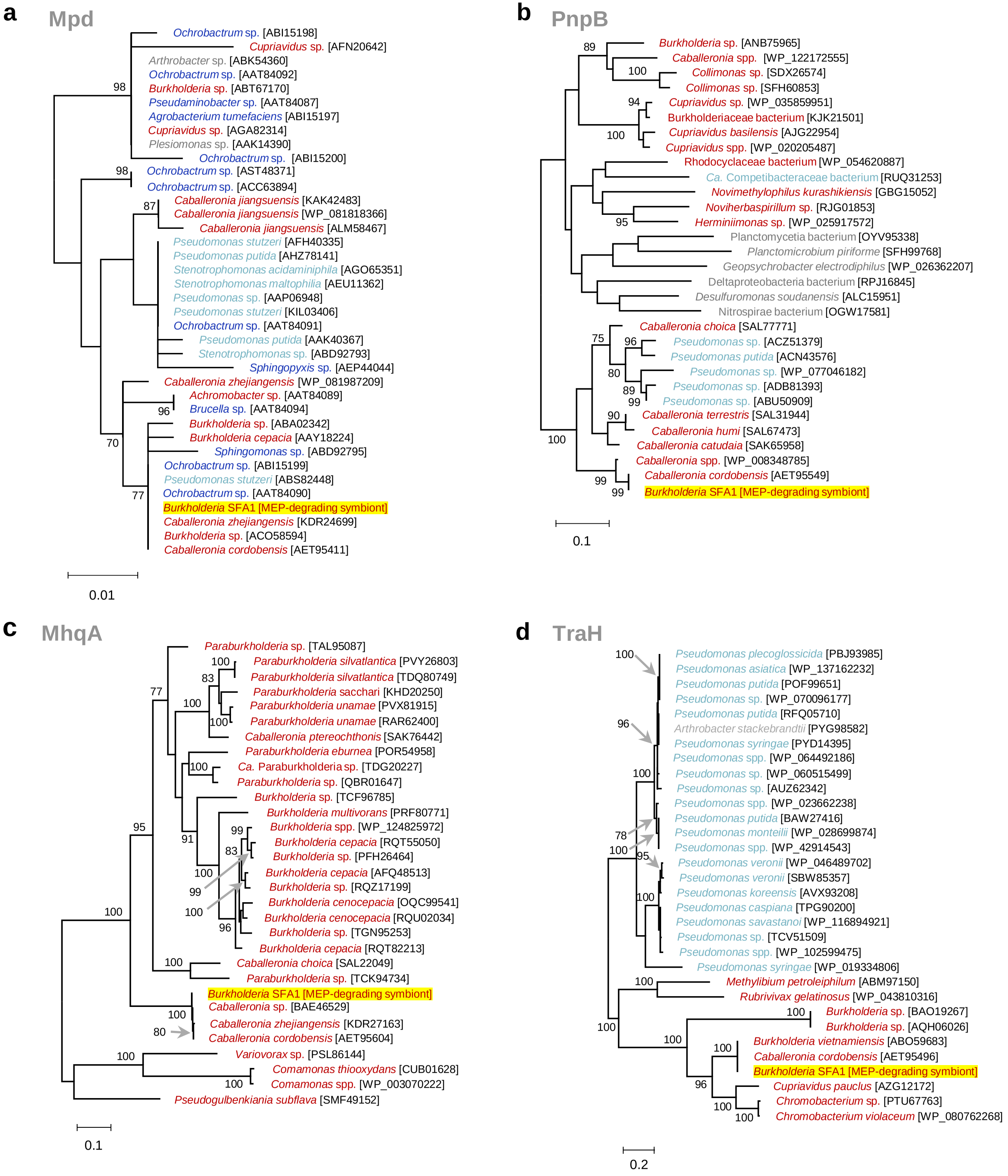

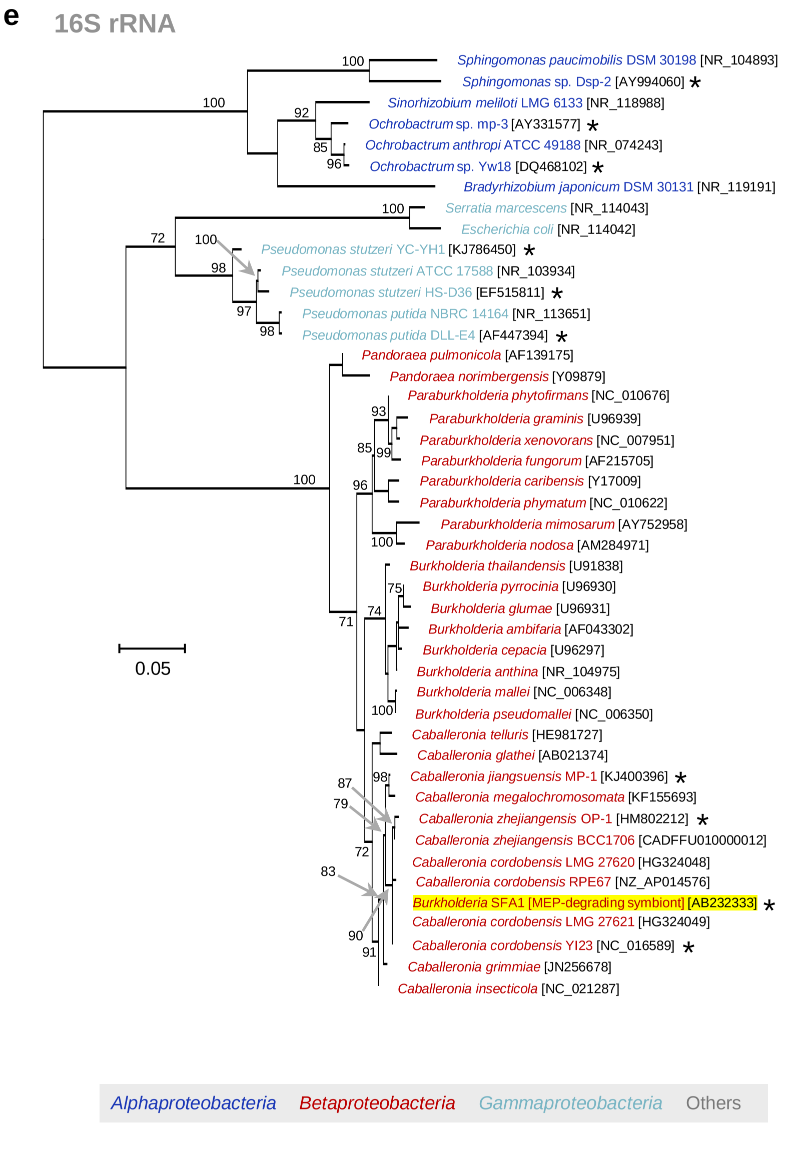

Supplementary Fig. 3 | Phylogenetic trees of MEP-degrading genes. ML trees with bootstrap values (> 70%) are shown. **a,** *mpd*; **b,** *pnpB*; **c,** *mhqA*; **d,** a plasmid transfer gene (*traH*). **e,** Phylogenetic relationship of the MEP-degrading species was estimated by 16S rRNA gene sequences, wherein MEP-degrading species/strains that carry *mpd* are indicated by asterisks. These phylogenetic analyses strongly suggest that the MEP-degrading genes and/or the plasmid itself transmit with a high frequency.

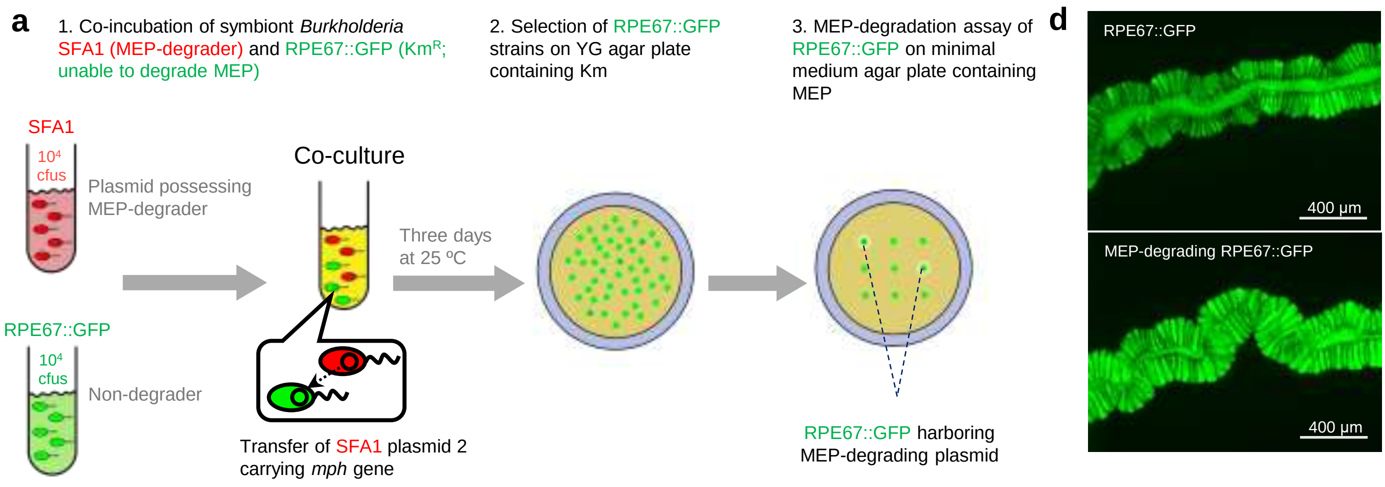

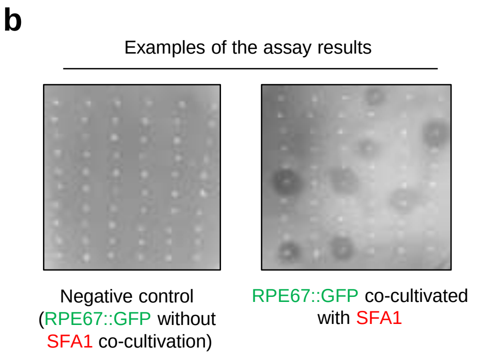

c

Batch	Ratio of MEP-degrading RPE67::GFP (%)[a]
#1	5/96 (5.2%)
#2	6/96 (6.3%)
#3	11/96 (11.5%)
#4	10/96 (10.4%)
#5	9/96 (9.4%)

[a]Ratio of MEP-degrading bacteria was calculated by [The number of MEP-degrading RPE67::GFP colonies / Total number of RPE67::GFP colonies]

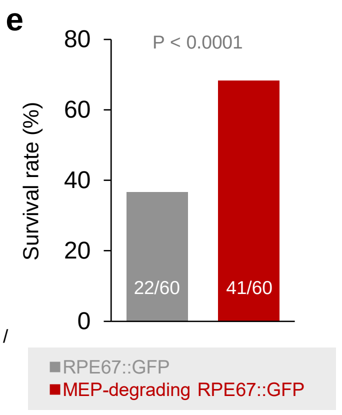

Supplementary Fig. 4 | Horizontal transmission of the MEP-degrading plasmid between *Burkholderia* symbiont strains. **a,** Analysis of the horizontal transmission of the MEP-degrading ability between *Burkholderia* symbiont strains under laboratory condition. SFA1 was co-cultured with RPE67, a non-degrading strain whose chromosome was tagged by GFP, and after overnight incubation, the inter-strain transmission of the MEP-degrading ability from SFA1 to the GFP-labelled RPE67 was investigated by halo-formation on agar plates containing emulsified MEP. **b,** Examples of the assay showing transmission of MEP-degrading ability. **c,** Table of the transmission rate of MEP-degrading ability in five trials. **d,** A newly-emerged MEP-degrading RPE67 derivative colonizes the midgut crypts. **e,** The survival rates after MEP treatment in insects infected with either the non-degrading symbiont (RPE67) or the newly-emerged MEP-degrading symbiont (MEP-degrading RPE67). The method is the same as the one in Figure 2e. Statistically analyzed by Fisher's exact test.

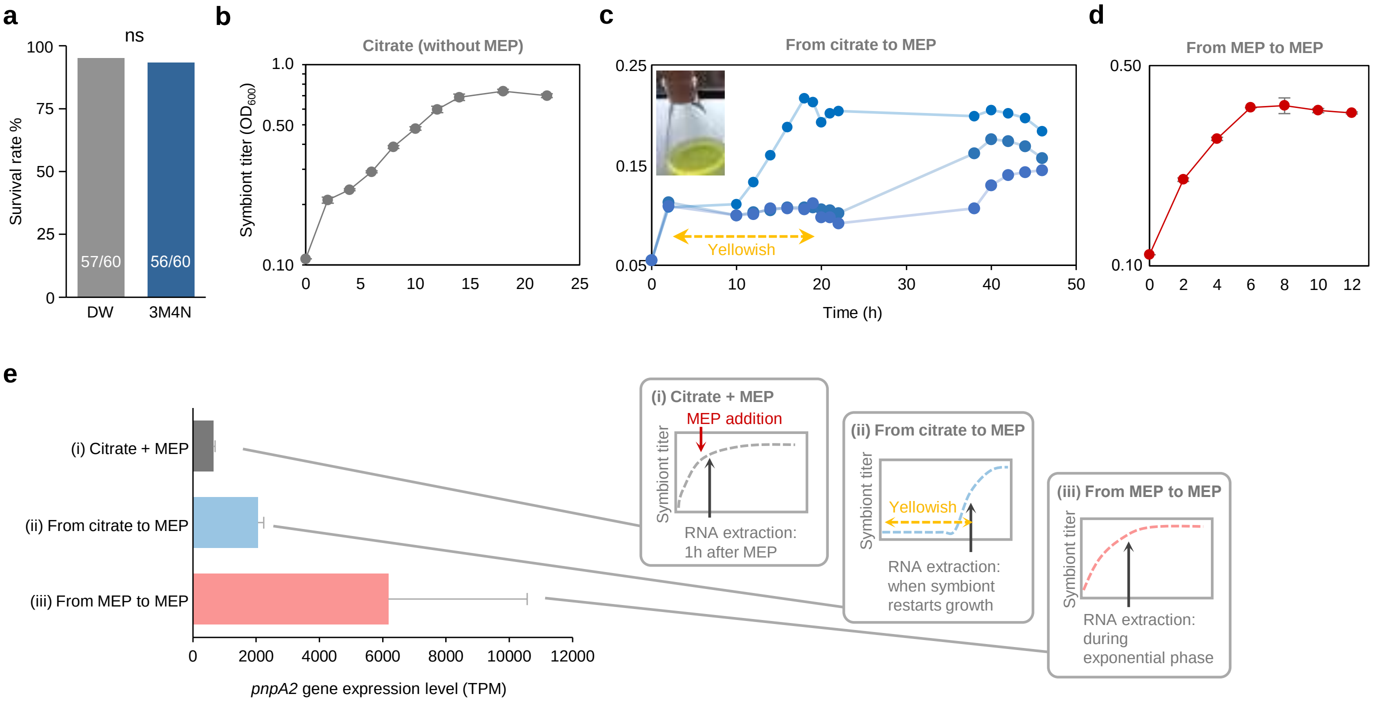

Supplementary Fig. 5 | Effects of 3M4N on the bean bug and the symbiotic bacteria. a, Survival rate of bean bugs that are fed on 3M4N. The survival rates were not significantly different between DWA-fed and 3M4N-fed insects, indicating 3M4N is non-toxic to the insect. Insects were reared on soybean seeds and either of DWA or 3M4N solution (DWA containing 5 mM 3M4N) for 2 days, and their survival was monitored for 5 days. **b** to **d,** Effects of 3M4N on the symbiotic bacteria: growth delay of SFA1 in MEP medium after pre-culture in citrate medium. **b,** Growth of SFA1 in minimal medium containing 0.1% citrate (citrate medium). **c,** Growth of SFA1 in minimal medium containing 1.0 mM of MEP (MEP medium) after pre-culture in the citrate medium. The medium became yellowish because of 3M4N accumulation (see inset picture) and the growth was delayed. After 10-20 h delay, bacterial growth was recovered because of the *pnpA2* expression was induced by 3M4N. The growth curves of three independent cultures are shown. **d,** Growth of SFA1 in MEP medium after pre-culture in MEP medium. No growth delay was observed. **e,** Gene expression of *pnpA2* during cultivation with MEP investigated by RNA-seq. Culture conditions: (i) SFA1 was cultured in minimal medium containing citrate and analyzed one hour after MEP addition; (ii) cells were transferred from the citrate medium to MEP medium, wherein growth delay was observed, and analyzed after yellow color of MEP medium started to disappear; (iii) cells were transferred from MEP medium to newly-prepared MEP medium, wherein no growth delay was observed. The values are mean from 2 replicates. The bars denote standard error.

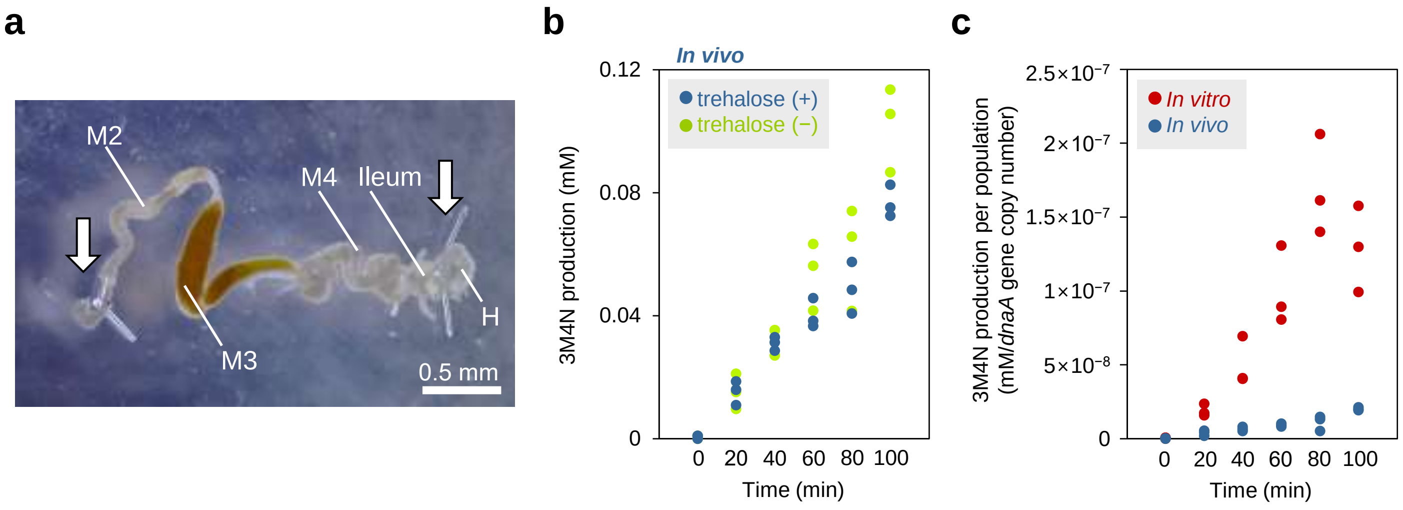

Supplementary Fig. 6 | Detoxification of MEP and production of 3M4N by dissected midguts. a, A dissected gut of a 5th instar insect infected with SFA1, closed at both ends with a 0.2 mm polyethylene fishline knot. M2, midgut second section; M3, midgut third section; M4, midgut fourth section (symbiotic organ); H, hindgut. **b,** 3M4N production in the gut harboring the MEP-degrading symbiont SFA1 when tissues were incubated in MEP-solution containing 250 mM trehalose. 3M4N production was not significantly different between with and without trehalose for each time point (Mann–Whitney *U* test after Bonferroni correction). The *in vivo* incubation data without trehalose is from Fig. 3c. **c,** MEP-degrading (3M4N production) rate per single SFA1 cell. The degradation rates shown in Fig. 3c were divided by symbiont cell numbers estimated by qPCR of the *dnaA* gene.

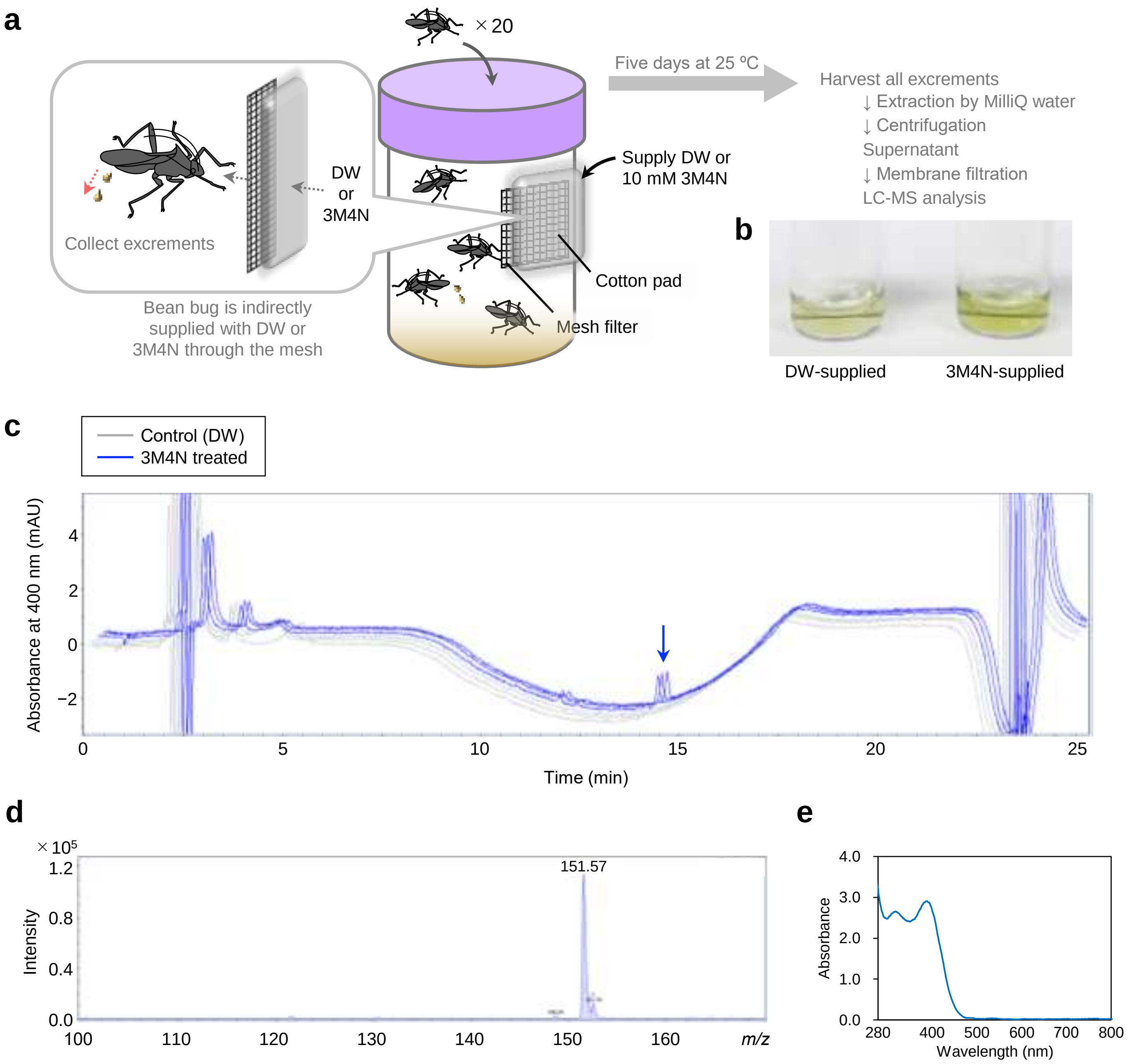

Supplementary Fig. 7 | Detection of 3M4N from feces. a, An insect rearing system for feeding 3M4N and collecting feces. Insects were fed with DW or DW containing 10 mM 3M4N in a plastic container, where the solution supplier was covered by 0.5 mm mesh so that insects were able to drink the solution by probing with their proboscis but did not directly touch the solution by their legs or body. **b,** Color of isolated feces. Feces isolated from the 3M4N-fed group are more yellowish in color. Feces were dissolved in DW adjusted to pH 8.0. **c,** Chromatogram of the feces extracts analyzed by HPLC. Monitoring of absorbance at 400 nm, which corresponds to 3M4N specific absorbance, detected a clear peak at 14.2 min only in the 3M4N-fed group. Triplicate chromatograms of the DW- and 3M4N-fed groups are stacked. **d,** Mass spectrum of the HPLC 14.2 min fraction in negative mode. The *m/z* of 151.57 is identical to that of 3M4N standard (*m/z* = 151.53). The mass and other spectral data of 3M4N standard are available from Spectral Database for Organic Compounds (https://sdbs.db.aist.go.jp/sdbs/cgi-bin/direct_frame_top.cgi). **e,** UV-V is absorbance spectrum of 1 mM 3M4N. A large peak was observed near 390 nm.

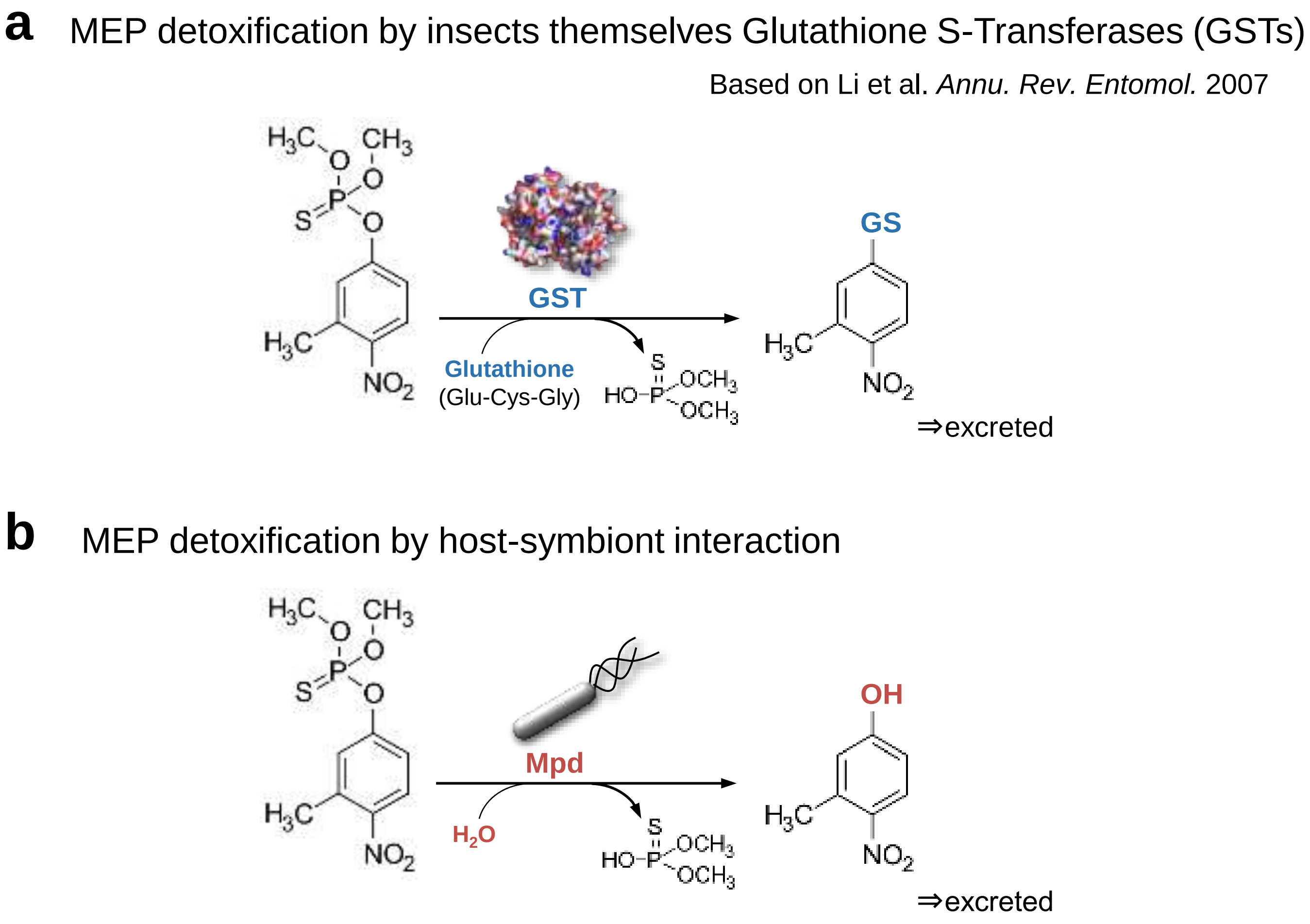

Supplementary Fig. 8 | The analogy between the insect-encoded and the symbiont-mediated detoxification systems. The symbiont-mediated MEP-detoxification mechanism revealed here is analogous to the detoxification mechanism reported in MEP-resistant insects where glutathione S-transferases (GSTs) are highly up-regulated[26]. GSTs detoxify MEP by conjugating glutathione and the symbiont Mpd hydrolyzes MEP. The products of these enzymes, 5-*S*-glutathionyl-1-methyl-2-nitrobenzene and 3M4N, respectively, are immediately eliminated from insect cells and tissues.

Supplementary Table 1 | Primers used in this study

Primer	Sequence[a] (5' → 3')	Restriction enzyme	Usage
mph-LF-EcoRI	CTGGAATTCCAGGTAGGCCAGCACCACAC	EcoRI	*mph* deletion
mph-LR	*GGCACGAAACCCAGCAAGCG*GTTCTTCAGG		
mph-RF	*CGCTTGCTGGGTTTCGTGCC*GGTGAACTAC		
mph-RR-HindIII	TCGAAGCTTTGCCCTACATCTCCAAGGTC	HindIII	
pnpA1-LF-EcoI	TTGGAATTCAACTGCTGATGCAGATACGG	EcoRI	*pnpA1* deletion
pnpA1-LR-XbaI	*GATTGTGTCTAGAGTCTCCA*TCTTGTGTCTC	XbaI	
pnpA1-RF-XbaI	*TGGAGACTCTAGACACAATC*GCTCAATTCTC	XbaI	
pnpA1-RR-HindIII	AATAAGCTTGTCCGACTGCGAAACGTC	HindIII	
pnpA2-LF-BamHI	TGAGGATCCCACGGCGAATCAGCTTGTAG	BamHI	*pnpA2* deletion and *pnpA1*/*pnpA2* double deletion
pnpA2-LR-XbaI	*ATCAGCATCTAGAGTTTCCA*TTTTGCAGTC	XbaI	
pnpA2-RF-XbaI	*TGGAAACTCTAGATGCTGAT*GCAGATACGG	XbaI	
pnpA2-RR-HindIII	GTGAAGCTTACTTGAACGCGACATTGCTG	HindIII	
mph-ex-LF-EcoRI	GCCGAATTCCTGCAGCTGCTGCGC	EcoRI	*mph* complementation
mph-ex-LR-BamHI	AGCGGATCCTCAGGGGCATGGTG	BamHI	
mph-ex-RF-HindIII	AAGAAGCTTCGTTTCGTGCCGGTG	HindIII	
mph-ex-RR-HindIII	GCAAAGCTTGGCGGCGCTCAAGGC	HindIII	
mph-ex-F-BamHI	AGGGGATCCAGGATGTAGGAC	BamHI	
mph-ex-R-fsn-kmr	*ATAAAACCGCCTAACGGTGC*GGGCAAG		
kmr-F-fsn-mph	*GCACCGTTAGGCGGTTTTAT*GGACAGCAAG		
kmr-R-BamHI	CTAGGGATCCCCAGAGTCC	BamHI	
tcr-F-XbaI	ATCTCTAGAGAACTCTCTCCCAAAG	XbaI	Tc resistance cassette
tcr-R-XbaI	ACGGTCTAGATTGTTGTATAAGTG	XbaI	
kmr-F-XbaI	GGTTCTAGAGACAGCAAGCGAAC	XbaI	Km resistance cassette
kmr-R-XbaI	AACTCTAGAGTCCCGCTCAGAAG	XbaI	

[a]In the sequences, restriction sites are indicated by underlines, and corresponding restriction enzymes are described right column. Italic indicates complement sequence for the pair primer.

Reviewers' Comments:

Reviewer #1:
Remarks to the Author:
I would like to thank the authors for their careful revisions. My comments have been addressed in a satisfactory manner. The only point I remain somewhat concerned about is the failure to detect MEP and 3M4N in the feces of MEP-fed insects, as indicated in the rebuttal letter. This casts some doubt on the excretion of 3M4N via the insect feces as concluded in the manuscript. In particular, the sentence in l. 188-190 may be overstated: "When feces of bean bugs fed with water containing 3M4N were analyzed by LC-MS, the compound was detected in the feces (Supplementary Fig. 7), suggesting that 3M4N is not further metabolized in the bean bug host but excreted in unchanged form." While I do not think this is a major problem in the study, I recommend to tone down this conclusion a bit in light of the finding of MEP and 3M4N not being detected in the feces of MEP-fed insects, which are not reported in the manuscript.
Nonetheless, I would like to commend the authors again on a fascinating and exciting study that will certainly be a very valuable contribution to the literature on symbiont-assisted detoxification in insects.

Reviewer #2:
Remarks to the Author:
The revisions address all of the points of the reviewers, and the manuscript is a valuable contribution to the areas of host-symbiont interactions and insecticide resistance.

Reviewer #3:
Remarks to the Author:
I am happy with this version as it is.
